# Design, synthesis and biological evaluation of novel biphenylsulfonamide derivatives as selective AT_2_ receptor antagonists

**DOI:** 10.3389/fchem.2022.984717

**Published:** 2022-08-26

**Authors:** Danhui Wang, Wenjie Zhao, Zuzhi Zhang, Yanchun Zhang, Jiaming Li, Weijun Huang

**Affiliations:** ^1^ College of Pharmacy, Anhui University of Chinese Medicine, Hefei, China; ^2^ Anhui Province Key Laboratory of Chinese Medicinal Formula, Hefei, Anhui, China

**Keywords:** benzenesulfonamide derivatives, AT2 receptor, antagonist, selective, drug design

## Abstract

A novel series of benzenesulfonamide derivatives that selectively act on the AT_2_ receptor have been designed and synthesized. The binding affinity and functional activity were evaluated by radio-ligand binding analysis and cell neurite outgrowth assay, respectively. The compounds **8d**, **8h**, **8i**, **8j**, **8l**, and **9h** exhibited moderate selectivity and affinity for the AT_2_ receptor. Among them, **8j** exhibited agonist activity and **8l** displayed similar selectivity to the AT_2_ receptor with **PD123,319**. Molecular docking was carried out to analyze the binding mode and binding site between the compound and the AT_2_ receptor to provide a reference for further development.

## 1 Introduction

Benzenesulfonamide derivatives have a variety of biological activities, such as being anti-microbial and anti-tumor, protecting against cardiovascular disease, offering resistance to diabetes, and so on*.* The AT_2_ receptor, which is one of the subtypes of Angiotensin II receptor, is rarely expressed in normal tissue. In certain pathological conditions, the expression of the AT_2_ receptor was significantly up-regulated, such as myocardial infarction, vascular injury, cerebral ischemia, and so on (Carey, 2005; Juillerat-Jeanneret, 2020). A growing number of studies have suggested that AT_2_ receptor antagonists could alleviate peripheral neuropathic pain by blocking neuronal excitability (Hein et al., 1995).

**FIGURE 1:**
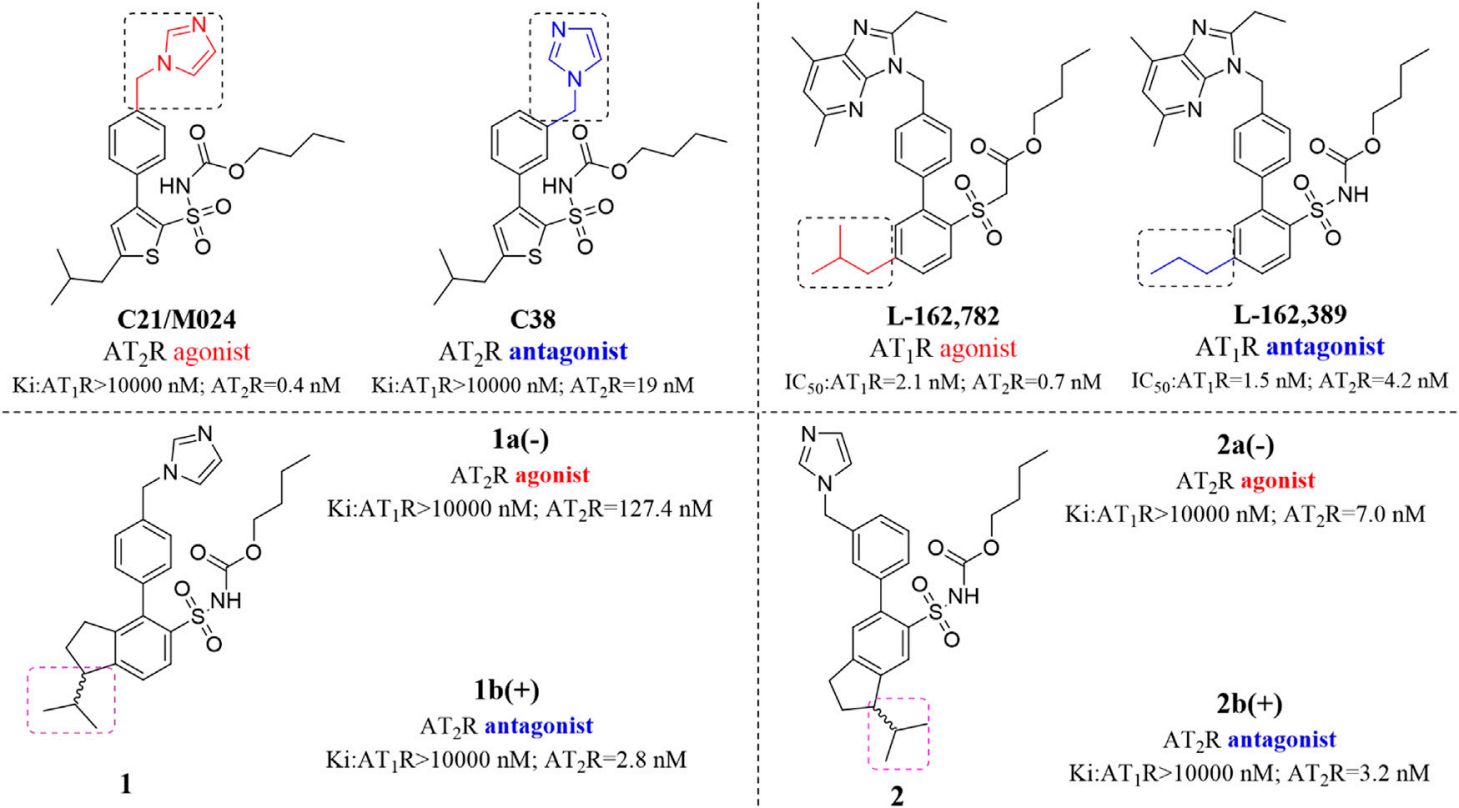
Small changes in the structure of the compound lead to the change of functional activity.

**FIGURE 2:**
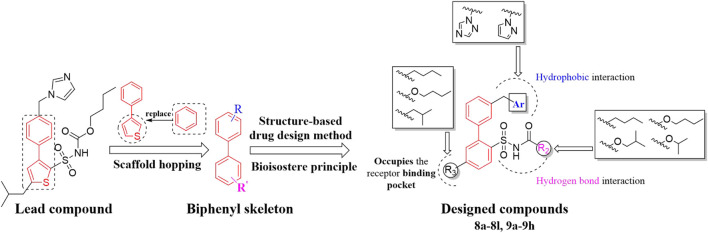
The design of target compounds.

**C21/M024** is the first selective non-peptide AT_2_ receptor agonist (Wan et al., 2004). It was observed that a migration of the methylene imidazole substituent resulted in the compound with AT_2_ receptor antagonist activity (Murugaiah et al., 2012; Wallinder et al., 2019) (Figure 1). To develop an AT_2_ receptor antagonist with novel scaffold, a series of heterocyclic substituted benzenesulfonamide derivatives were designed based on the principle of scaffold hopping and bioelectronic isosteric (as shown in Figure 2). The binding affinity and functional activity were evaluated by the recognized assay.

## 2 Results and discussion

### 2.1 Chemistry

**SCHEME 1:**
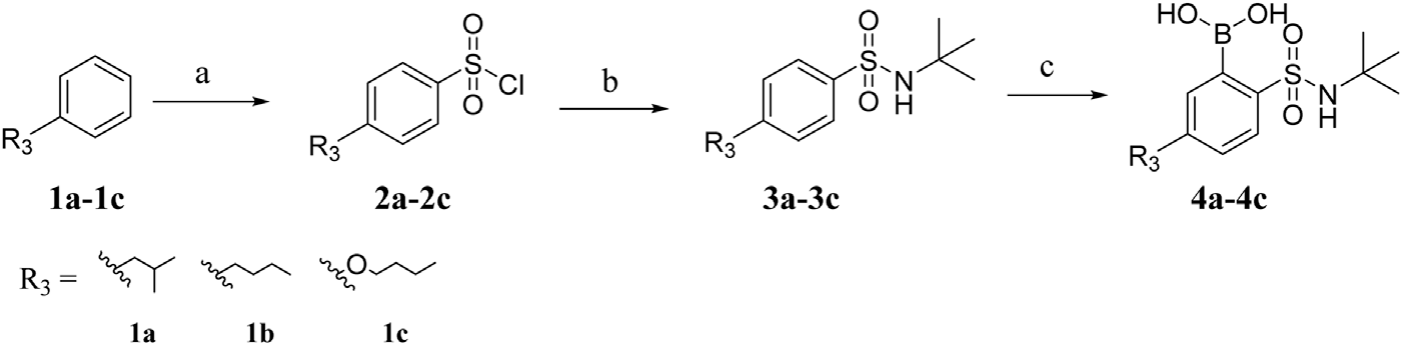
Synthesis of intermediates **4a-4c**.

**SCHEME 2:**
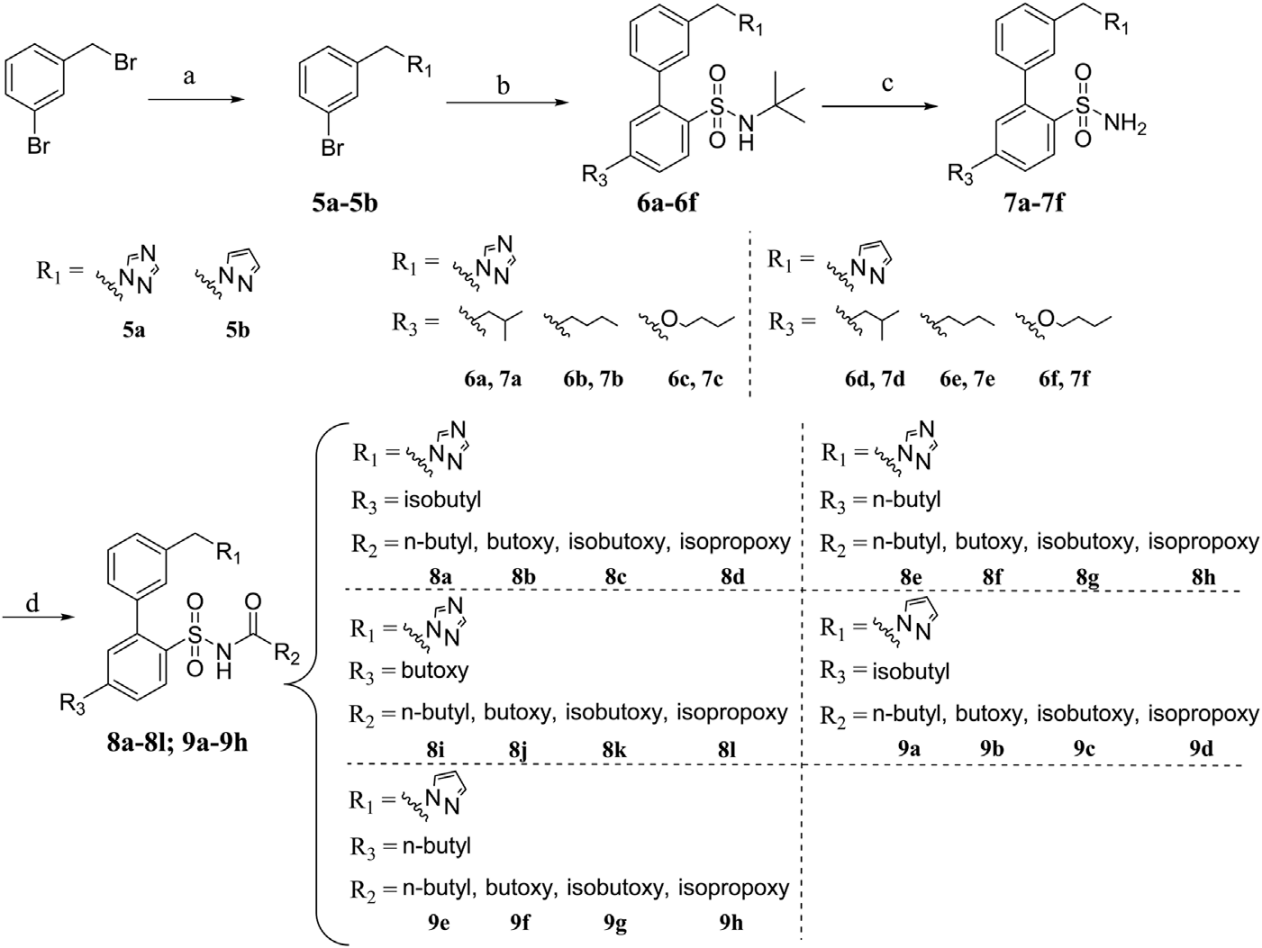
Synthesis of target compounds **8a-8l, 9a-9h**.

The boric acid intermediates **4a**-**4c** were obtained according to the synthetic route shown in Scheme 1 (Liu et al., 2013). The triazole and pyrazole was substituted to produce **5a** and **5b,** respectively. Then, the target compounds (**8a-8l** and **9a-9h**) were synthesized by Suzuki coupling, amino deprotection, and esterification (as shown in Scheme 2).

### 2.2 Biological evaluation

#### 2.2.1 Binding assay

The target compounds were evaluated for AT_1_ receptor binding affinity by displacement of [^125^I]-Ang II from the AT_1_ receptor in rat liver membranes. The radio-iodinated AT_2_ receptor selective ligand [^125^I]-CGP42112 was used to study AT_2_ receptor binding affinity in HEK-293 cells (HEK293-hAT_2_R) (Dudley et al., 1990; Whitebread et al., 1991). All of above methods were widely accepted and used to evaluate the binding activity of AT_1_ and AT_2_ receptors. **PD-123,319** is often used as a tool compound in the analysis of AT_2_ receptor antagonists (Blankley et al., 1991). The results are summarized in Table 1.

**TABLE 1:** The binding affinity and selectivity of the compound to the AT_2_ receptor.

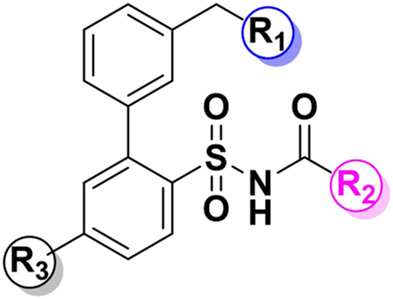					
Compound	R_1_	R_2_	R_3_	Ki (nM)	
				AT_1_R	AT_2_R
**8a**	1,2,4-triazole	*n*-Bu	*i*-Bu	629.4 ± 1.1	873.7 ± 1.1
**8b**	1,2,4-triazole	OBu-*n*	*i*-Bu	846.1 ± 1.17	273.5 ± 1.2
**8c**	1,2,4-triazole	OBu-*i*	*i*-Bu	1037 ± 1.1	322 ± 1.24
**8d**	1,2,4-triazole	OPr-*i*	*i*-Bu	1786 ± 1.37	115.9 ± 1.18
**8e**	1,2,4-triazole	*n*-Bu	*n*-Bu	505.2 ± 1.4	753.9 ± 1.1
**8f**	1,2,4-triazole	OBu-*n*	*n*-Bu	1425 ± 1.12	252.7 ± 1.2
**8g**	1,2,4-triazole	OBu-*i*	*n*-Bu	945.5 ± 1.22	347.5 ± 1.36
**8h**	1,2,4-triazole	OPr-*i*	*n*-Bu	1505 ± 1.11	82.9 ± 1.19
**8i**	1,2,4-triazole	*n*-Bu	OBu-*n*	1250 ± 1.12	199.6 ± 1.17
**8j**	1,2,4-triazole	OBu-*n*	OBu-*n*	889.3 ± 1.24	620.1 ± 1.27
**8k**	1,2,4-triazole	OBu-*i*	OBu-*n*	775 ± 1.08	712.5 ± 1.17
**8l**	1,2,4-triazole	OPr-i	OBu-n	2005 ± 1.16	56.59 ± 1.16
**9a**	pyrazole	*n*-Bu	*i*-Bu	1264 ± 1.14	1152 ± 1.1
**9b**	pyrazole	OBu-*n*	*i*-Bu	989.7 ± 1.08	1027 ± 1.15
**9c**	pyrazole	OBu-*i*	*i*-Bu	1027 ± 1.08	916.2 ± 1.3
**9d**	pyrazole	OPr-*i*	*i*-Bu	1549 ± 1.11	74.09 ± 1.16
**9e**	pyrazole	*n*-Bu	*n*-Bu	363.9 ± 1.28	992.9 ± 1.26
**9f**	pyrazole	OBu-*n*	*n*-Bu	1310 ± 1.11	467.9 ± 1.19
**9g**	pyrazole	OBu-*i*	*n*-Bu	1212 ± 1.12	513.3 ± 1.18
**9h**	pyrazole	OPr-*i*	*n*-Bu	1723 ± 1.07	138 ± 1.34
**PD-123,319** (Blankley et al., 1991)	-	-	*-*	2758 ± 1.09	34 ± 1.15

The affinity of **8a**, **8e**, **8j**, **8k**, **9a**, **9b**, and **9c** to the AT_1_ and AT_2_ receptors was lower and with rare selectivity. The selectivity of **8l** to the AT_2_ receptor was modest, which binding force to the AT_2_ receptor is equivalent to **PD-123,319** [*Ki*: AT_2_R = 34 nM (Blankley et al., 1991)].

#### 2.2.2 Function evaluation assay

The compounds (i.e., **8d**, **8h**, **9h**, **8i**, **8j**, **8k**, and **8l**) with higher-affinity and which were structurally diverse were selected for further function evaluation by neurite outgrowth assay with NG108-15 cells, which is accepted as an AT_2_ receptor function assay (Buisson et al., 1992; Gasparo, 1996). The ratio of cell neurite outgrowth was used to evaluate, and the details are shown in the Supplementary Material.

**FIGURE 3:**
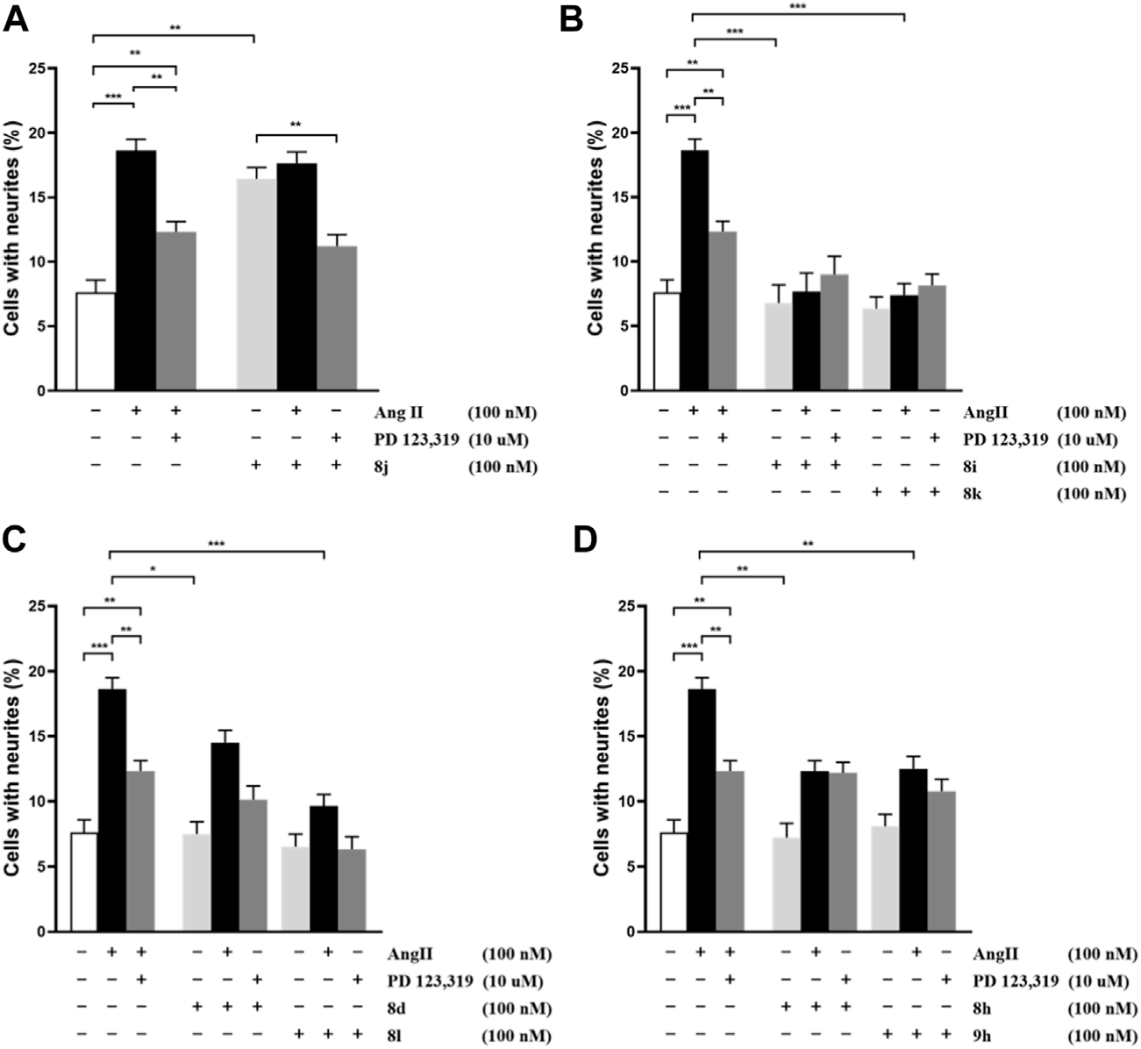
Effect of compounds **8j**, **8i**, **8k**, **8d**, **8l**, **8h**, and **9h** on neurite outgrowth in NG108-15 cells. The number of cells with neurites was expressed as the percentage of the total number in the micrographs (at least 400 cells per well plate according to the experimental requirements). The results are significant according to two-way ANOVA: ***, *p* < 0.001; **, *p* < 0.01; *, *p* < 0.05; no significant difference is not shown.

Ang II can induce the outgrowth of cell neurite at a ratio of nearly 20% (shown in Figure 3A). When co-incubated with AT_2_ receptor antagonist **PD-123,319**, the outgrowth of **Ang II** induce ratio were decreased. This means the outgrowth of cell neurite might be induced by the AT_2_ receptor. As shown in Figures 3B–D **,** the outgrowth of the cell neurite was significantly less than 20% when incubated with the evaluated compounds alone, which means that the compounds inhibit the **Ang II** effect. The cell neurite outgrowth was slightly increased when the cells were inhibited by **PD-123,319**, which means that the inhibition of Ang II was induced by the AT_2_ receptor. Accordingly, **8i**, **8k**, **8d**, **8l**, **8h**, and **9h** had antagonistic activity against the AT_2_ receptor. The cell outgrowth ability of **8j** were blocked by **PD-123,319**, which means that **8j** exerted AT_2_ receptor agonistic activity (shown in Figure 3A). These results were found to be significant according to a two-way analysis of variance (ANOVA).

### 2.3 Molecular docking studies

The binding affinity with the AT_2_ receptor and the functional activity of selected compounds (i.e., **8j**, **8i**, **8k**, **8d**, **8l**, **8h**, and **9h)** are summarized in Table 2. The pyrazole group may provide an opportunity for binding to the AT_1_ receptor. Minor changes of the sulfonamide substituents of ligands not only affect the selectivity between compounds and receptors but also affect the functional activity of compounds.

**TABLE 2:** Differences in structure, affinity, and functional activity of compounds.

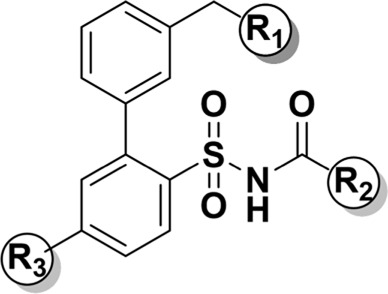						
Compound	R_1_	R_2_	R_3_	Ki (nM)		Function
				AT_1_R	AT_2_R	
**8d**	1,2,4-triazole	OPr-*i*	*i*-Bu	1786 ± 1.37	115.9 ± 1.18	Antagonist
**8h**	1,2,4-triazole	OPr-*i*	*n*-Bu	1505 ± 1.11	82.9 ± 1.19	Antagonist
**8i**	1,2,4-triazole	*n*-Bu	OBu-*n*	1250 ± 1.12	199.6 ± 1.17	Antagonist
**8j**	1,2,4-triazole	OBu-*n*	OBu-*n*	889.3 ± 1.24	620.1 ± 1.27	Agonist
**8k**	1,2,4-triazole	OBu-*i*	OBu-*n*	775 ± 1.08	712.5 ± 1.17	Antagonist
**8l**	1,2,4-triazole	OPr-i	OBu-n	2005 ± 1.16	56.59 ± 1.16	Antagonist
**9h**	pyrazole	OPr-*i*	*n*-Bu	1723 ± 1.07	138 ± 1.34	Antagonist

**FIGURE 4:**
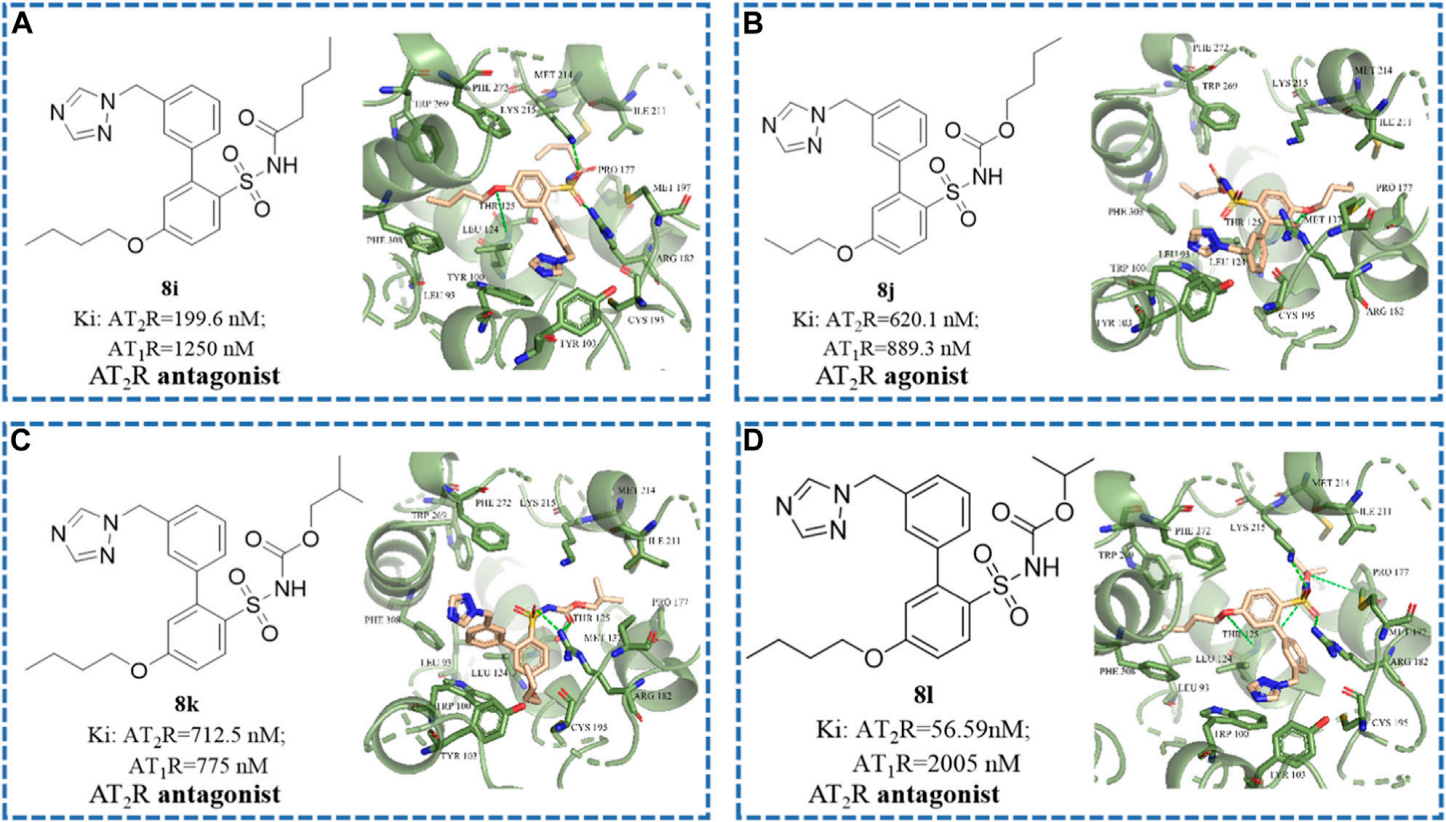
The structural formula and docking results of compounds **(A) 8i**, **(B) 8j**, **(C) 8k**, and **(D) 8l**. The green dotted line indicates hydrogen bond interaction.

To better acquire comprehension of the relationship of structure modification and affinity and activity, and to elucidate the key binding sites where compounds retain antagonistic activity, molecular docking studies were implemented by utilizing SYBYL software (Porrello et al., 2009; Sallander et al., 2016; Zhang et al., 2017; Connolly et al., 2019). As shown in Figures 4B,D, the disappearance of hydrogen bond interaction between sulfonamide side chain and receptor protein not only reduced the affinity between the compound and the AT_2_ receptor by 100 times but also changed the functional activity from antagonist to agonist. As shown in Figures 4A,D, when the isopropoxy side chain of the sulfonamide group of **8l** is replaced with n-butyl (compound **8i**), the n-butyl of the sulfonamide group will competitively extend to the hydrophobic pocket.

We speculated that the Thr 125, Lys 215, Arg 182, and Met 137 residues of the AT_2_ receptor may be the key binding sites for binding affinity. The additional hydrogen bond formed by residues Lys 215, Arg 182, and Pro 177 with the alkyl chain on the sulfonamide group may be the key binding sites for the antagonistic activity of the compound.

## 3 Discussion

**FIGURE 5:**
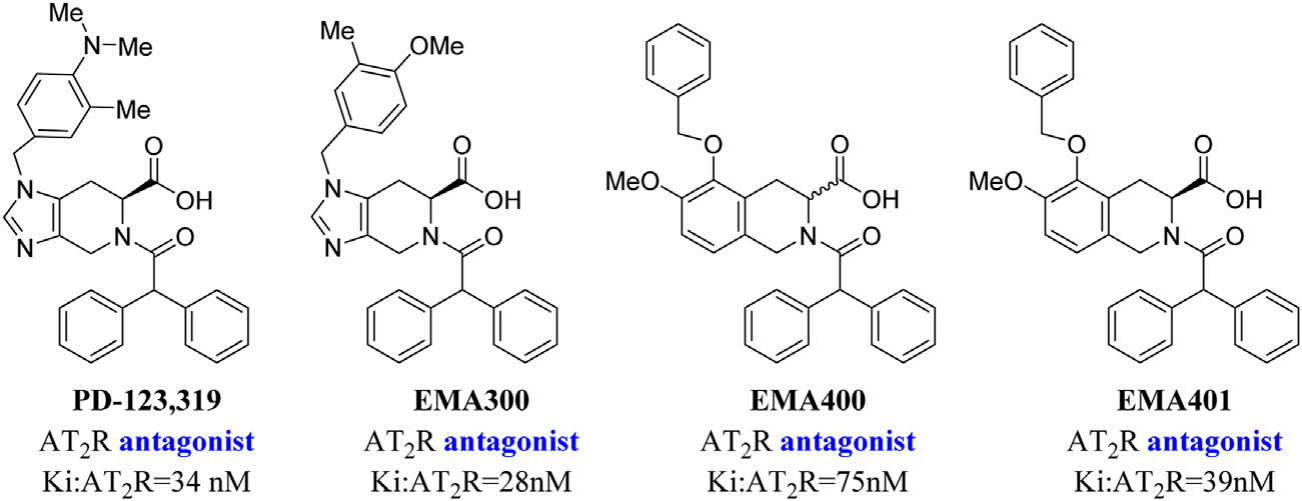
AT_2_ receptor antagonist with the tetrahydroisoquinoline structure.

**PD-123,319** is the classical AT_2_ receptor antagonist. Why then did we design a novel series compound with a benzenesulfonamide structure? As shown in Figure 5, an AT_2_ receptor antagonist with the tetrahydroisoquinoline structure, especially **EMA401**, has been reported to exhibit analgesic efficacy in animal models, and finished the phase II clinical trial for neuropathic pain with satisfactory results. However, more recent trials were discontinued due to drug toxicity after most patients had completed enrollment (Smith et al., 2013; Rice et al., 2014; Smith and Muralidharan, 2015; Smith et al., 2016), which indicated that **EMA401** was ineffective. Therefore, we speculated that the tetrahydroisoquinoline structure may have had some “off-target” effects or pharmacokinetic problems. Consequently, we focused on the benzenesulfonamide scaffold.

**FIGURE 6:**
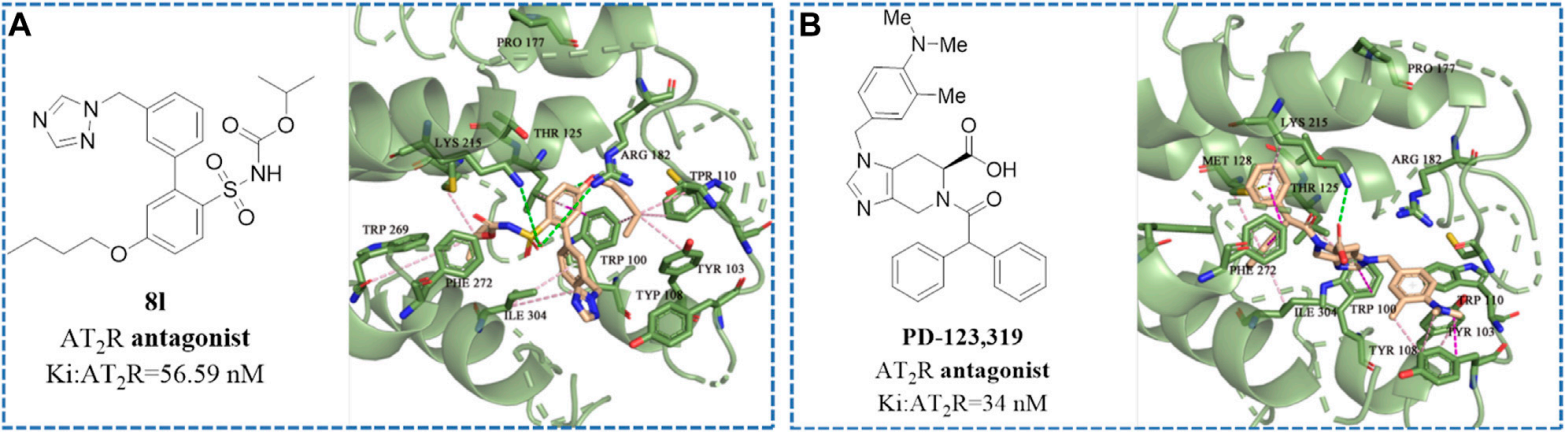
The structural formula and docking results of **(A)** compound **8l** and **(B) PD-123,319** (Blankley et al., 1991) (*Ki*: AT_2_R = 34 nM). The green dotted line indicates hydrogen bond interaction. The purple dotted line indicates hydrophobic interaction.

As shown in Figure 6, both **8l** and **PD-123,319** can bind to the receptor protein through hydrophobic bonds with amino acid residues, such as Thr 125, Lys 215, Met 128, Tyr 103, and Trp 100, and occupy the cavity of the receptor protein. Furthermore, **81** (*Ki*: AT_2_R = 56.59 nM) and **PD-123,319** [*Ki*: AT_2_R = 34 nM (Blankley et al., 1991)] have almost familiar binding affinity to the AT_2_ receptor. Therefore, the formation of these hydrophobic bonds is considered to be an important molecular basis for compounds to maintain high affinity for AT_2_ receptors.

## 4 Conclusion

A series of novel AT_2_ receptor selective compounds with benzenesulfonamide scaffolds have been synthesized and evaluated. Among them, **8j** with agonist activity and compounds **8i**, **8k**, **8d**, **8l**, and **8h** exhibited moderate higher selectivity and antagonist activity. The structure activity relationship and the key binding site of the AT_2_ receptor were discussed. These results may provide potential compounds and references for further development.

## 5 Experimental section

### 5.1 Chemistry

Starting material and solvents were purchased from commercial sources. Reactions were monitored by thin-layer chromatography (TLC) using precoated silica gel plates (silica gel GF/UV 254), and spots were visualized under UV light (254 nm). Melting points (uncorrected) were determined on a Mel-TEMP II melting point apparatus and are uncorrected. ^1^H NMR and ^13^C NMR spectra were recorded with a Bruker Avance 300 MHz spectrometer at 300 K, using TMS as an internal standard. MS spectra or high-resolution mass spectra (HRMS) were recorded on a Shimadzu GC-MS 2050 (ESI) or an Agilent 1946AMSD (ESI) Mass Spectrum. MS spectra or high-resolution mass Column chromatography was performed with silica gel (200e300 mesh). Chemical shifts were reported on the d scale and J values were given in Hz. The synthesis of intermediates **4a-4c** is shown in the Supplementary Material.

#### 5.1.1 General procedure A: Synthesis of intermediates (**5a-5b**)

+To a 250 ml round-bottom flask was added potassium carbonate (4.98 g, 36.01 mmol) dissolved in acetone (100 ml). The base was activated at room temperature by stirring for 10 min. Afterward, 3-bromobenzyl bromide (6 g, 24.01 mmol), 1,2,4-triazole or pyrazole (36.01 mmol), and potassium iodide (797.03 mg, 4.80 mmol) were added to a round-bottom flask and stirred 15 h at 55°C. The reaction was diluted with water and ethyl acetate. The layers were separated, and the organic layer was washed twice with saturated NaCl solution and dried with Na_2_SO_4_. The organic layer was concentrated to give **5a** (Yellow oil) or **5b** (Yellow oil).

#### 5.1.2 General procedure B: Synthesis of intermediates (**6a-6f**)

Compounds **5a** (2.89 g, 12.13 mmol), **4a** (3.80 g, 12.13 mmol), Pd(OAc)_2_ (54.48 mg, 242.64 umol), PPh_3_ (254.57 mg, 970.57 umol), toluene (30 ml), ethanol (20 ml), and NaOH (1.6 M, 15 ml) were added to a 250 ml round-bottom flask and stirred for 5 h at 90°C. The reaction was extracted with ethyl acetate. The organic layers were combined, washed with saturated NaCl solution, dried over MgSO_4_, and concentrated in vacuo to give crude product. The residue was purified on silica gel with PE/EA (5:1) as eluent to get **6a**. Yellow oil (3.20 g, 61.83%).

Compounds **6b-6f** were prepared by the same synthetic method as **6a**.

#### 5.1.3 General procedure C: Synthesis of intermediates (**7a-7f**)

To a 100 ml round-bottom flask was added **6a** (1.80 g, 4.22 mmol). Then, trifluoroacetic acid (6.92 ml, 92.83 mmol) was added slowly to the solution in an ice bath and the mixture was stirred under N_2_ atmosphere for 12 h at room temperature. The reaction mixture was evaporated and most TFA was removed, leaving oil which was dissolved in ethyl acetate and washed with water and saturated NaCl solution. The crude product was achieved after drying over anhydrous Na_2_SO_4_ and evaporating under reduced pressure. Using silica gel column chromatography (PE/EA as eluent), the residue was purified to obtain **7a**. White solid (1.0 g, 63.97%). ^1^H NMR (300 MHz, Chloroform-*d*) δ 8.20 (s, 1H, triazole-**H**), 8.05 (d, *J* = 8.1 Hz, 1H, triazole-**H**), 7.94 (s, 1H, Ar-**H**), 7.50 (d, *J* = 8.3 Hz, 3H, Ar-**H**), 7.34 (d, *J* = 6.4 Hz, 1H, Ar-**H**), 7.30 (d, *J* = 8.2 Hz, 1H, Ar-**H**), 7.13 (d, *J* = 1.4 Hz, 1H, Ar-**H**), 5.41 (s, 2H, -C**H**_**2**_), 4.74 (s, 2H, -N**H**_**2**_), 2.58 (d, *J* = 7.2 Hz, 2H, -C**H**_**2**_), 1.94 (m, 1H, -C**H**), 0.96 (d, *J* = 6.6 Hz, 6H, -C**H**_**3**_). ^13^C NMR (75 MHz, CDCl_3_) δ 152.25, 146.72, 140.18, 139.24, 138.27, 134.43, 132.71, 129.69, 129.52, 129.01, 128.57, 127.84, 53.30, 44.96, 30.07, 22.34. HR-MS: [M + H]^+^: 371.1542 **C**_**19**_ **H**_**22**_ **N**_**4**_ **O**_**2**_ **S** require 371.1542.

Compounds **7b-7f** were prepared by the same synthetic method as **7a**.

#### 5.1.4 General procedure D: Synthesis of target compounds (**8a-8l, 9a-9h**)

N-((3'-((1H-1,2,4-triazol-1-yl)methyl)-5-isobutyl-[1,1′-biphenyl]-2-yl)sulfonyl)pentanamide **(8a**).

Compound **7a** (200 mg, 539.85 umol) was dissolved in DCM (5 ml), followed by triethylamine (375.20 mmL, 2.70 mmol) and valeryl chloride (127.63 mmL, 1.08 mmol) added on an ice bath. The reaction mixture was stirred for 4 h at room temperature under N_2_ atmosphere. The reaction was extracted with ethyl acetate. The organic layers were combined, washed with saturated NaCl solution, dried over MgSO_4_, and concentrated in vacuo. The crude product was purified by silica gel column chromatography using CH_2_Cl_2_/MeOH as an eluent to give **8a**. White solid (146 mg, 59.49%). m.p. 138.4–139.7°C. ^1^H NMR (300 MHz, Chloroform-*d*) δ 9.88 (s, 1H, -N**H**), 8.26 (d, *J* = 8.2 Hz, 1H, Ar-**H**), 8.22 (s, 1H, triazole-**H**), 7.96 (s, 1H, triazole-**H**), 7.63 (s, 1H, Ar-**H**), 7.49-7.27 (m, 4H, Ar-**H**), 7.15 (s, 1H, Ar-**H**), 5.34 (s, 2H, -C**H**_**2**_), 2.59 (d, *J* = 7.0 Hz, 2H, -C**H**_**2**_), 1.97 (t, *J* = 6.9 Hz, 3H, -C**H**_**2**_, -C**H**), 1.46 (m, 2H, -C**H**_**2**_), 1.31-1.20 (m, 2H, -C**H**_**2**_), 0.96 (d, *J* = 6.5 Hz, 6H, -C**H**_**3**_), 0.85 (t, *J* = 7.2 Hz, 3H, -C**H**_**3**_). ^13^C NMR (75 MHz, CDCl_3_) δ 171.15, 147.97, 140.02, 139.50, 134.96, 133.63, 132.71, 130.93, 130.32, 129.59, 128.99, 128.93, 127.79, 53.50, 45.09, 35.46, 30.02, 26.04, 22.39, 22.08, 13.72. ESI-MS: [M + H]^+^: 455.2107 **C**_**24**_ **H**_**31**_ **N**_**4**_ **O**_**3**_ **S** require 455.2111.

((3'-((1H-1,2,4-triazol-1-yl)methyl)-5-isobutyl-[1,1′-biphenyl]-2-yl)sulfonyl)carbamae **(8b**).

Compound **8b** was prepared as described for **8a**, replacing valeryl chloride with butyl chloroformate. Yield: 125.60 mg (49.44%). m.p. 148.1-149.3°C; ^1^H NMR (400 MHz, DMSO-*d*_6_) δ 11.47 (s, 1H, -N**H**), 8.60 (s, 1H, triazole-**H**), 8.00 (s, 1H, triazole-**H**), 7.97 (d, *J* = 8.2 Hz, 1H, Ar-**H**), 7.41 (dd, *J* = 14.5, 7.8 Hz, 2H, Ar-**H**), 7.29 (d, *J* = 7.7 Hz, 1H, Ar-**H**), 7.23 (d, *J* = 7.9 Hz, 2H, Ar-**H**), 7.12-7.09 (m, 1H, Ar-**H**), 5.46 (s, 2H, -C**H**_**2**_), 3.93 (t, *J* = 6.5 Hz, 2H, -C**H**_**2**_), 2.56 (d, *J* = 7.1 Hz, 2H, -C**H**_**2**_), 1.89 (m, 1H, -C**H**), 1.39 (m, 2H, -C**H**_**2**_), 1.15 (m, 2H, -C**H**_**2**_), 0.88 (d, *J* = 6.6 Hz, 6H, -C**H**_**3**_), 0.81 (t, *J* = 7.4 Hz, 3H, -C**H**_**3**_). ^13^C NMR (101 MHz, DMSO) δ 152.11, 151.32, 147.46, 144.78, 140.50, 139.58, 135.93, 135.43, 133.49, 130.17, 128.94, 128.85, 128.66, 128.41, 127.62, 65.88, 52.60, 44.31, 30.49, 29.87, 22.54, 18.73, 13.89. ESI-MS: [M + H]^+^: 471.2055 **C**_**24**_ **H**_**31**_ **N**_**4**_ **O**_**4**_ **S** require 471.2061.

Isobutyl((3'-((1H-1,2,4-triazol-1-yl)methyl)-5-isobutyl-[1,1′-biphenyl]-2-yl)sulfonyl)carbamate **(8c**).

Compound **8c** was prepared as described for **8a**, replacing valeryl chloride with isobutyl chloroformate. Yield: 111.00 mg (45.23%). M.p. 145.3-146.2°C; ^1^H NMR (300 MHz, Chloroform-*d*) δ 9.62 (s, 1H, -N**H**), 8.24 (d, *J* = 8.2 Hz, 1H, triazole-**H**), 8.17 (s, 1H, triazole-**H**), 7.95 (s, 1H, Ar-**H**), 7.56 (s, 1H, Ar-**H**), 7.47-7.28 (m, 4H, Ar-**H**), 7.16 (d, *J* = 1.4 Hz, 1H, Ar-**H**), 5.31 (s, 2H, -C**H**_**2**_), 3.83 (d, *J* = 6.5 Hz, 2H, -C**H**_**2**_), 2.60 (d, *J* = 7.1 Hz, 2H, -C**H**_**2**_), 1.96 (m, 1H, -C**H**), 1.81 (m, 1H, -C**H**), 0.97 (d, *J* = 6.6 Hz, 6H, -C**H**_**3**_), 0.81 (d, *J* = 6.7 Hz, 6H, -C**H**_**3**_). ^13^C NMR (75 MHz, Chloroform-*d*) δ 152.03, 150.85, 147.93, 143.22, 139.99, 139.79, 134.89, 133.64, 132.71, 130.96, 130.03, 129.38, 128.93, 128.70, 127.74, 72.58, 53.43, 45.07, 30.05, 27.61, 22.36, 18.76. ESI-MS: [M + H]^+^: 471.2043 **C**_**24**_ **H**_**31**_ **N**_**4**_ **O**_**4**_ **S** require 471.2061.

Isopropyl((3’-((1H-1,2,4-triazol-1-yl)methyl)-5-isobutyl-[1,1′-biphenyl]-2-yl)sulfonyl)carbamate **(8d**).

`Compound **8d** was prepared as described for **8a**, replacing valeryl chloride with isopropyl chloroformate. Yield: 145.00 mg (57.08%). m.p. 150.9-151.7°C; ^1^H NMR (400 MHz, DMSO-*d*_6_) δ 11.34 (s, 1H, -N**H**), 8.60 (s, 1H, triazole-**H**), 7.98 (d, *J* = 9.2 Hz, 2H, triazole-**H**, Ar-**H**), 7.47-7.37 (m, 2H, Ar-**H**), 7.29 (d, *J* = 7.8 Hz, 1H, Ar-**H**), 7.26∼7.20 (m, 2H, Ar-**H**), 7.12 (d, *J* = 15.9 Hz, 1H, Ar-**H**), 5.46 (s, 2H, -C**H**_**2**_), 4.69 (m, 1H, -C**H**), 2.56 (d, *J* = 7.1 Hz, 2H, -C**H**_**2**_), 1.89 (m, 1H, -C**H**), 1.05 (d, *J* = 6.2 Hz, 6H, -C**H**_**3**_), 0.88 (d, *J* = 6.6 Hz, 6H, -C**H**_**3**_). ^13^C NMR (101 MHz, DMSO-*d*_6_) δ 152.08, 150.74, 147.39, 144.78, 140.47, 139.53, 135.84, 135.55, 133.45, 130.30, 129.00, 128.97, 128.76, 128.65, 127.62, 70.13, 52.64, 44.31, 29.87, 22.50, 21.83. ESI-MS: [M + H]^+^: 457.1886 **C**_**23**_ **H**_**29**_ **N**_**4**_ **O**_**4**_ **S** require 457.1904.

N-((3'-((1H-1,2,4-triazol-1-yl)methyl)-5-butyl-[1,1′-biphenyl]-2-yl)sulfonyl)pentanamide **(8e**).

Yield: 153.00 mg (43.29%). m.p. 141.8–142.4°C; ^1^H NMR (400 MHz, DMSO-*d*_6_) δ 11.44 (s, 1H, -N**H**), 8.63 (s, 1H, triazole-**H**), 8.02-7.97 (m, 2H, triazole-**H**, Ar-**H**), 7.45 (m, 1H, Ar-**H**), 7.40 (d, *J* = 7.6 Hz, 1H, Ar-**H**), 7.28 (m, 3H, Ar-**H**), 7.11 (d, *J* = 1.6 Hz, 1H, Ar-**H**), 5.46 (s, 2H, -C**H**_**2**_), 2.73-2.64 (m, 2H, -C**H**_**2**_), 1.98 (t, *J* = 7.4 Hz, 2H, -C**H**_**2**_), 1.64-1.53 (m, 2H, -C**H**_**2**_), 1.38-1.32 (m, 2H, -C**H**_**2**_), 1.30 (m, 2H, -C**H**_**2**_), 1.17 (m, 2H, -C**H**_**2**_), 0.90 (t, *J* = 7.3 Hz, 3H, -C**H**_**3**_), 0.81 (t, *J* = 7.3 Hz, 3H, -C**H**_**3**_). ^13^C NMR (101 MHz, DMSO) δ 171.74, 152.16, 148.64, 144.78, 140.55, 139.68, 136.01, 135.39, 132.77, 130.64, 129.15, 129.02, 128.61, 128.14, 127.66, 52.57, 35.17, 34.82, 33.01, 26.29, 22.28, 21.96, 14.18, 14.08. ESI-MS: [M + H]^+^: 455.2186 **C**_**24**_ **H**_**31**_ **N**_**4**_ **O**_**3**_ **S** require 455.2111.

Butyl((3'-((1H-1,2,4-triazol-1-yl)methyl)-5-butyl-[1,1′-biphenyl]-2-yl)sulfonyl)carbamate **(8f**).

Yield: 116.00 mg (50.34%). m.p. 150.1-151.4°C; ^1^H NMR (400 MHz, DMSO-*d*_6_) δ 11.46 (s, 1H, -N**H**), 8.60 (s, 1H, triazole-**H**), 8.03∼7.99 (m, 1H, triazole-**H**), 7.97 (dd, *J* = 8.3, 1.6 Hz, 1H, Ar-**H**), 7.46 (d, *J* = 8.3 Hz, 1H, Ar-**H**), 7.41 (t, *J* = 7.5 Hz, 1H, Ar-**H**), 7.29 (d, *J* = 7.7 Hz, 1H, Ar-**H**), 7.23 (d, *J* = 7.6 Hz, 2H, Ar-**H**), 7.14 (s, 1H, Ar-**H**), 5.46 (s, 2H, -C**H**_**2**_), 3.94 (t, *J* = 6.4 Hz, 2H, -C**H**_**2**_), 2.68 (t, *J* = 7.6 Hz, 2H, -C**H**_**2**_), 1.59 (m, 2H, -C**H**_**2**_), 1.45-1.37 (m, 2H, -C**H**_**2**_), 1.36-1.28 (m, 2H, -C**H**_**2**_), 1.16 (m, 2H, -C**H**_**2**_), 0.93∼0.87 (m, 3H, C**H**_**3**_), 0.86∼0.76 (m, 3H, -C**H**_**3**_). ^13^C NMR (101 MHz, DMSO) δ 152.10, 151.36, 148.65, 144.77, 140.65, 139.60, 135.88, 135.39, 132.84, 130.32, 128.95, 128.93, 128.63, 128.17, 127.61, 65.85, 52.62, 34.80, 33.03, 30.50, 22.25, 18.73, 14.18, 13.89. ESI-MS: [M + H]^+^: 472.2199 **C**_**24**_ **H**_**31**_ **N**_**4**_ **O**_**4**_ **S** require 471.2061.

Isobutyl((3'-((1H-1,2,4-triazol-1-yl)methyl)-5-butyl-[1,1′-biphenyl]-2-yl)sulfonyl)carbamate **(8g**).

Yield: 119.00 mg (55.79%). m.p. 168.4–169.1°C; ^1^H NMR (300 MHz, Chloroform-*d*) δ 8.24 (d, *J* = 8.2 Hz, 1H, Ar-**H**), 8.16 (s, 1H, triazole-**H**), 7.95 (s, 1H, -N**H**), 7.54 (s, 1H, triazole-**H**), 7.40 (d, *J* = 7.4 Hz, 2H, Ar-**H**), 7.33 (d, *J* = 13.0 Hz, 2H, Ar-**H**), 7.24 (d, *J* = 7.5 Hz, 1H, Ar-**H**), 7.18 (d, *J* = 1.6 Hz, 1H, Ar-**H**), 5.30 (s, 2H, -C**H**_**2**_), 3.83 (d, *J* = 6.5 Hz, 2H, -C**H**_**2**_), 2.81-2.66 (m, 2H, -C**H**_**2**_), 1.82 (m, 1H, -C**H**), 1.74-1.60 (m, 2H, -C**H**_**2**_), 1.42 (m, 2H, -C**H**_**2**_), 0.98 (t, *J* = 7.3 Hz, 3H, -C**H**_**3**_), 0.81 (d, *J* = 6.7 Hz, 6H, -C**H**_**3**_). ^13^C NMR (75 MHz, Chloroform-*d*) δ 152.05, 150.86, 149.12, 143.23, 140.14, 139.81, 134.77, 133.66, 132.08, 131.09, 129.97, 129.37, 128.89, 128.02, 127.69, 72.57, 53.41, 35.44, 33.06, 27.61, 22.38, 18.74, 13.89. ESI-MS: [M + H]^+^: 471.2061 **C**_**24**_ **H**_**31**_ **N**_**4**_ **O**_**4**_ **S** require 471.2061.

Isopropyl((3'-((1H-1,2,4-triazol-1-yl)methyl)-5-butyl-[1,1′-biphenyl]-2-yl)sulfonyl)carbamate (**8h**).

Yield: 149.00 mg (59.51%). m.p. 167.7–168.3°C; ^1^H NMR (300 MHz, Chloroform-*d*) δ 8.23 (d, *J* = 8.2 Hz, 1H, Ar-**H**), 8.14 (s, 1H, triazole-**H**), 7.93 (s, 1H, -N**H**), 7.51 (s, 1H, triazole-**H**), 7.40 (dd, *J* = 7.8, 2.6 Hz, 2H, Ar-**H**), 7.35 (s, 1H, Ar-**H**), 7.32 (d, *J* = 4.7 Hz, 1H, Ar-**H**), 7.23 (d, *J* = 7.4 Hz, 1H, Ar-**H**), 7.17 (d, *J* = 1.5 Hz, 1H, Ar-**H**), 5.31 (s, 2H, -C**H**_**2**_), 4.86 (m, 1H, -C**H**), 2.81-2.69 (m, 2H, -C**H**_**2**_), 1.67 (m, 2H, -C**H**_**2**_), 1.48-1.36 (m, 2H, -C**H**_**2**_), 1.15 (d, *J* = 6.3 Hz, 6H, -C**H**_**3**_), 0.97 (t, *J* = 7.3 Hz, 3H, -C**H**_**3**_). ^13^C NMR (75 MHz, Chloroform-*d*) δ 152.04, 150.28, 149.07, 143.23, 140.16, 139.78, 134.82, 133.71, 132.08, 131.19, 129.89, 129.43, 128.81, 127.92, 127.64, 70.74, 53.38, 35.44, 33.06, 22.37, 21.66, 13.89. ESI-MS: [M + H]^+^: 457.1910 **C**_**23**_ **H**_**29**_ **N**_**4**_ **O**_**4**_ **S** require 457.1904.

N-((3'-((1H-1,2,4-triazol-1-yl)methyl)-5-butoxy-[1,1′-biphenyl]-2-yl)sulfonyl)pentanamide (**8i**).

Yield: 124.00 mg (50.94%). m.p. 167.4–168.8°C; ^1^H NMR (300 MHz, Chloroform-*d*) δ 9.72 (s, 1H, -N**H**), 8.30 (d, *J* = 9.0 Hz, 1H, triazole-**H**), 8.21 (s, 1H, triazole-**H**), 7.96 (s, 1H, Ar-**H**), 7.61 (s, 1H, Ar-**H**), 7.43 (t, *J* = 7.6 Hz, 1H, Ar-**H**), 7.34 (d, *J* = 7.7 Hz, 1H, Ar-**H**), 7.30 (d, *J* = 4.3 Hz, 1H, Ar-**H**), 7.05 (dd, *J* = 9.0, 2.6 Hz, 1H, Ar-**H**), 6.84 (d, *J* = 2.6 Hz, 1H, Ar-**H**), 5.34 (s, 2H, -C**H**_**2**_), 4.07 (t, *J* = 6.4 Hz, 2H, -C**H**_**2**_), 2.01 (t, *J* = 7.4 Hz, 2H, -C**H**_**2**_), 1.83 (m, 2H, -C**H**_**2**_), 1.52 (m, 4H, -C**H**_**2**_), 1.28 (m, 2H, -C**H**_**2**_), 1.02 (t, *J* = 7.4 Hz, 3H, -C**H**_**3**_), 0.86 (t, *J* = 7.3 Hz, 3H, -C**H**_**3**_). ^13^C NMR (75 MHz, Chloroform-*d*) δ 171.08, 162.61, 152.18, 143.48, 141.91, 139.82, 133.63, 129.43, 128.98, 128.94, 127.92, 118.22, 113.06, 93.76, 68.30, 53.50, 35.51, 31.03, 26.07, 22.11, 19.16, 13.80, 13.74. ESI-MS: [M + H]^+^: 471.2044 **C**_**24**_ **H**_**31**_ **N**_**4**_ **O**_**4**_ **S** require 471.2061.

Butyl((3'-((1H-1,2,4-triazol-1-yl)methyl)-5-butoxy-[1,1′-biphenyl]-2-yl)sulfonyl)carbamate (**8j**).

Yield: 118.00 mg (51.63%). m.p. 158.9–160.4°C; ^1^H NMR (300 MHz, Chloroform-*d*) δ 9.46 (s, 1H, -N**H**), 8.26 (d, *J* = 8.9 Hz, 1H, triazole-**H**), 8.15 (s, 1H, triazole-**H**), 7.94 (s, 1H, Ar-**H**), 7.51 (s, 1H, Ar-**H**), 7.41 (t, *J* = 7.5 Hz, 1H, Ar-**H**), 7.34 (m, 1H, Ar-**H**), 7.31 (s, 1H, Ar-**H**), 7.04 (dd, *J* = 9.0, 2.6 Hz, 1H, Ar-**H**), 6.85 (d, *J* = 2.6 Hz, 1H, Ar-**H**), 5.31 (s, 2H, -C**H**_**2**_), 4.07 (td, *J* = 6.5, 2.6 Hz, 4H, -C**H**_**2**_), 1.89-1.77 (m, 2H, -C**H**_**2**_), 1.53 (m, 4H, -C**H**_**2**_), 1.26 (dd, *J* = 15.3, 7.6 Hz, 2H, -C**H**_**2**_), 1.02 (t, *J* = 7.4 Hz, 3H, -C**H**_**3**_), 0.90 (t, *J* = 7.3 Hz, 3H, -C**H**_**3**_). ^13^C NMR (75 MHz, Chloroform-*d*) δ 162.58, 152.04, 151.50, 150.92, 143.23, 142.43, 139.62, 133.67, 129.71, 129.23, 128.81, 127.80, 121.66, 118.11, 112.95, 68.32, 66.40, 53.39, 31.04, 30.49, 19.16, 18.81, 13.80, 13.61. ESI-MS: [M + H]^+^: 487.1998 **C**_**24**_ **H**_**31**_ **N**_**4**_ **O**_**5**_ **S** require 487.2010.

Isobutyl((3'-((1H-1,2,4-triazol-1-yl)methyl)-5-butoxy-[1,1′-biphenyl]-2-yl)sulfonyl)carbamate (**8k**).

Yield: 109.00 mg (42.38%). m.p. 172.3–173.1°C; ^1^H NMR (300 MHz, Chloroform-*d*) δ 9.48 (s, 1H, -N**H**), 8.27 (d, *J* = 8.9 Hz, 1H, triazole-**H**), 8.15 (s, 1H, triazole-**H**), 7.95 (s, 1H, Ar-**H**), 7.53 (s, 1H, Ar-**H**), 7.41 (t, *J* = 7.5 Hz, 1H, Ar-**H**), 7.34 (d, *J* = 7.7 Hz, 1H, Ar-**H**), 7.31 (s, 1H, Ar-**H**), 7.04 (dd, *J* = 9.0, 2.6 Hz, 1H, Ar-**H**), 6.85 (d, *J* = 2.5 Hz, 1H, Ar-**H**), 5.30 (s, 2H, -C**H**_**2**_), 4.07 (t, *J* = 6.4 Hz, 2H, -C**H**_**2**_), 3.84 (d, *J* = 6.6 Hz, 2H, -C**H**_**2**_), 1.88-1.77 (m, 3H, -C**H**_**2**_, -C**H**), 1.53 (m, 2H, -C**H**_**2**_), 1.02 (t, *J* = 7.4 Hz, 3H, -C**H**_**3**_), 0.84 (d, *J* = 6.7 Hz, 6H, -C**H**_**3**_). ^13^C NMR (75 MHz, Chloroform-*d*) δ 162.58, 152.13, 150.93, 143.22, 142.40, 139.63, 133.66, 133.62, 129.81, 129.23, 128.90, 128.84, 127.82, 118.13, 112.97, 72.51, 68.32, 53.39, 31.02, 27.64, 19.15, 18.78, 13.79. ESI-MS: [M + H]^+^: 487.1995 **C**_**24**_ **H**_**31**_ **N**_**4**_ **O**_**5**_ **S** require 487.2010.

Isopropyl((3'-((1H-1,2,4-triazol-1-yl)methyl)-5-butoxy-[1,1′-biphenyl]-2-yl)sulfonyl)carbamate (**8l**).

Yield: 163.00 mg (61.82%). m.p. 166.7-168.1°C; ^1^H NMR (300 MHz, Chloroform-*d*) δ 9.26 (s, 1H, -N**H**), 8.25 (d, *J* = 8.9 Hz, 1H, triazole-**H**), 8.14 (s, 1H, triazole-**H**), 7.94 (s, 1H, Ar-**H**), 7.50 (s, 1H, Ar-**H**), 7.39 (d, *J* = 7.5 Hz, 1H, Ar-**H**), 7.33 (d, *J* = 13.1 Hz, 2H, Ar-**H**), 7.03 (dd, *J* = 9.0, 2.5 Hz, 1H, Ar-**H**), 6.84 (d, *J* = 2.5 Hz, 1H, Ar-**H**), 5.30 (s, 2H, -C**H**_**2**_), 4.87 (m, 1H, -C**H**), 4.07 (t, *J* = 6.4 Hz, 2H, -C**H**_**2**_), 1.82 (m, 2H, -C**H**_**2**_), 1.53 (m, 2H, -C**H**_**2**_), 1.18 (d, *J* = 6.2 Hz, 6H, -C**H**_**3**_), 1.01 (t, *J* = 7.4 Hz, 3H, -C**H**_**3**_). ^13^C NMR (75 MHz, Chloroform-*d*) δ 162.53, 152.04, 150.32, 143.20, 142.38, 139.57, 133.68, 129.72, 129.26, 128.88, 128.83, 127.79, 118.09, 116.27, 112.91, 70.70, 68.31, 53.39, 31.03, 21.72, 19.15, 13.80. ESI-MS: [M + H]^+^: 473.1846 **C**_**23**_ **H**_**29**_ **N**_**4**_ **O**_**5**_ **S** require 473.1853.

N-((3'-((1H-pyrazol-1-yl)methyl)-5-isobutyl-[1,1′-biphenyl]-2-yl)sulfonyl)pentanamide (**9a**).

Yield: 121.00 mg (47.96%). m.p. 125.8-126.7°C; ^1^H NMR (400 MHz, DMSO-*d*_6_) δ 11.46 (s, 1H, -N**H**), 8.00 (d, *J* = 8.2 Hz, 1H, Ar-**H**), 7.82 (d, *J* = 2.1 Hz, 1H, pyrazole-**H**), 7.48 (d, *J* = 1.5 Hz, 1H, Ar-**H**), 7.40 (m, 2H, Ar-**H**), 7.26-7.17 (m, 3H, Ar-**H,** pyrazole-**H**), 7.07 (d, *J* = 1.6 Hz, 1H, Ar-**H**), 6.28 (t, *J* = 2.0 Hz, 1H, pyrazole-**H**), 5.39 (s, 2H, -C**H**_**2**_), 2.55 (d, *J* = 7.1 Hz, 2H, -C**H**_**2**_), 1.99 (t, *J* = 7.4 Hz, 2H, -C**H**_**2**_), 1.88 (m, 1H, -C**H**), 1.39-1.29 (m, 2H, -C**H**_**2**_), 1.17 (m, 2H, -C**H**_**2**_), 0.88 (d, *J* = 6.6 Hz, 6H, -C**H**_**3**_), 0.81 (t, *J* = 7.3 Hz, 3H, -C**H**_**3**_). ^13^C NMR (101 MHz, DMSO-*d*_6_) δ 171.71, 147.38, 140.54, 139.51, 139.43, 137.50, 135.46, 133.37, 130.70, 130.52, 128.75, 128.72, 128.67, 128.48, 127.25, 105.93, 55.08, 44.34, 35.17, 29.84, 26.29, 22.55, 21.95, 14.06. ESI-MS: [M + H]^+^: 454.2142 **C**_**25**_ **H**_**31**_ **N**_**3**_ **O**_**3**_ **S** require 454.2159.

Butyl((3'-((1H-pyrazol-1-yl)methyl)-5-isobutyl-[1,1′-biphenyl]-2-yl)sulfonyl)carbamate (**9b**).

Yield: 120.00 mg (48.39%). m.p. 129.1-131.4°C; ^1^H NMR (400 MHz, DMSO-*d*_6_) δ 11.47 (s, 1H, -N**H**), 7.97 (d, *J* = 8.2 Hz, 1H, Ar-**H**), 7.80 (d, *J* = 1.8 Hz, 1H, pyrazole-**H**), 7.47 (d, *J* = 1.6 Hz, 1H, Ar-**H**), 7.42 (m, 1H, Ar-**H**), 7.37 (t, *J* = 7.8 Hz, 1H, Ar-**H**), 7.22-7.16 (m, 3H, Ar-**H,** pyrazole-**H**), 7.09 (d, *J* = 1.6 Hz, 1H, Ar-**H**), 6.27 (t, *J* = 2.0 Hz, 1H, pyrazole-**H**), 5.38 (s, 2H, -C**H**_**2**_), 3.93 (t, *J* = 6.5 Hz, 2H, -C**H**_**2**_), 2.56 (d, *J* = 7.1 Hz, 2H, -C**H**_**2**_), 1.89 (m, 1H, -C**H**_**2**_), 1.39 (m, 2H, -C**H**_**2**_), 1.15 (m, 2H, -C**H**_**2**_), 0.88 (d, *J* = 6.6 Hz, 6H, -C**H**_**3**_), 0.81 (t, *J* = 7.4 Hz, 3H, -C**H**_**3**_). ^13^C NMR (101 MHz, DMSO-*d*_6_) δ 151.35, 147.37, 140.67, 139.45, 139.35, 137.44, 135.50, 133.47, 130.71, 130.64, 130.21, 128.74, 128.56, 128.46, 127.17, 105.91, 65.85, 55.12, 44.34, 30.52, 29.86, 22.52, 18.74, 13.87. ESI-MS: [M + H]^+^: 470.2107 **C**_**25**_ **H**_**31**_ **N**_**3**_ **O**_**4**_ **S** require 470.2108.

Isobutyl((3'-((1H-pyrazol-1-yl)methyl)-5-isobutyl-[1,1′-biphenyl]-2-yl)sulfonyl)carbamate (**9c**).

Yield: 114.00 mg (45.27%). m.p. 149.6-151.3°C; ^1^H NMR (400 MHz, DMSO-*d*_6_) δ 11.47 (s, 1H, -N**H**), 7.98 (d, *J* = 8.2 Hz, 1H, Ar-**H**), 7.82-7.77 (m, 1H, pyrazole-**H**), 7.51-7.45 (m, 1H, Ar-**H**), 7.44-7.36 (m, 2H, Ar-**H**), 7.23-7.17 (m, 3H, pyrazole-H, Ar-**H**), 7.09 (s, 1H, Ar-**H**), 6.27 (t, *J* = 2.1 Hz, 1H, pyrazole-**H**), 5.38 (s, 2H, -C**H**_**2**_), 3.73 (d, *J* = 6.4 Hz, 2H, -C**H**_**2**_), 2.55 (d, *J* = 7.1 Hz, 2H, -C**H**_**2**_), 1.88 (m, 1H, -C**H**), 1.70 (m, 1H, -C**H**), 0.88 (d, *J* = 6.5 Hz, 6H, -C**H**_**3**_), 0.73 (d, *J* = 6.7 Hz, 6H, -C**H**_**3**_). ^13^C NMR (101 MHz, DMSO-*d*_6_) δ 151.37, 147.35, 140.63, 139.46, 139.36, 137.45, 135.59, 133.50, 130.72, 130.07, 128.79, 128.56, 128.53, 128.49, 127.16, 105.92, 71.92, 55.11, 44.31, 29.86, 27.65, 22.50, 18.96. ESI-MS: [M + H]^+^: 470.2104 **C**_**25**_ **H**_**31**_ **N**_**3**_ **O**_**4**_ **S** require 470.2108.

Isopropyl((3'-((1H-pyrazol-1-yl)methyl)-5-isobutyl-[1,1′-biphenyl]-2-yl)sulfonyl)carbamate (**9d**).

Yield: 132.00 mg (51.27%). m.p. 146.6-147.5°C; ^1^H NMR (400 MHz, DMSO-*d*_6_) δ 11.35 (s, 1H, -N**H**), 7.98 (d, *J* = 8.2 Hz, 1H, Ar-**H**), 7.80 (d, *J* = 2.1 Hz, 1H, pyrazole-**H**), 7.47 (d, *J* = 1.4 Hz, 1H, Ar-**H**), 7.42 (dd, *J* = 8.3, 1.4 Hz, 1H, Ar-**H**), 7.37 (t, *J* = 7.8 Hz, 1H, Ar-**H**), 7.23-7.16 (m, 3H, pyrazole-**H**, Ar-**H**), 7.09 (d, *J* = 1.5 Hz, 1H, Ar-**H**), 6.27 (t, *J* = 2.0 Hz, 1H, pyrazole-**H**), 5.38 (s, 2H, -C**H**_**2**_), 4.70 (t, *J* = 6.2 Hz, 1H, -C**H**), 2.56 (d, *J* = 7.1 Hz, 2H, -C**H**_**2**_), 1.88 (m, 1H, -C**H**), 1.06 (d, *J* = 6.2 Hz, 6H, -C**H**_**3**_), 0.88 (d, *J* = 6.6 Hz, 6H, -C**H**_**3**_). ^13^C NMR (101 MHz, DMSO-*d*_6_) δ 150.74, 147.35, 140.63, 139.39, 139.36, 137.42, 135.55, 133.46, 130.73, 130.32, 128.70, 128.60, 128.57, 128.48, 127.18, 105.93, 70.12, 55.13, 44.32, 29.87, 22.50, 21.85. ESI-MS: [M + H]^+^: 456.1945 **C**_**24**_ **H**_**29**_ **N**_**3**_ **O**_**4**_ **S** require 456.1952.

N- ((3'-((1H-pyrazol-1-yl)methyl)-5-butyl-[1,1′-biphenyl]-2-yl)sulfonyl)pentanamide **(9e**).

Yield: 149.00 mg (56.19%). m.p. 140.1-141.3°C; ^1^H NMR (300 MHz, DMSO-*d*_6_) δ 11.53 (s, 1H, -N**H**), 8.02 (d, *J* = 8.2 Hz, 1H, Ar-**H**), 7.88-7.84 (m, 1H, pyrazole-**H**), 7.53-7.49 (m, 1H, Ar-**H**), 7.47 (dd, *J* = 8.3, 1.4 Hz, 1H, Ar-**H**), 7.41 (t, *J* = 7.9 Hz, 1H, Ar-**H**), 7.29-7.20 (m, 3H, pyrazole-**H**, Ar-**H**), 7.15-7.10 (m, 1H, Ar-**H**), 6.30 (t, *J* = 2.0 Hz, 1H, pyrazole-**H**), 5.42 (s, 2H, -C**H**_**2**_), 2.70 (t, *J* = 7.7 Hz, 2H, -C**H**_**2**_), 2.02 (t, *J* = 7.3 Hz, 2H, -C**H**_**2**_), 1.60 (m, 2H, -C**H**_**2**_), 1.37 (m, 2H, -C**H**_**2**_), 1.32 (dd, *J* = 7.2, 3.3 Hz, 2H, -C**H**_**2**_), 1.18 (m, 2H, -C**H**_**2**_), 0.92 (t, *J* = 7.3 Hz, 3H, -C**H**_**3**_), 0.83 (t, *J* = 7.2 Hz, 3H, -C**H**_**3**_). ^13^C NMR (75 MHz, DMSO-*d*_6_) δ 171.78, 148.63, 140.67, 139.51, 139.46, 137.53, 135.37, 133.46, 132.76, 130.74, 128.74, 128.66, 128.50, 128.10, 127.27, 105.95, 55.06, 35.14, 34.81, 33.03, 26.27, 22.30, 21.98, 14.21, 14.11. ESI-MS: [M + H]^+^: 454.2144 **C**_**25**_ **H**_**31**_ **N**_**3**_ **O**_**3**_ **S** require 454.2159.

Butyl((3'-((1H-pyrazol-1-yl)methyl)-5-butyl-[1,1′-biphenyl]-2-yl)sulfonyl)carbamate (**9f**).

Yield: 134.00 mg (50.75%). m.p. 142.5-143.1°C; ^1^H NMR (300 MHz, DMSO-*d*_6_) δ 11.54 (s, 1H, -N**H**), 7.99 (d, *J* = 8.2 Hz, 1H, Ar-**H**), 7.83 (d, *J* = 2.1 Hz, 1H, pyrazole-**H**), 7.53-7.44 (m, 2H, Ar-**H**), 7.43-7.35 (m, 1H, Ar-**H**), 7.22 (d, *J* = 8.4 Hz, 3H, pyrazole-**H**, Ar-**H**), 7.14 (d, *J* = 1.3 Hz, 1H, Ar-**H**), 6.30 (t, *J* = 2.0 Hz, 1H, pyrazole-**H**), 5.40 (s, 2H, -C**H**_**2**_), 3.95 (t, *J* = 6.5 Hz, 2H, -C**H**_**2**_), 2.70 (t, *J* = 7.6 Hz, 2H, -C**H**_**2**_), 1.60 (m, 2H, -C**H**_**2**_), 1.43 (dd, *J* = 14.1, 7.4 Hz, 2H, -C**H**_**2**_), 1.37-1.30 (m, 2H, -C**H**_**2**_), 1.17 (m, 2H, -C**H**_**2**_), 0.92 (t, *J* = 7.3 Hz, 3H, -C**H**_**3**_), 0.83 (t, *J* = 7.3 Hz, 3H, -C**H**_**3**_). ^13^C NMR (75 MHz, DMSO-*d*_6_) δ 151.59, 148.58, 147.22, 140.78, 139.48, 139.39, 137.45, 135.46, 132.85, 130.76, 130.37, 130.14, 128.92, 128.55, 127.17, 105.94, 65.81, 55.09, 34.80, 33.07, 30.52, 22.26, 18.75, 14.21, 13.93. ESI-MS: [M + H]^+^: 470.2103 **C**_**25**_ **H**_**31**_ **N**_**3**_ **O**_**4**_ **S** require 470.2108.

Isobutyl((3'-((1H-pyrazol-1-yl)methyl)-5-butyl-[1,1′-biphenyl]-2-yl)sulfonyl)carbamate (**9g**).`

Yield: 107.00 mg (39.27%). m.p. 149.3-151.2°C; ^1^H NMR(400 MHz, DMSO-*d*_6_) δ 11.47 (s, 1H, -N**H**), 7.97 (d, *J* = 8.2 Hz, 1H, Ar-**H**), 7.84-7.76 (m, 1H, pyrazole-**H**), 7.45 (d, *J* = 11.4 Hz, 2H, Ar-**H**), 7.41-7.31 (m, 1H, Ar-**H**), 7.20 (d, *J* = 6.9 Hz, 3H, Ar-**H**, pyrazole-**H**), 7.11 (d, *J* = 9.5 Hz, 1H, Ar-**H**), 6.27 (s, 1H, pyrazole-**H**), 5.38 (s, 2H, -C**H**_**2**_), 3.73 (d, *J* = 6.4 Hz, 2H, C**H**_**2**_), 2.67 (t, *J* = 7.5 Hz, 2H, -C**H**_**2**_), 1.71 (m, 1H, -C**H**), 1.58 (m, 2H, -C**H**_**2**_), 1.33 (dd, *J* = 14.6, 7.2 Hz, 2H, -C**H**_**2**_), 0.89 (t, *J* = 7.3 Hz, 3H, -C**H**_**3**_), 0.74 (d, *J* = 6.7 Hz, 6H, -C**H**_**3**_). ^13^C NMR (101 MHz, DMSO-*d*_6_) δ 151.35, 148.62, 140.80, 139.45, 139.36, 137.42, 135.44, 132.87, 130.72, 130.26, 128.57, 128.51, 128.49, 128.15, 127.18, 105.92, 71.93, 55.11, 34.78, 33.02, 27.65, 22.19, 18.94, 14.17. ESI-MS: [M + H]^+^: 470.2093 **C**_**25**_ **H**_**31**_ **N**_**3**_ **O**_**4**_ **S** require 470.2108.

Isopropyl((3'-((1H-pyrazol-1-yl)methyl)-5-butyl-[1,1′-biphenyl]-2-yl)sulfonyl)carbamate (**9h**).

Yield: 114.00 mg (45.19%). m.p. 169.8-171.3°C; ^1^H NMR(400 MHz, DMSO-*d*_6_) δ 11.36 (s, 1H, -N**H**), 7.97 (d, *J* = 8.2 Hz, 1H, Ar-**H**), 7.80 (d, *J* = 2.1 Hz, 1H, pyrazole-**H**), 7.49-7.43 (m, 2H, Ar-**H**), 7.40-7.34 (m, 1H, Ar-**H**), 7.23-7.16 (m, 3H, Ar-**H,** pyrazole-**H**), 7.12 (d, *J* = 1.6 Hz, 1H, Ar-**H**), 6.27 (t, *J* = 2.0 Hz, 1H, pyrazole-**H**), 5.38 (s, 2H, -C**H**_**2**_), 4.70 (m, 1H, -C**H**), 2.73-2.64 (m, 2H, -C**H**_**2**_), 1.58 (m, 2H, -C**H**_**2**_), 1.37-1.29 (m, 2H, -C**H**_**2**_), 1.06 (d, *J* = 6.3 Hz, 6H, -C**H**_**3**_), 0.89 (t, *J* = 7.3 Hz, 3H, -C**H**_**3**_). ^13^C NMR (101 MHz, DMSO-*d*_6_) δ 150.77, 148.60, 140.78, 139.39, 139.36, 137.39, 135.45, 132.83, 130.72, 130.46, 128.62, 128.56, 128.48, 128.06, 127.19, 105.93, 70.11, 55.13, 34.78, 33.02, 22.19, 21.86, 14.16. ESI-MS: [M + H]^+^: 456.1937 **C**_**24**_ **H**_**29**_ **N**_**3**_ **O**_**4**_ **S** require 456.1952.

### 5.2 Radioligand binding assay

Rat liver membranes were prepared according to the method of Dudley *et al* (Dudley et al., 1990). After HEK-293 cells were transfected with the AT_2_ receptor, a lysis buffer was used to separate the cell membrane of HEK-293 cells (Grieger et al., 2016), using 27-G Resuspend. The lysis solution was mixed and centrifuged at 12,000 g for 10 min at 4°C. Details are shown in the Supplementary Material.

### 5.3 *In vitro* morphological effects studies

NG108-15 cells (China Center for Type Culture Collection CCTCC) were used to study the *in vitro* morphological effects. In their undifferentiated state, neuroblastoma × glioma hybrid NG108-15 cells havea rounded shape and divide actively. The cells were cultured from passage 18–25 in Dulbecco’s modified Eagle’s medium (DMEM, Gibco BRL, Thermo Fisher Technology (China) Co., Ltd, China) with 10% fetal bovine serum (FBS, Gibco), HAT supplement and 50 mg L^−1^ gentamycin at 37°C in 75 cm^2^ Nunclon Delta flasks in a humidified atmosphere of 95% air and 5% CO_2_, as previously described (Buisson et al., 1992). Subcultures were performed at subconfluency. Under these conditions, cells express mainly the AT_2_ receptor subtype (Whitebread et al., 1991). Cells were treated during 3 days, once a day (first treatment 24 h after plating), and micrographs were taken on the fourth day. The details of experimental grouping and administration method are shown in the Supplementary Material.

### 5.4 Molecular docking simulation

The AT_2_ receptor protein (**PDB ID: 5UNF**) was obtained from the **RSCB** protein bank database, the water molecules of theprotein were removed, the endogenous ligands were extracted, the C and D chains were selected, and a series of treatments such as energy optimization, hydrogenation, and side chain repair were carried out on the protein. Using **SYBYL-2.1.1**, the 2D structure of docking ligand is drawn by software and the energy is optimized. Using the docking suite module in **SYBYL**, the active pocket is automatically generated based on the endogenous ligand. The processed protein and ligand molecules are semi-flexibly docked in **SFXC** mode, and the endogenous ligand **8 ES** is used as the template molecule to evaluate the similarity between the docked ligand and the endogenous ligand. Combined with the scoring functions, such as Crash, Ploar, and Similarity, the docking results are comprehensively analyzed to obtain the total score and the conformation of the compound with the highest score is taken as the docking result. Finally, the analysis results module in **SYBYL** is used to judge the action mode of compounds and receptors, and the sequence viewer module is used to determine the key amino acid residues.

### 5.5 Statistical analysis

Biological results are reported as means ± SE. Statistical analysis was performed by using one-way analysis of variance. *p* value of less than 0.05 was considered to be statistically significant.

## Data Availability

The original contributions presented in the study are included in the article/Supplementary Material; further inquiries can be directed to the corresponding author.

## References

[B1] Blankley C. J. Hodges J. C. Klutchko S. R. Himmelsbach R. J. Chucholowski A. Connolly C. J. (1991). Synthesis and structure-activity relationships of a novel series of non-peptide angiotensin II receptor binding inhibitors specific for the AT2 subtype. J. Med. Chem. 34 (11), 3248–3260. 10.1021/jm00115a014 1956044

[B2] Buisson B. Bottari S. P. Gasparo M. D. Gallo-PayetN. Payet M. D. (1992). The angiotensin AT2 receptor modulates T-type calcium current in non-differentiated NG108-15 cells. FEBS Lett. 309 (2), 161–164. 10.1016/0014-5793(92)81086-2 1324194

[B3] Carey Robert M. (2005). Update on the role of the AT2 receptor. Curr. Opin. Nephrol. Hypertens. 14, 67–71. 10.1097/00041552-200501000-00011 15586018

[B4] Connolly A. Holleran B. J. Simard E. Baillargeon J. P. Lavigne P. Leduc R. (2019). Interplay between intracellular loop 1 and helix VIII of the angiotensin II type 2 receptor controls its activation. Biochem. Pharmacol. 168, 330–338. 10.1016/j.bcp.2019.07.018 31348898

[B5] Dudley D. T. Panek R. L. Major T. C. Lu G. H. Bruns R. F. Klinkefus B. A. (1990). Subclasses of angiotensin II binding sites and their functional significance. Mol. Pharmacol. 38 (3), 370–377. 2402226

[B6] Gasparo D. M. (1996). Angiotensin II induction of neurite outgrowth by AT2 receptors in NG108-15 cells. effect counteracted by the AT1 receptors [J]. J. Biol. Chem. 271 (37), 22729. 8798447 10.1074/jbc.271.37.22729

[B7] Grieger J. C. Soltys S. M. Samulski R. J. (2016). Production of recombinant adeno-associated virus vectors using suspension HEK293 cells and continuous harvest of vector from the culture media for GMP FIX and FLT1 clinical vector. Mol. Ther. 24 (2), 287–297. 10.1038/mt.2015.187 26437810 PMC4817810

[B8] Hein L. Barsh G. S. Pratt R. E. Dzau V. J. Kobilka B. K. (1995). Behavioural and cardiovascular effects of disrupting the angiotensin II type-2 receptor gene in mice. Nature 377, 744–747. 10.1038/377744a0 7477266

[B9] Juillerat-Jeanneret L. (2020). The other angiotensin II receptor: AT_2_R as a therapeutic target. J. Med. Chem. 63 (5), 1978–1995. 10.1021/acs.jmedchem.9b01780 32030982

[B10] Liu J. Liu Q. Yang X. Xu S. Zhang H. Bai R. (2013). Design, synthesis, and biological evaluation of 1, 2, 4-triazole bearing 5-substituted biphenyl-2-sulfonamide derivatives as potential antihypertensive candidates. Bioorg. Med. Chem. 21, 7742–7751. 10.1016/j.bmc.2013.10.017 24200932

[B11] Murugaiah A. M. Wu X. Wallinder C. Mahalingam A. K. Wan Y. Skold C. (2012). From the first selective non-peptide AT_2_ receptor agonist to structurally related antagonists. J. Med. Chem. 55 (5), 2265–2278. 10.1021/jm2015099 22248302

[B12] Porrello E. R. Delbridge L. M. D. Thomas W. G. (2009). The angiotensin II type 2 (AT2) receptor: an enigmatic seven transmembrane receptor. Front. Biosci. 14 (3), 958–972. 10.2741/3289 19273111

[B13] Rice A. S. C. Dworkin R. H. McCarthy T. D. Anand P. Bountra C. McCloud P. I. (2014). EMA401, an orally administered highly selective angiotensin II type 2 receptor antagonist, as a novel treatment for postherpetic neuralgia: a randomised, double-blind, placebo-controlled phase 2 clinical trial. Lancet 383 (9929), 1637–1647. 10.1016/s0140-6736(13)62337-5 24507377

[B14] Sallander J. Wallinder C. Hallberga A. Aqvist J. Gutierrez-de-Teran H. (2016). Structural determinants of subtype selectivity and functional activity of angiotensin II receptors. Bioorg. Med. Chem. Lett. 26, 1355–1359. 10.1016/j.bmcl.2015.10.084 26810314

[B15] Smith M. T. Anand P. Rice A. S. C. (2016). Selective small molecule angiotensin II type 2 receptor antagonists for neuropathic pain: preclinical and clinical studies [J]. Pain 157, S33–S41. 10.1097/j.pain.0000000000000369 26785154

[B16] Smith M. T. Muralidharan A. (2015). Targeting angiotensin II type 2 receptor pathways to treat neuropathic pain and inflammatory pain. Expert Opin. Ther. Targets 19 (1), 25–35. 10.1517/14728222.2014.957673 25315162

[B17] Smith M. T. Wyse B. D. Edwards S. R. (2013). Small molecule angiotensin II type 2 receptor (AT_2_R) antagonists as novel analgesics for neuropathic pain: comparative pharmacokinetics, radioligand binding, and efficacy in rats [J]. Pain Med. 14 (5), 692–705. 10.1111/pme.12063 23489258

[B18] Wallinder C. Sköld C. Sundholm S. Guimond M. O. Yahiaoui S. Lindeberg G. (2019). High affinity rigidified AT_2_ receptor ligands with indane scaffolds. Medchemcomm 10 (12), 2146–2160. 10.1039/c9md00402e 32904210 PMC7451071

[B19] Wan Y. Wallinder C. Plouffe B. Beaudry H. Mahalingam A. K. Wu X. (2004). Design, synthesis, and biological evaluation of the first selective nonpeptide AT_2_ receptor agonist. J. Med. Chem. 47, 5995–6008. 10.1021/jm049715t 15537354

[B20] Whitebread S. E. Taylor V. Bottari S. P. Kamber B. de Gasparo M. (1991). Radioiodinated cgp 42111A: a novel high affinity and highly selective ligand for the characterization of angiotensin AT2 receptors. Biochem. Biophys. Res. Commun. 181 (3), 1365–1371. 10.1016/0006-291x(91)92089-3 1764088

[B21] Zhang H. Han G. W. Batyuk A. Ishchenko A. White K. L. Patel N. (2017). Structural basis for selectivity and diversity in angiotensin II receptors. Nature 544, 327–332. 10.1038/nature22035 28379944 PMC5525545

