# Supplementary material for: Design, synthesis and biological evaluation of novel biphenylsulfonamide derivatives as selective AT2 receptor antagonists
Source: Front Chem. 2022 Aug 26;10:984717. doi: 10.3389/fchem.2022.984717 (PMC9458978; doi:10.3389/fchem.2022.984717)
Supplement: Supplementary file 1 [file Presentation1.pdf]

Supporting Information

for

Design, Synthesis and Biological Evaluation of Novel Biphenylsulfonamide Derivatives as Selective AT2 Receptor Antagonists

Danhui Wang [a], Wenjie Zhao[a †], Zuzhi Zhang[a], Yanchun Zhang[* a,b], Jiaming Li[a,b], Weijun Huang[a]

[a]*College of Pharmacy, Anhui University of Chinese Medicine, Hefei, 230012, China*

[b]*Anhui Province Key Laboratory of Chinese Medicinal Formula, Hefei, Anhui, 230012, China*

* *Corresponding author: Yanchun Zhang , E-mail address: yczhang2017@163.com*

† These authors contributed equally to this work.

Table of contents:

1. Synthesis of intermediate **4a-4c**
2. NMR and HRMS spectra of compounds **8a-8l, 9a-9h**
3. Radioligand binding assay steps
4. NG108-15 cell experiment steps

1. Synthesis of intermediate 4a-4c

1.1 General procedure A: Synthesis of intermediates (2a-2c)

Isobutylbenzene, butylbenzene or butoxybenzene (74.50 mmol) was dissolved in the CH_2Cl_2 (200 mL). Subsequently, chlorosulfonic acid (298.01 mmol) was added slowly to the solution in an ice bath and the mixture was stirred at rt for 1 h. The reaction was quenched by dropped ice-cold water (50 mL) into the mixture. The reaction mixture was extracted twice with CH_2Cl_2 (20 mL) and washed with water (30 mL×2) and brine (30 mL×2). The organic layer was dried with anhydrous Na_2SO_4, filtered, and evaporated to achieve **2a-2c.**

1.2 General procedure B: Synthesis of intermediates (3a-3c)

To a 250 mL closed pressure vessel was added **2a, 2b, or 2c** (70.90 mmol) dissolved in DCM (200 mL). Tert-butylamine (11.18 mL, 106.35 mmol) was added to the mixture in an ice bath. The reaction was stirred at r.t for 8 h. The reaction was extracted with DCM (50 mL), the organic layers were combined and washed with NaCl, dried over Na_2SO_4, and concentrated to give **3a-3c**.

1.3 General procedure C: Synthesis of intermediates (4a-4c)

To a 100 mL round-bottom flask were added **3a, 3b, 3c** (7.42 mmol) followed by dry THF (20 mL). N-BuLi (1.6 M in hexane, 9.28 mL, 14.85 mmol) was added to the mixture at -78 °C.The reagent was added under nitrogen and the reaction was stirred for 1 h. After the flask was warmed to -20 °C, kept for 3 h and subsequently decreased to -78 °C. Triisopropyl borate (2.57 mL, 11.14 mmol) was then added. The reaction mixture was stirred over night at room temperature. The reaction mixture was treated with an excess of 2 M HCl solution in an ice bath. The mixture was extracted with ethyl acetate (50 mL x 2). The combined organic phase was washed with water and brine, dried with Na_2SO_4, filtered and evaporated. Using silica gel column chromatography (PE/EA as eluent), the residue was purified to obtain **4a-4c.**

2. NMR and HRMS spectra of compounds 8a-8l, 9a-9h.

^{1}H and ^{13}C NMR spectra were recorded at room temperature at 400 MHz and 100 MHz respectively using a QNP probe. NMR spectra were recorded in deuterated dimethyl sulfoxide (DMSO-d_6) at room temperature unless otherwise stated. Chemical shifts (δ values) are reported in parts per million, and are referenced to the deuterated residual Solvent peak. NMR data was reported as: δ value (chemical shift, *J*-value(Hz), integration, where s = singlet, d = doublet, t = triplet, q = quartet, brs = broad singlet). High-resolution mass spectra (HRMS) were recorded with a Bruker microTOF ESI-TOF mass spectrometer in positive ion mode unless otherwise specified.

8a:

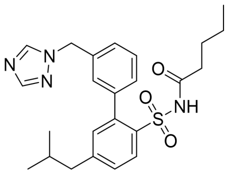

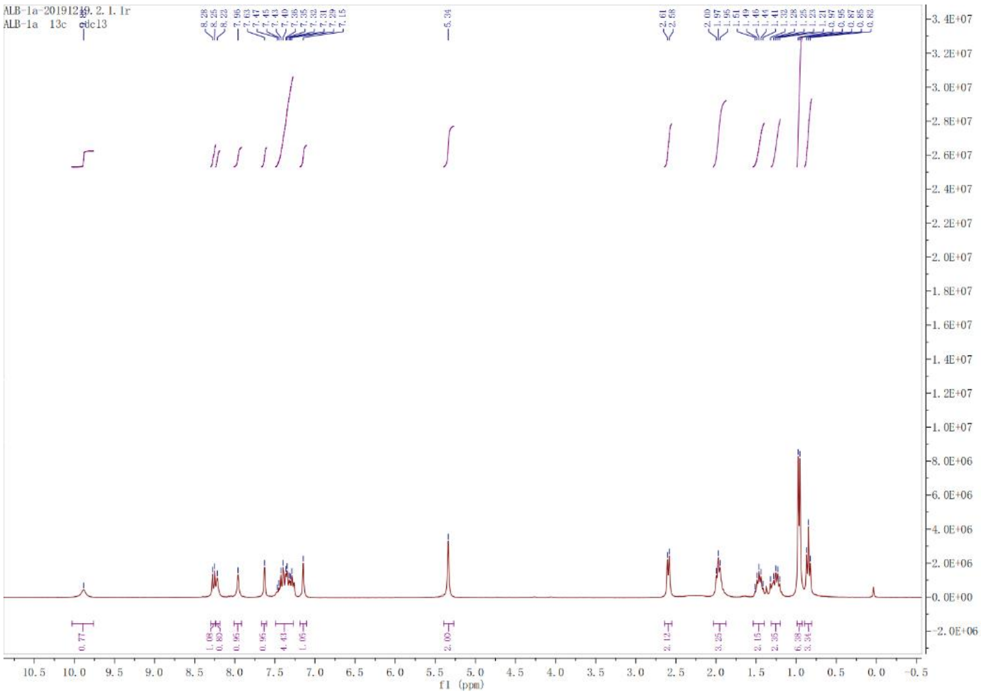

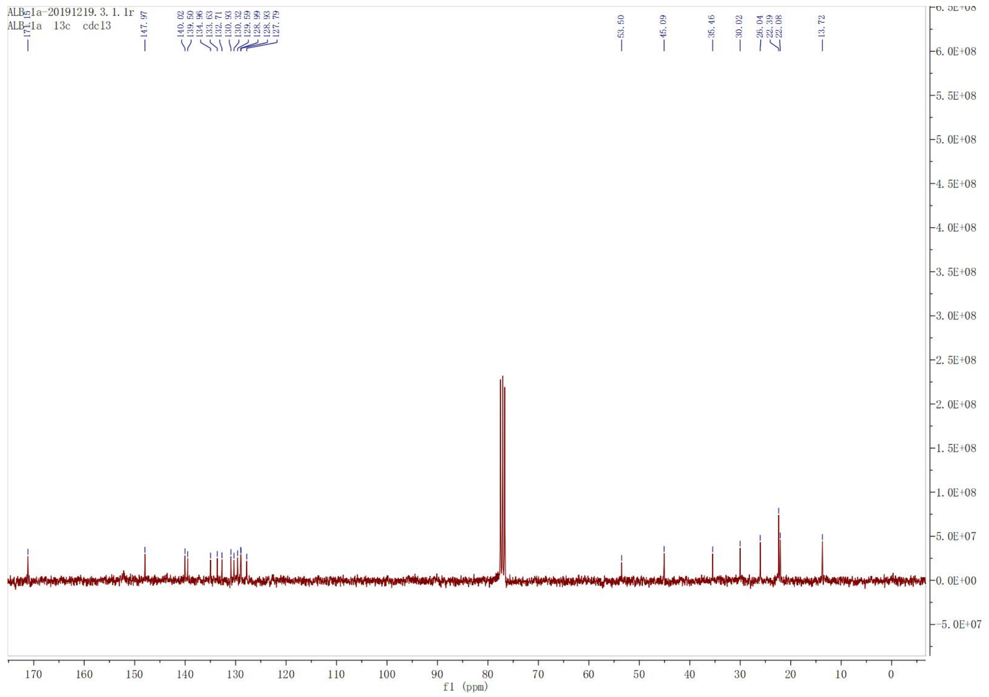
ALB-1a-20191219.3.1.1r
ALB-1a 13c cdcl3
171.15
147.97
140.02
139.50
134.96
133.63
132.71
130.93
130.32
129.59
128.99
128.93
127.79
53.50
45.09
35.46
30.02
26.04
22.39
22.08
13.72
f1 (ppm)

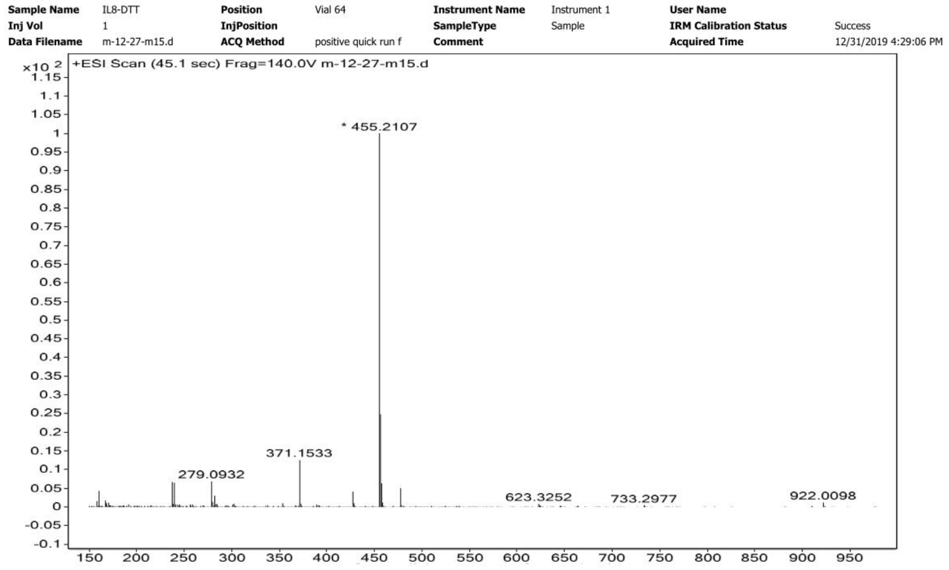
Sample Name IL8-DTT
Position Vial 64
Instrument Name Instrument 1
User Name
Inj Vol 1
InjPosition
SampleType Sample
IRM Calibration Status Success
Data Filename m-12-27-m15.d
ACQ Method positive quick run f
Comment
Acquired Time 12/31/2019 4:29:06 PM
+ESI Scan (45.1 sec) Frag=140.0V m-12-27-m15.d
x10 2
* 455.2107
371.1533
279.0932
623.3252
733.2977
922.0098

8b:

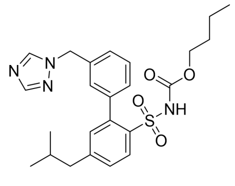
N
N
N
O
O
NH
S
O
O

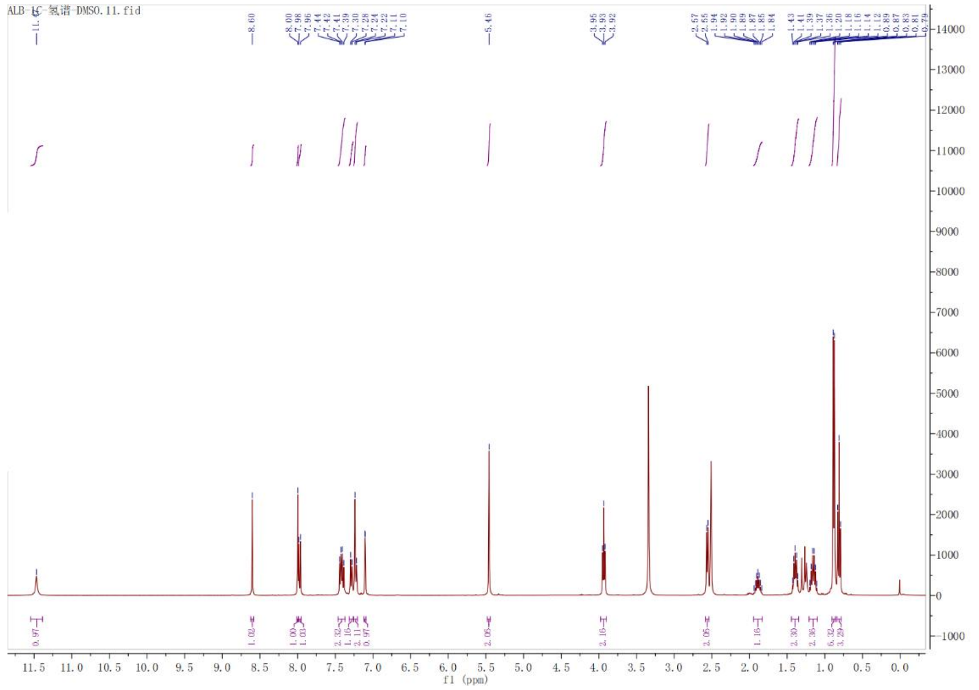
ALB-1C-氢谱-DMSO.11.fid
f1 (ppm)

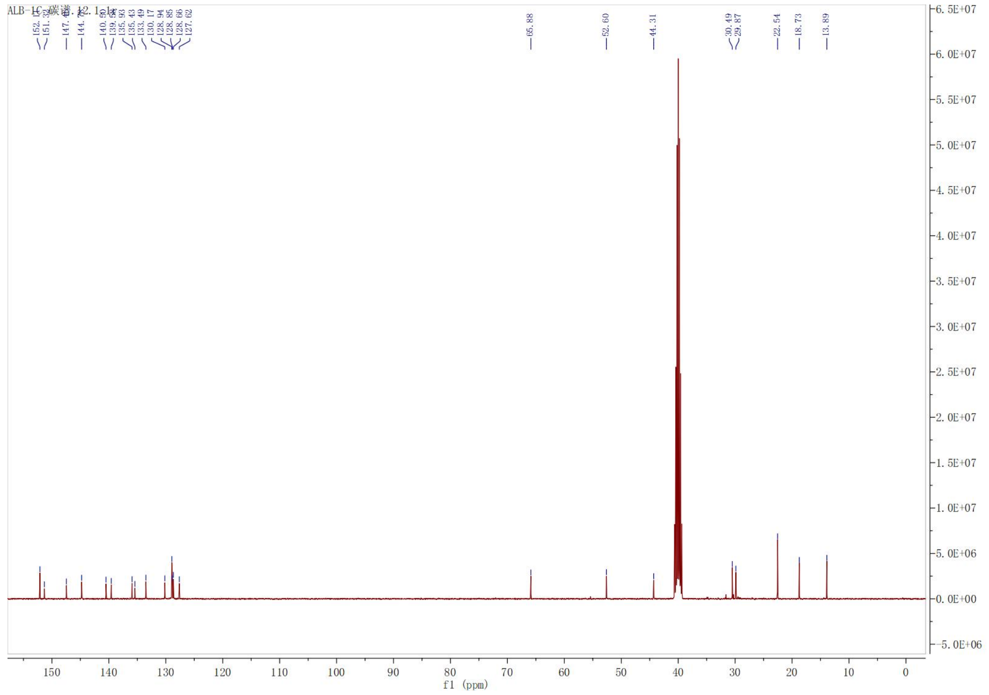
ALB-1C-碳谱.12.1.1r
f1 (ppm)

**Sample Name**	04026	**Position**	Vial 65	**Instrument Name**	Instrument 1	**User Name**	
**Inj Vol**	1	**InjPosition**		**SampleType**	Sample	**IRM Calibration Status**	Success
**Data Filename**	m-12-27-m16.d	**ACQ Method**	positive quick run f	**Comment**		**Acquired Time**	12/31/2019 4:32:30 PM

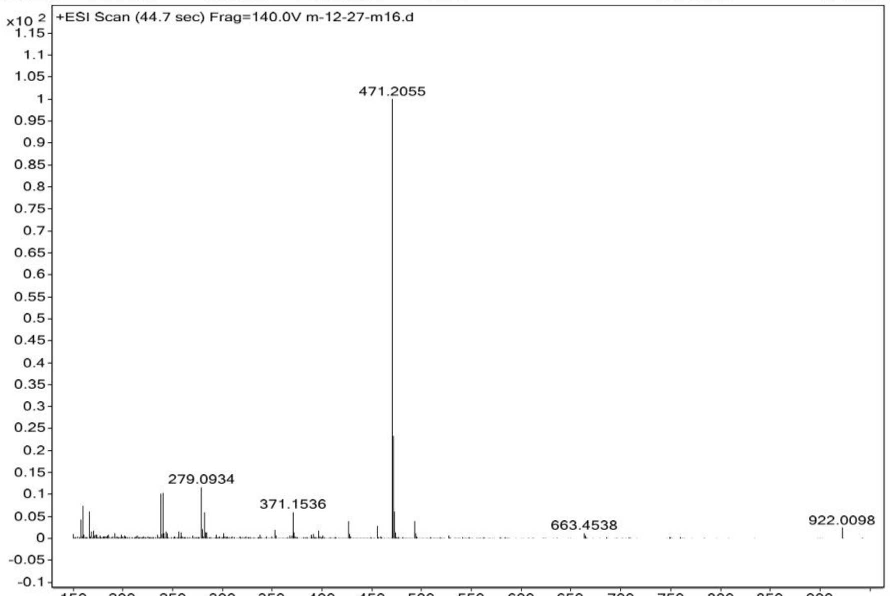

8c:

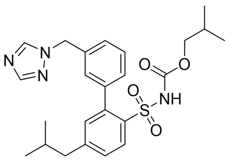

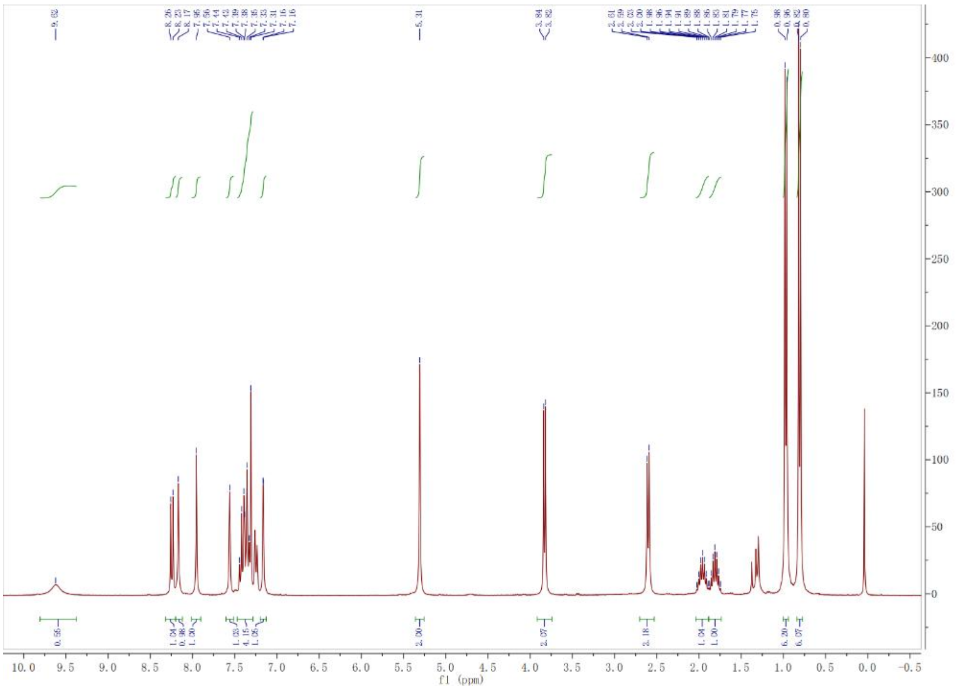

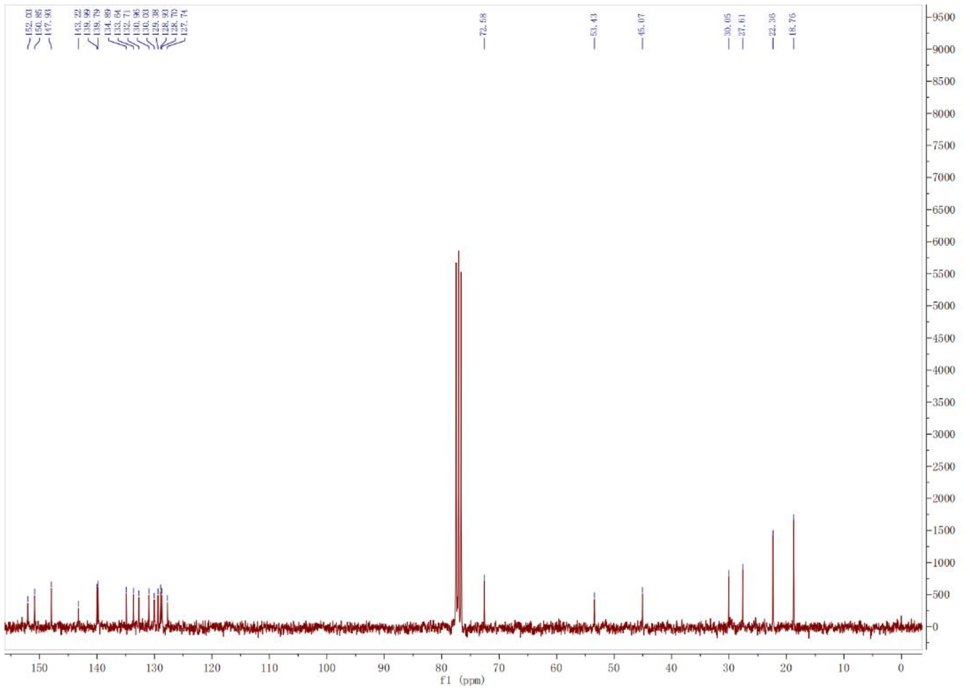
152.03
150.85
147.93
143.22
139.99
139.79
134.89
133.64
132.71
130.96
130.03
129.38
128.93
128.70
127.74
72.58
53.43
45.07
30.05
27.61
22.36
18.76
f1 (ppm)

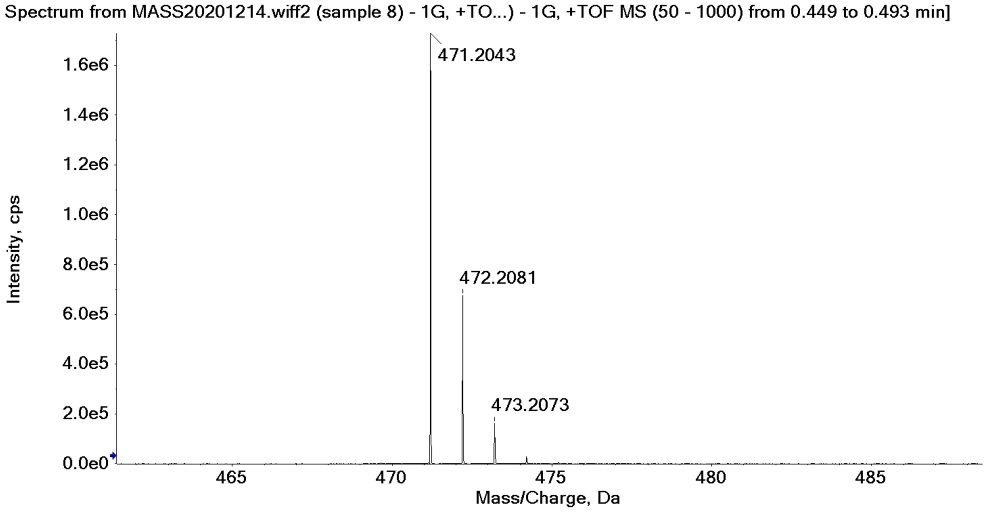
Spectrum from MASS20201214.wiff2 (sample 8) - 1G, +TO...) - 1G, +TOF MS (50 - 1000) from 0.449 to 0.493 min]
471.2043
472.2081
473.2073
Intensity, cps
Mass/Charge, Da

8d:

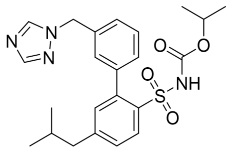
N
N
N
O
O
O
S
NH
O

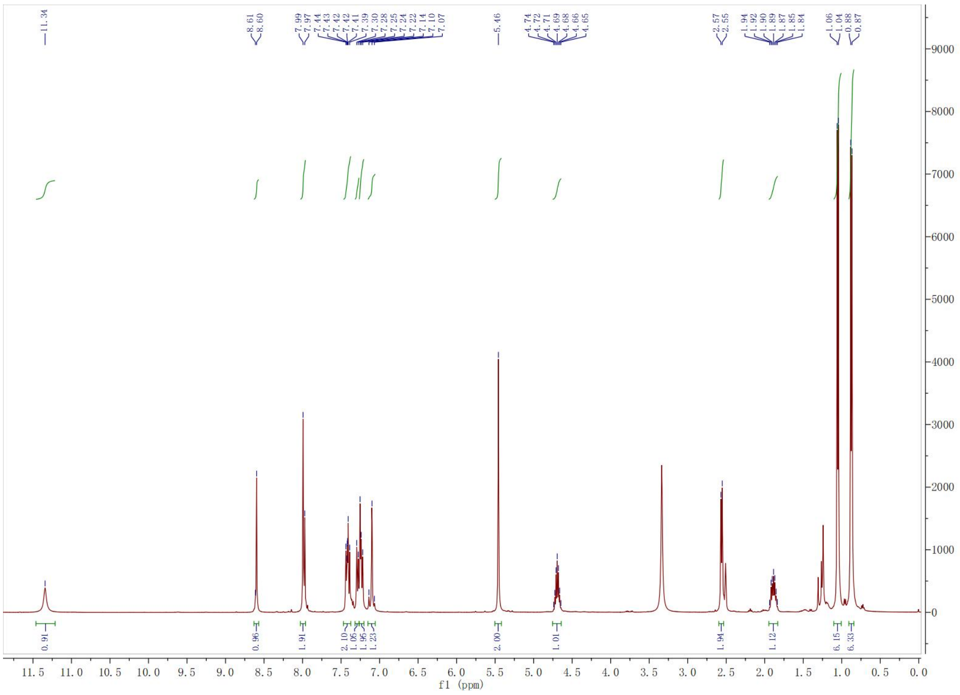

11.34
8.61
8.60
7.99
7.97
5.46
2.57
2.55
1.06
1.04
0.88
0.87
0.91
0.96
1.91
2.00
1.01
1.94
1.12
6.15
6.33
f1 (ppm)

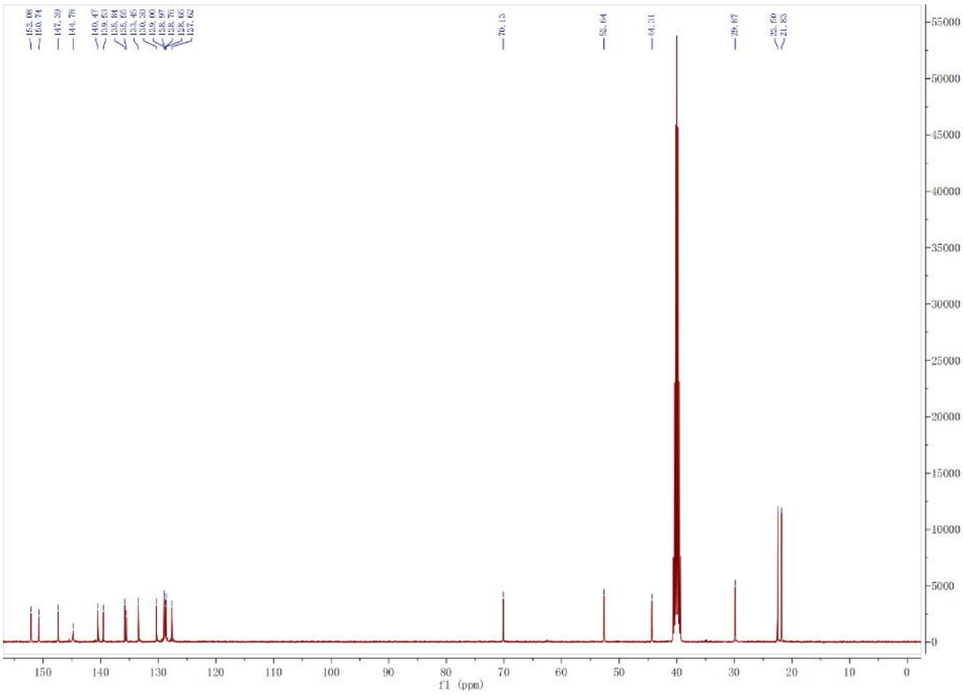

152.08
150.74
147.39
144.78
140.47
70.13
52.64
44.31
29.87
f1 (ppm)

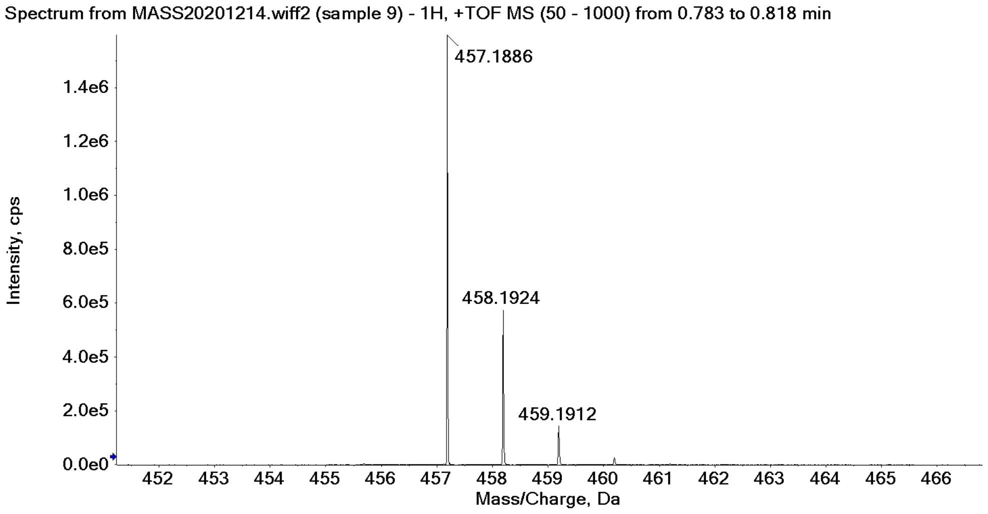

Spectrum from MASS20201214.wiff2 (sample 9) - 1H, +TOF MS (50 - 1000) from 0.783 to 0.818 min
457.1886
458.1924
459.1912
Intensity, cps
1.4e6
1.2e6
1.0e6
8.0e5
6.0e5
4.0e5
2.0e5
0.0e0
452 453 454 455 456 457 458 459 460 461 462 463 464 465 466
Mass/Charge, Da

8e:

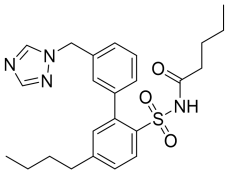

N
N
N
O
O
S
NH
O

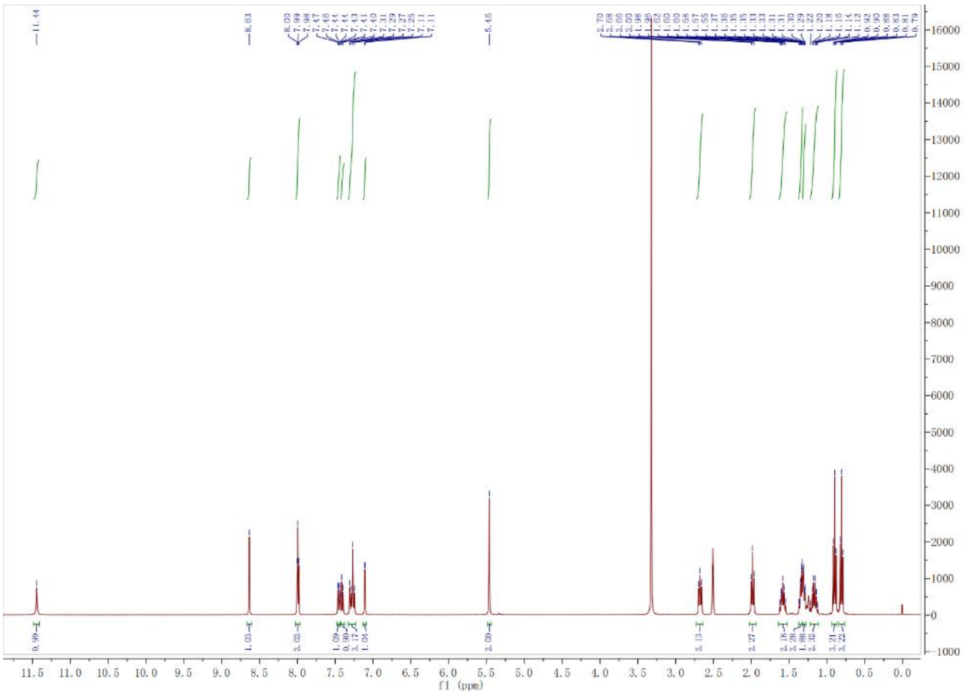

11.5 11.0 10.5 10.0 9.5 9.0 8.5 8.0 7.5 7.0 6.5 6.0 5.5 5.0 4.5 4.0 3.5 3.0 2.5 2.0 1.5 1.0 0.5 0.0
f1 (ppm)

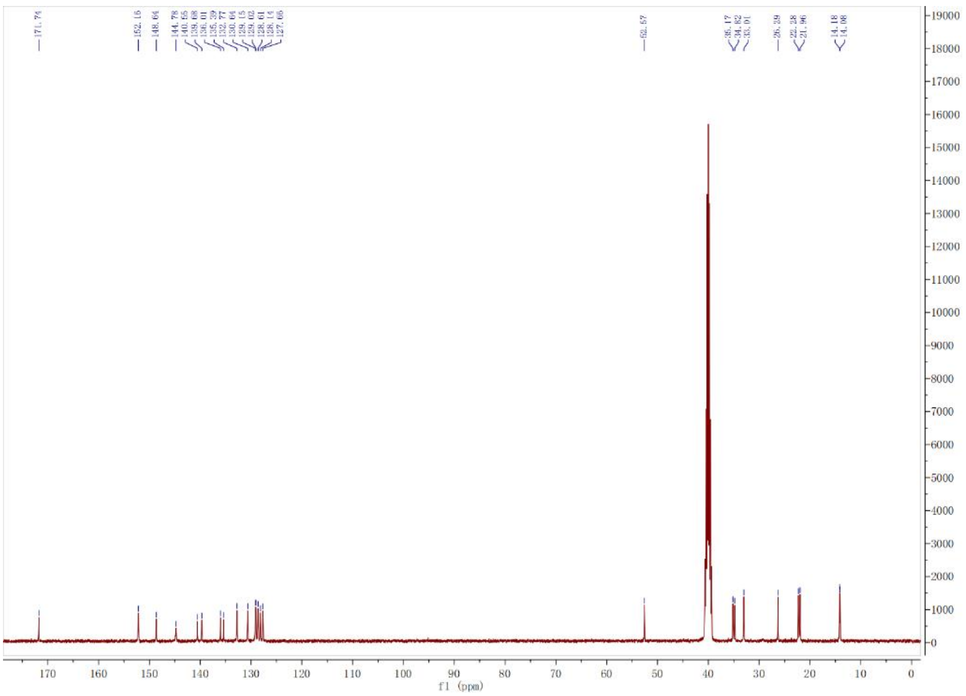
f1 (ppm)

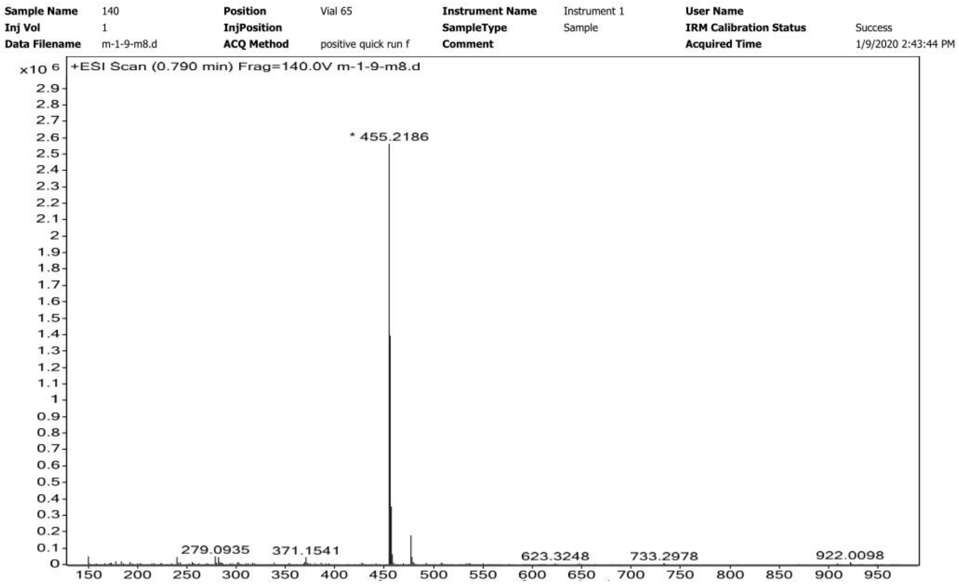
Sample Name 140
Position Vial 65
Instrument Name Instrument 1
User Name
Inj Vol 1
InjPosition
SampleType Sample
IRM Calibration Status Success
Data Filename m-1-9-m8.d
ACQ Method positive quick run f
Comment
Acquired Time 1/9/2020 2:43:44 PM
+ESI Scan (0.790 min) Frag=140.0V m-1-9-m8.d
x10 6
* 455.2186
279.0935
371.1541
623.3248
733.2978
922.0098

8f:

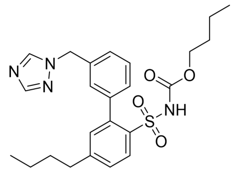
N
N
N
O
O
NH
S
O
O

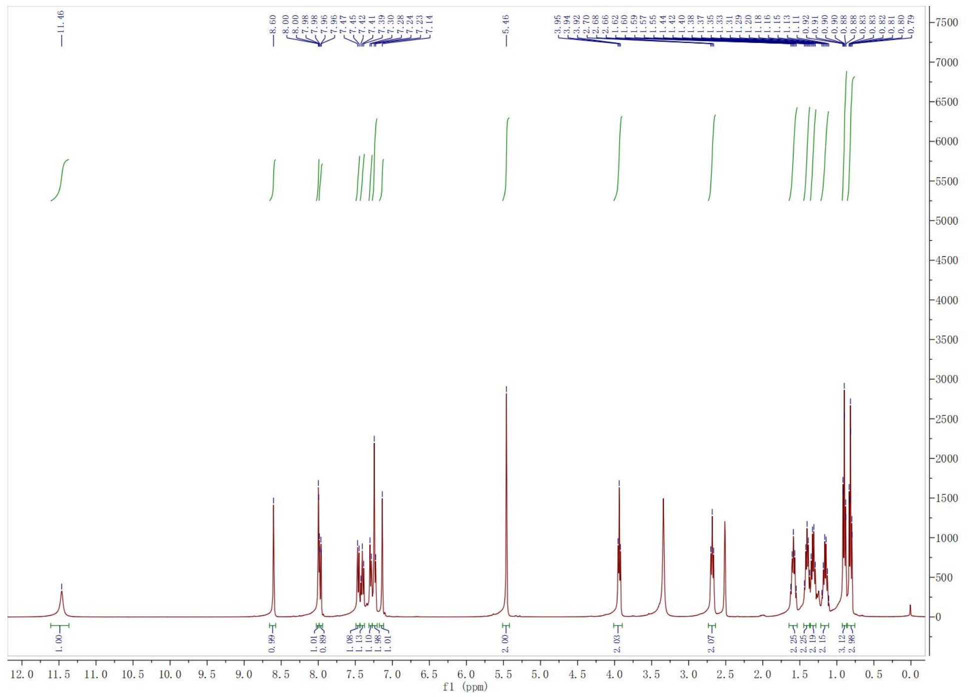
f1 (ppm)

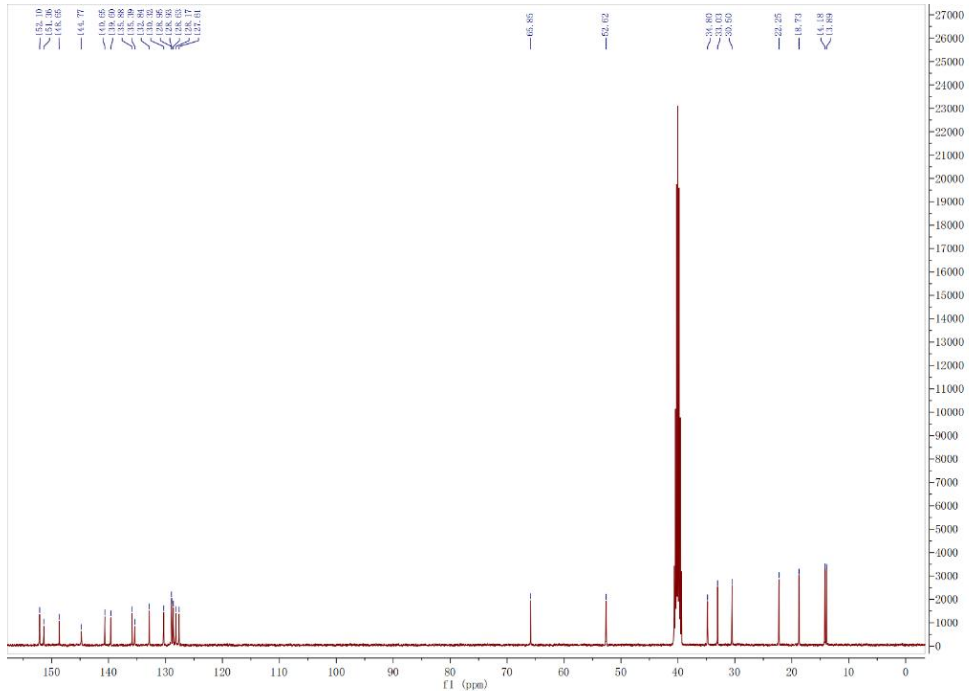
f1 (ppm)

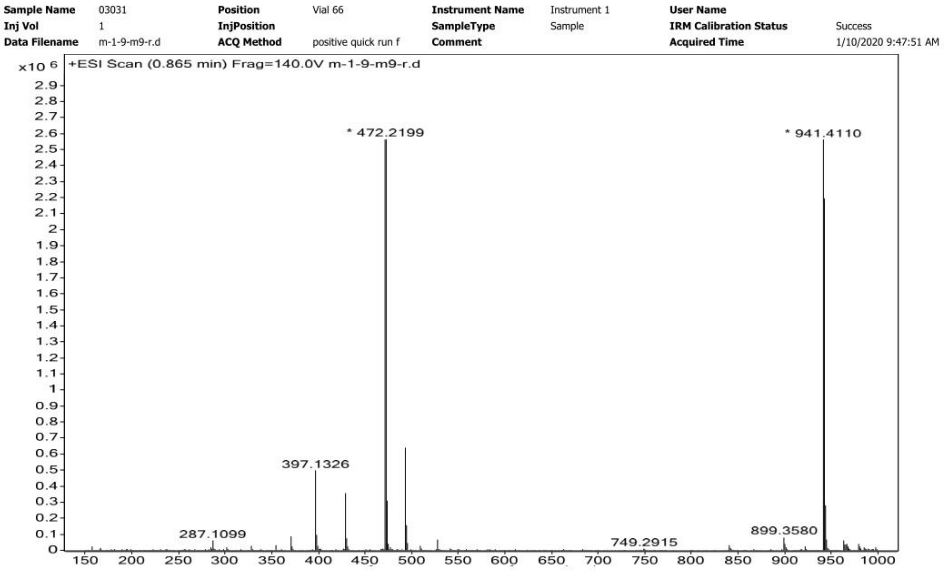
Sample Name 03031
Position Vial 66
Instrument Name Instrument 1
User Name
Inj Vol 1
InjPosition
SampleType Sample
IRM Calibration Status Success
Data Filename m-1-9-m9-r.d
ACQ Method positive quick run f
Comment
Acquired Time 1/10/2020 9:47:51 AM
x10 6
+ESI Scan (0.865 min) Frag=140.0V m-1-9-m9-r.d
* 472.2199
* 941.4110
397.1326
287.1099
749.2915
899.3580

8g:

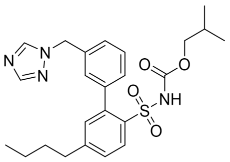

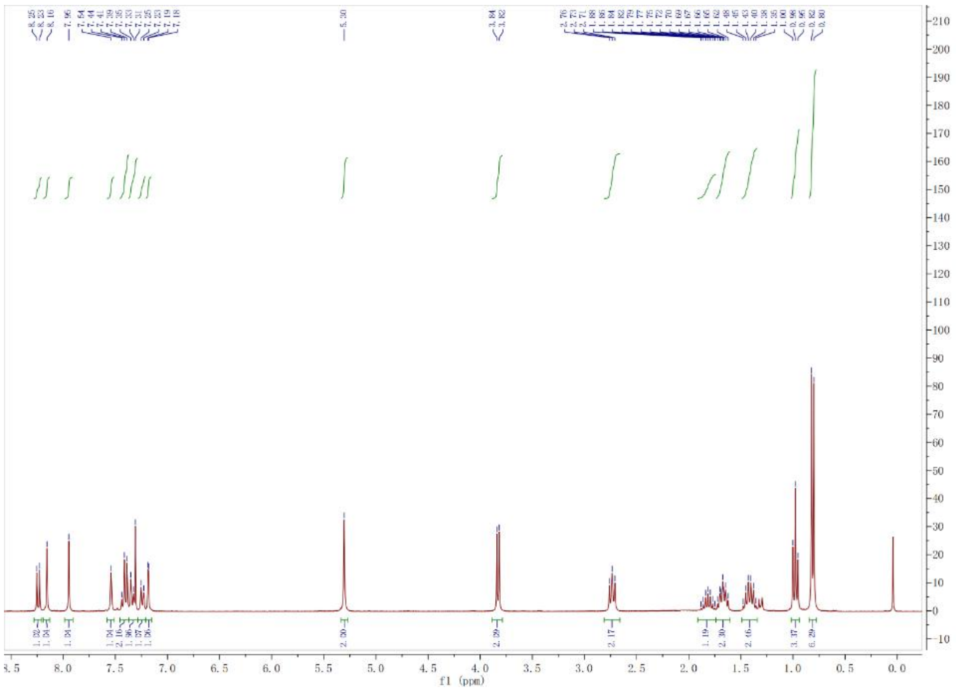
f1 (ppm)

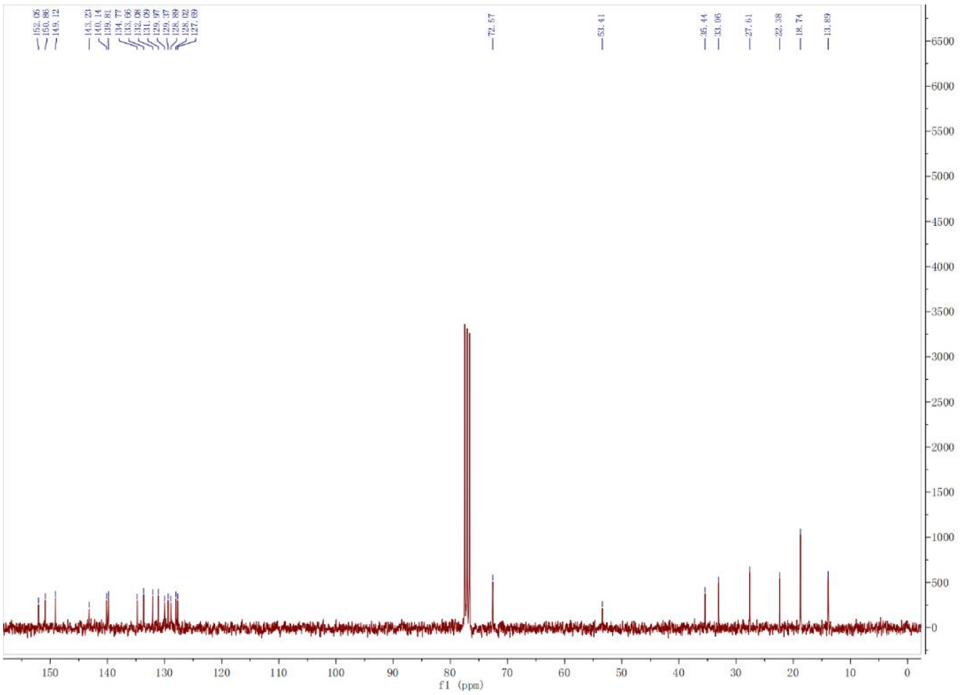
152.05
150.86
149.12
143.23
140.14
139.81
134.77
133.66
132.08
131.09
129.97
129.37
128.89
128.02
127.69
72.57
53.41
35.44
33.06
27.61
22.38
18.74
13.89
f1 (ppm)

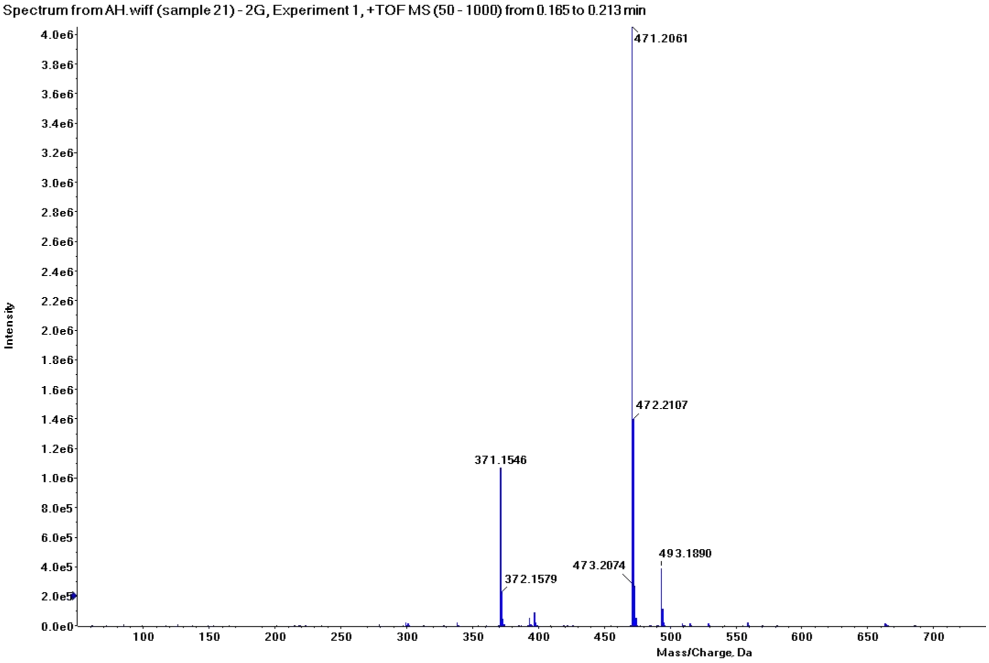
Spectrum from AH.wiff (sample 21) - 2G, Experiment 1, +TOF MS (50 - 1000) from 0.165 to 0.213 min
471.2061
472.2107
371.1546
493.1890
473.2074
372.1579
Intensity
Mass/Charge, Da

8h:

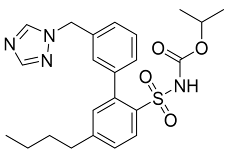
N
N
N
O
O
NH
O
S
O

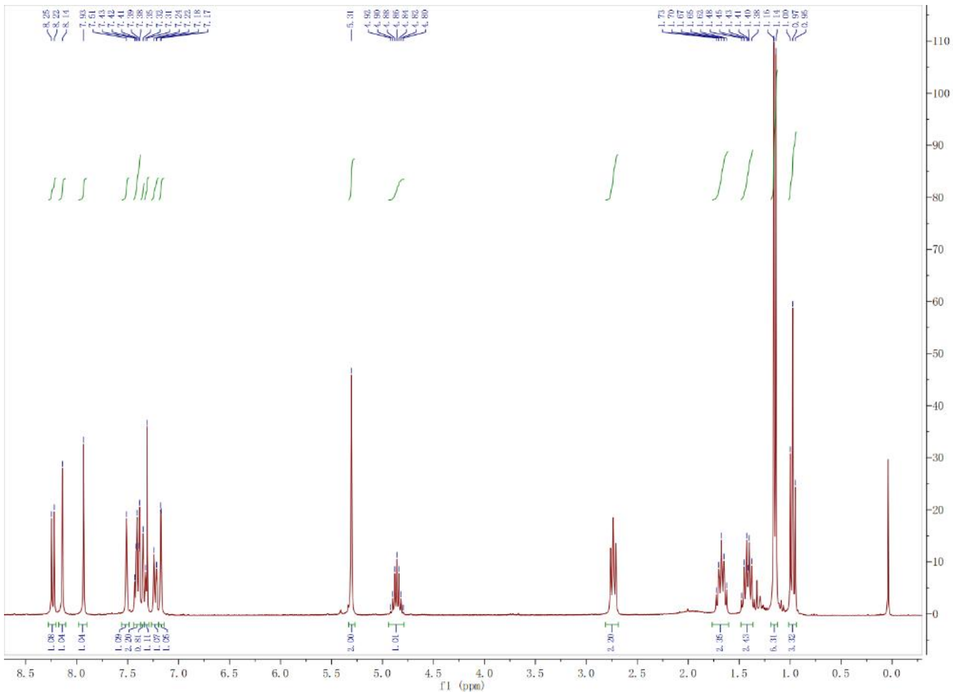
f1 (ppm)

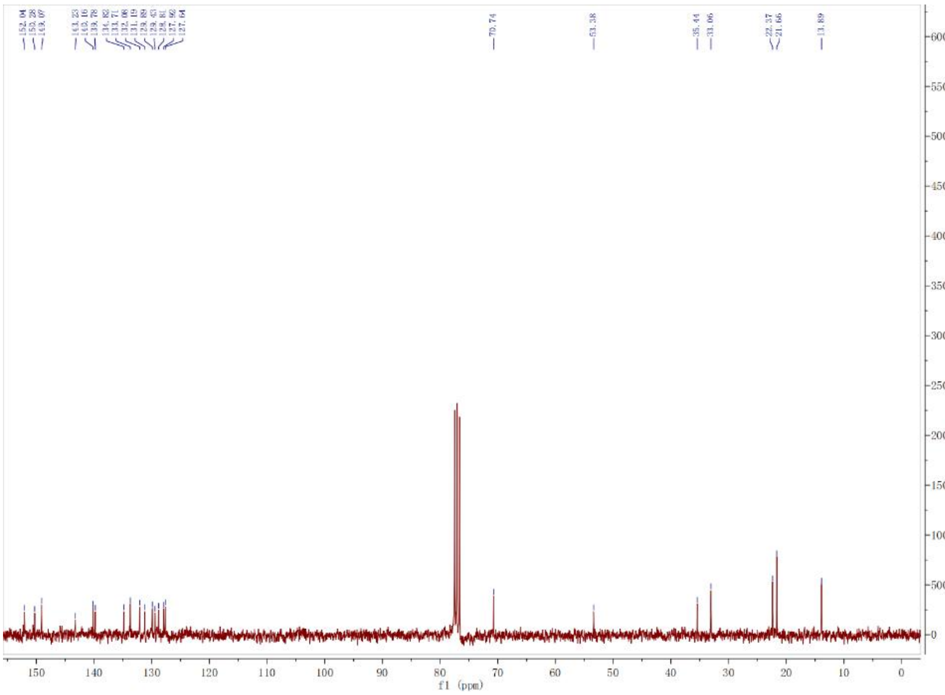
f1 (ppm)

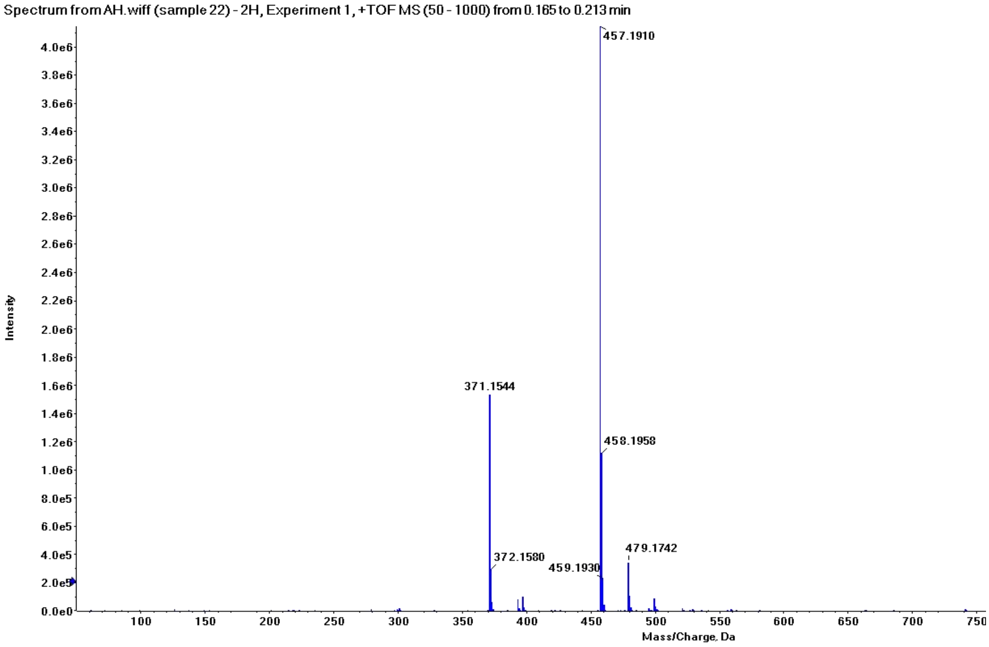
Spectrum from AH.wiff (sample 22) - 2H, Experiment 1, +TOF MS (50 - 1000) from 0.165 to 0.213 min
457.1910
371.1544
458.1958
372.1580
459.1930
479.1742
Intensity
Mass/Charge, Da

8i:

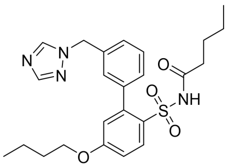
N
N
N
O
O
S
NH
O
O

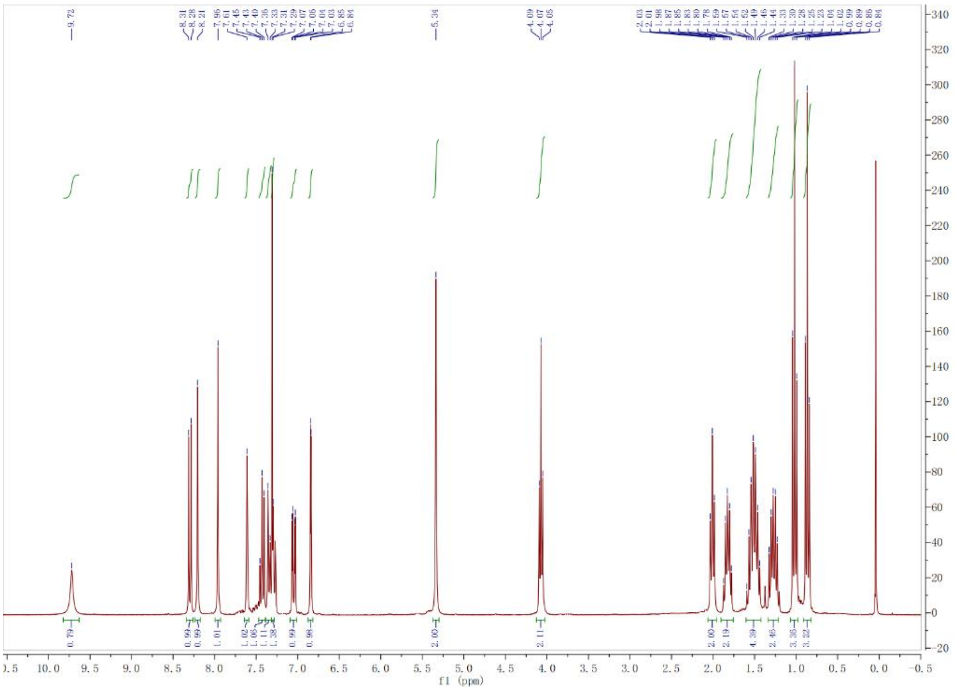
f1 (ppm)

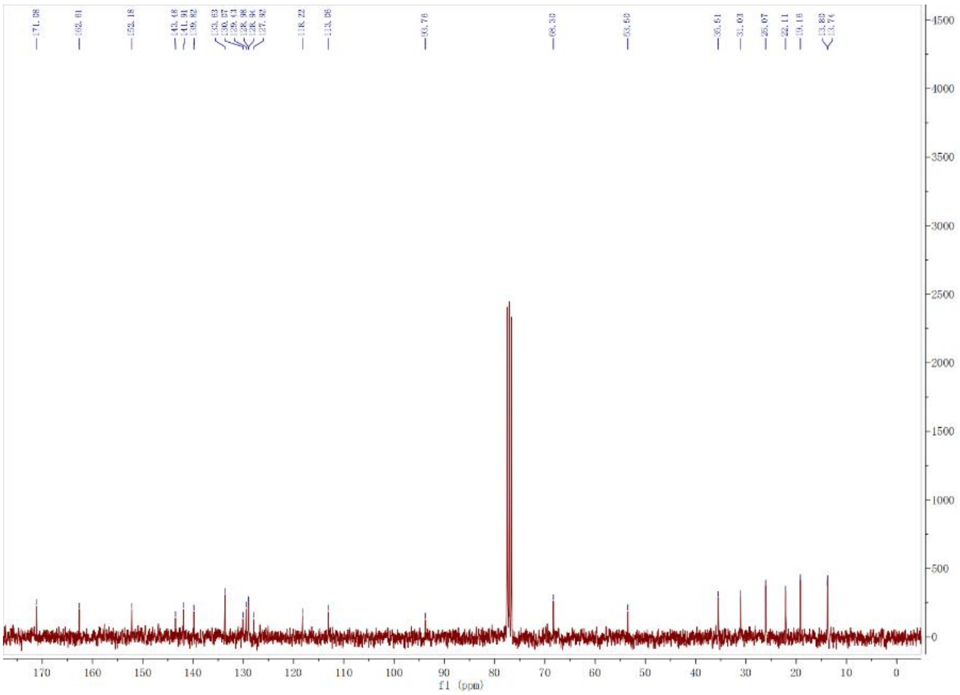
f1 (ppm)

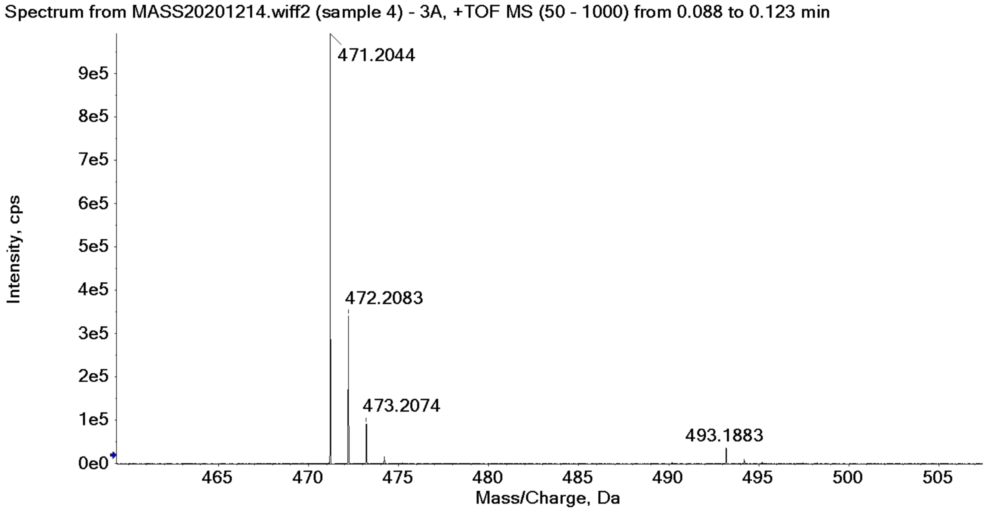
Spectrum from MASS20201214.wiff2 (sample 4) - 3A, +TOF MS (50 - 1000) from 0.088 to 0.123 min
471.2044
472.2083
473.2074
493.1883
Intensity, cps
Mass/Charge, Da

8j:

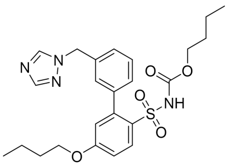
N
N
N
O
O
NH
O
S
O
O

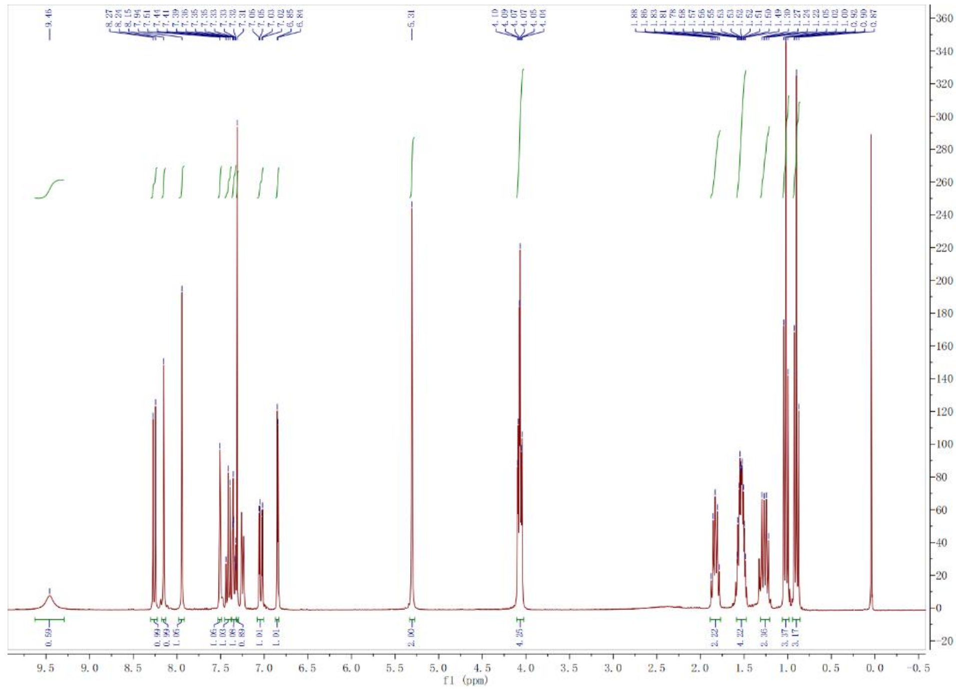

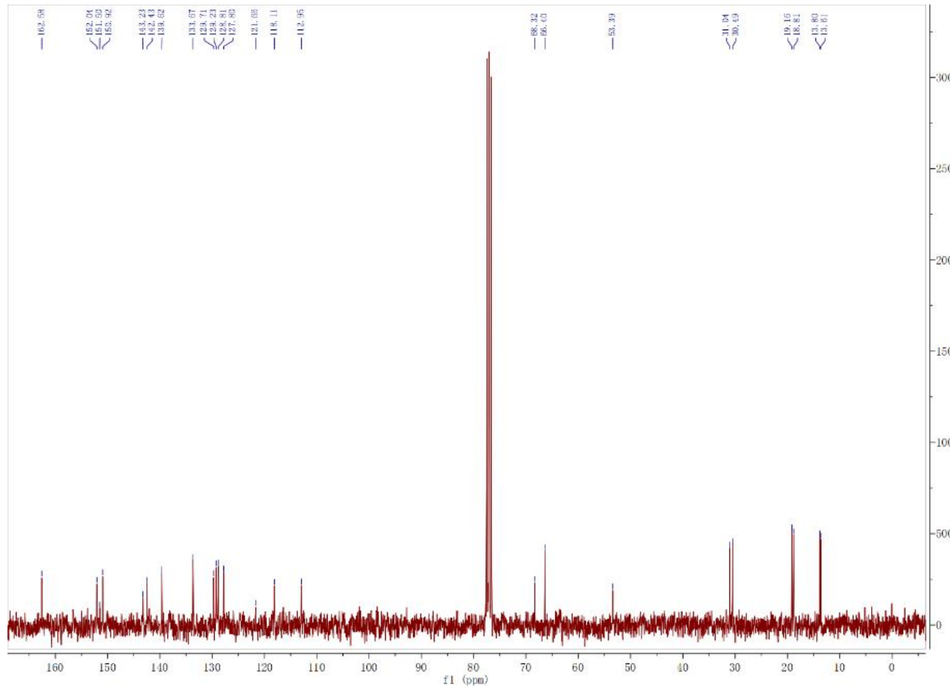

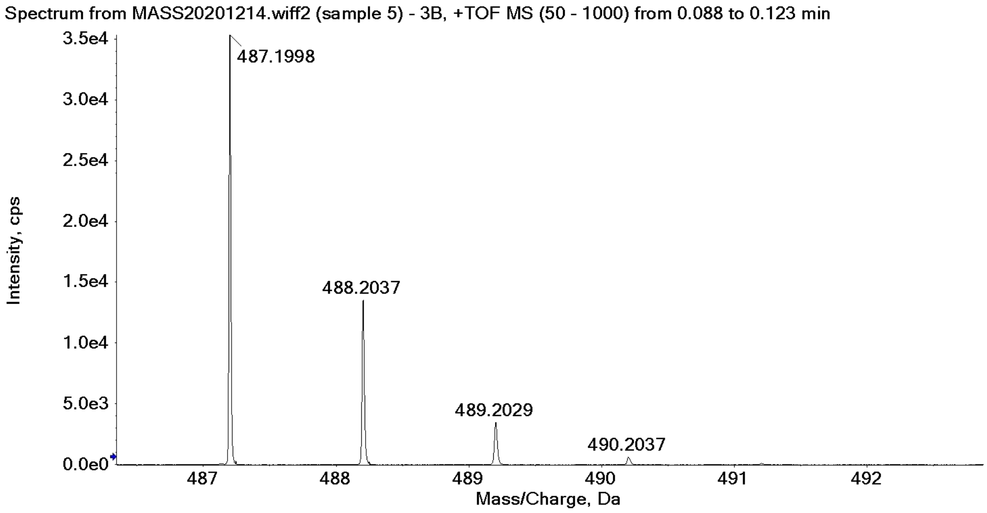

Spectrum from MASS20201214.wiff2 (sample 5) - 3B, +TOF MS (50 - 1000) from 0.088 to 0.123 min
487.1998
488.2037
489.2029
490.2037
Intensity, cps
Mass/Charge, Da

8k:

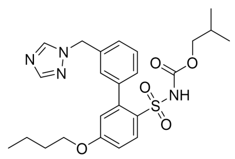

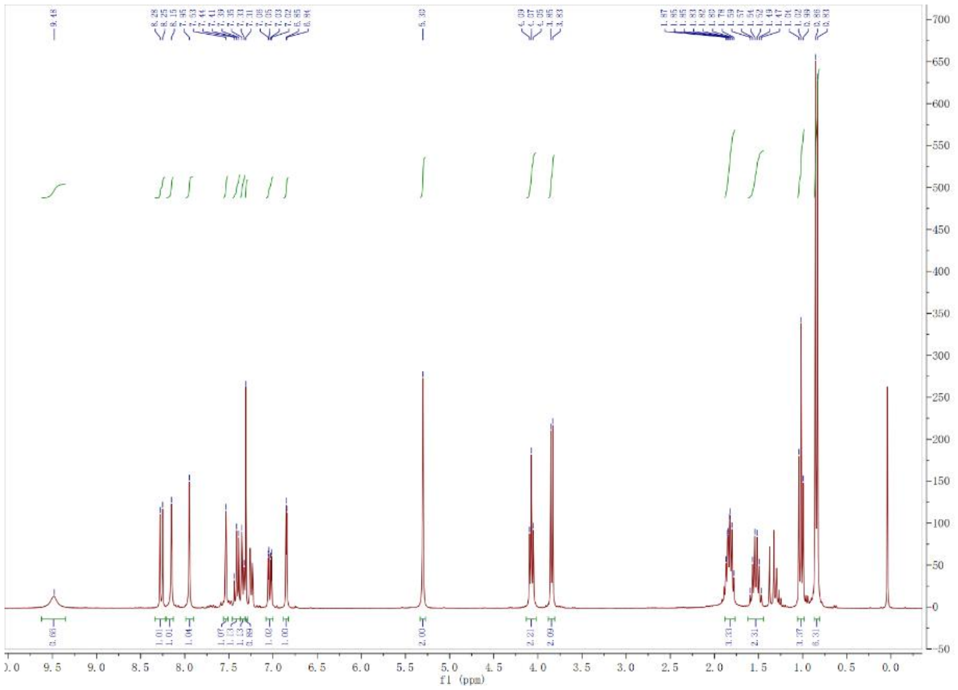

f1 (ppm)

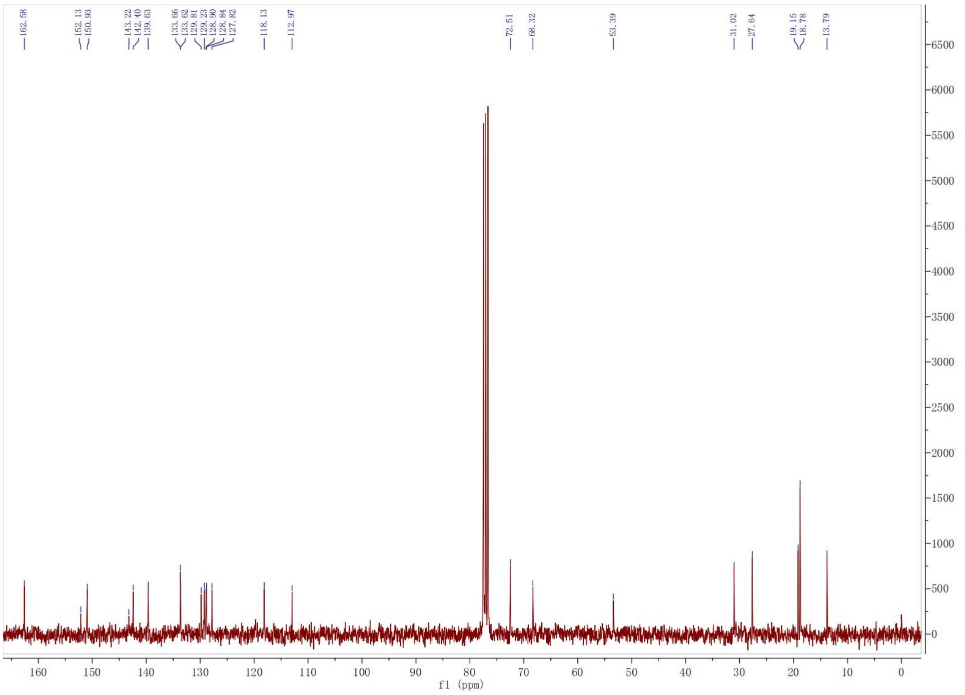

Spectrum from MASS20201214.wiff2 (sample 6) - 3C, +TO...6) - 3C, +TOF MS (50 - 1000) from 0.766 to 0.818 min]

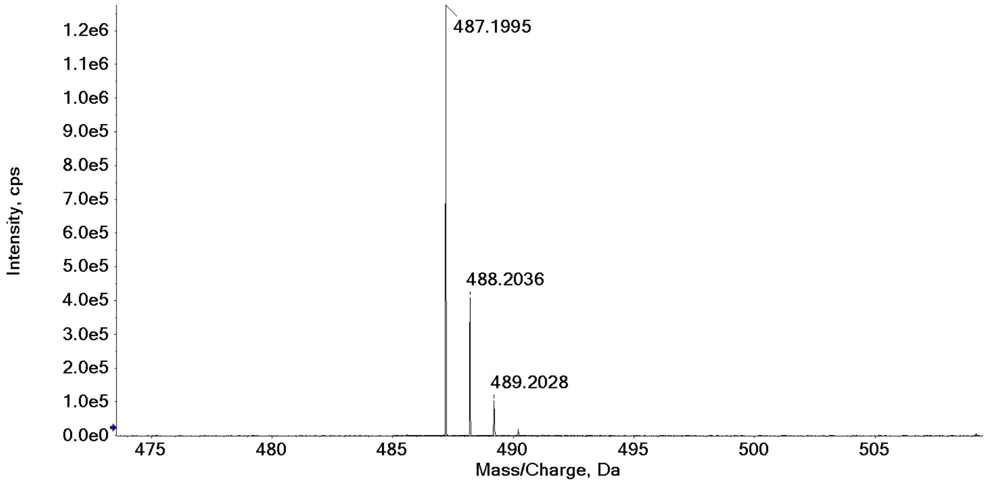

8l:

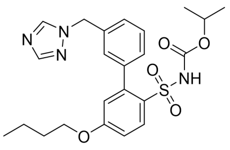

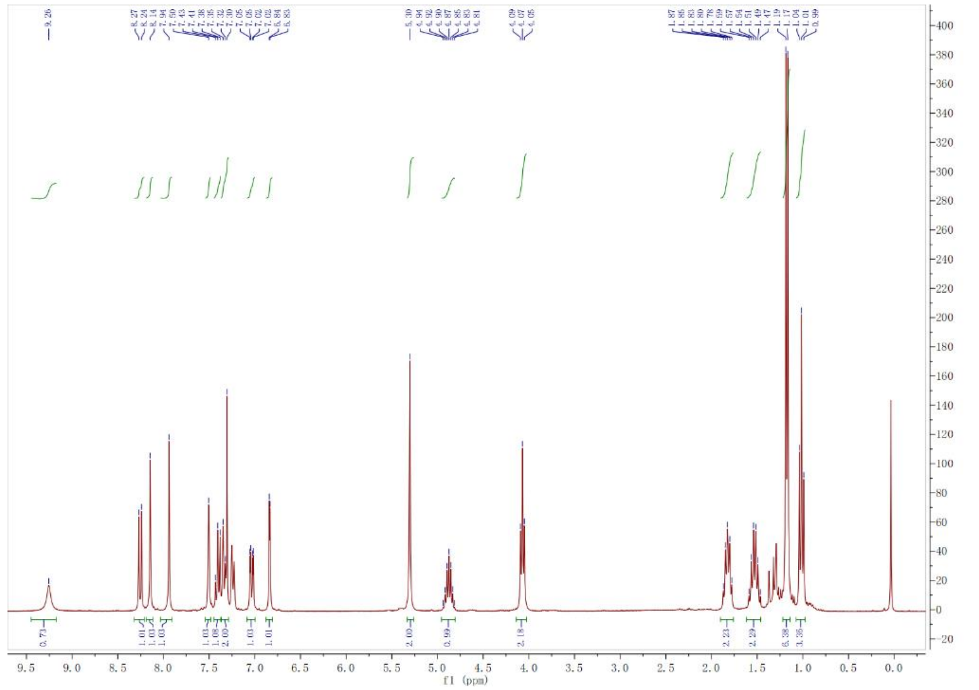
f1 (ppm)

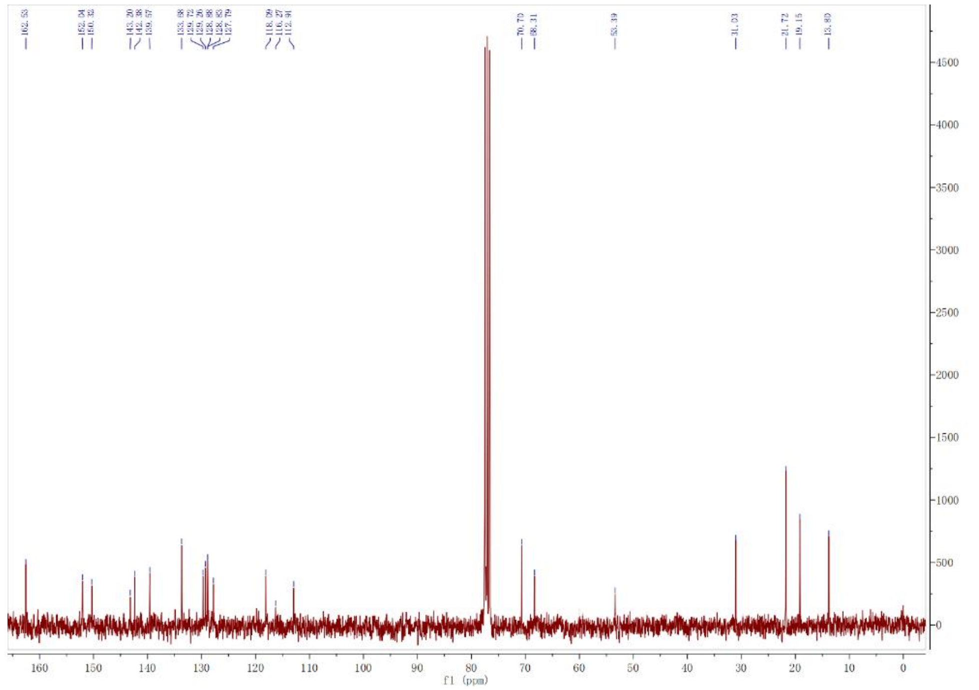
f1 (ppm)

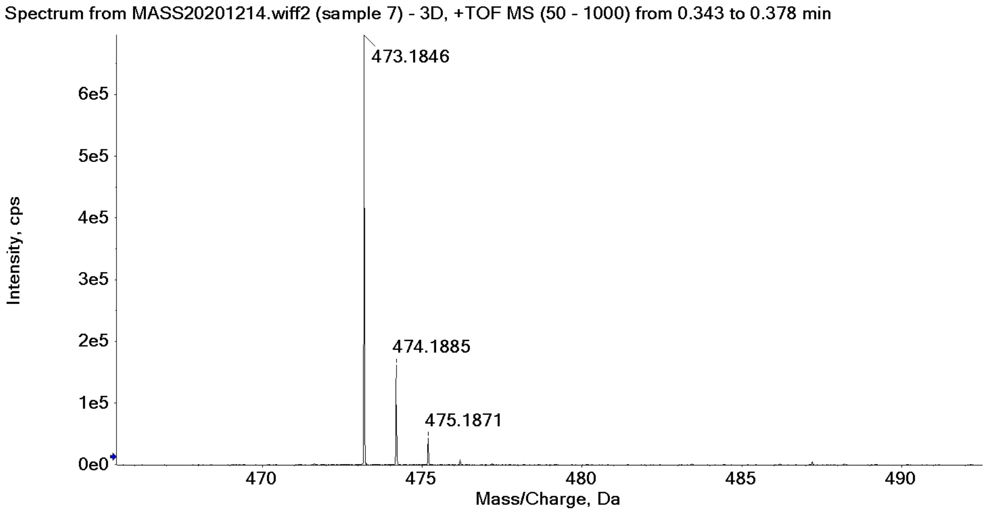
Spectrum from MASS20201214.wiff2 (sample 7) - 3D, +TOF MS (50 - 1000) from 0.343 to 0.378 min
473.1846
474.1885
475.1871
Intensity, cps
Mass/Charge, Da

9a:

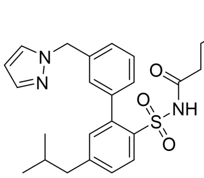

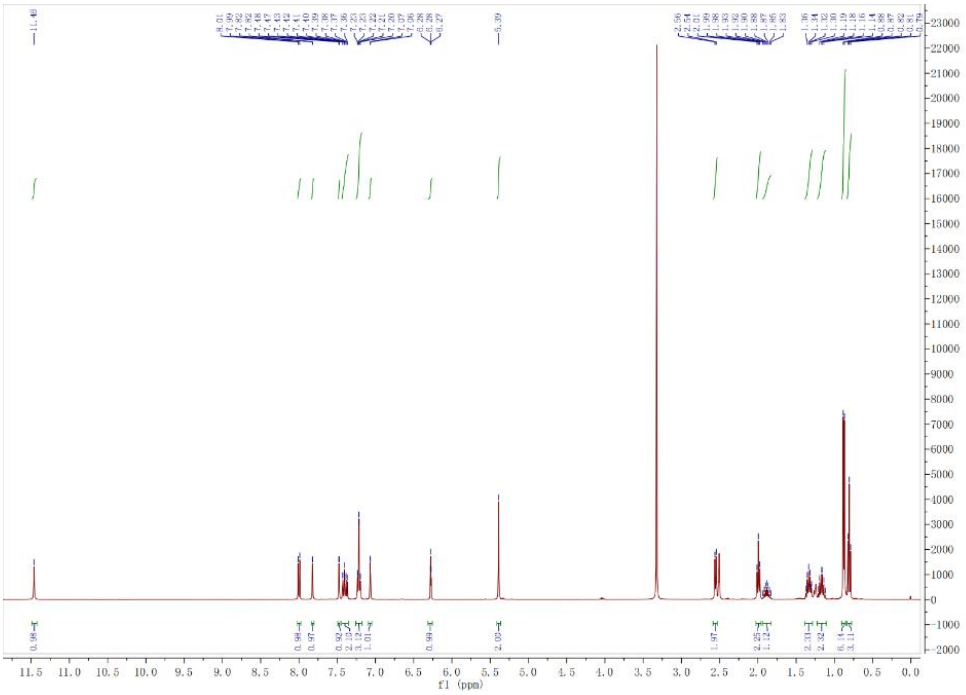
f1 (ppm)

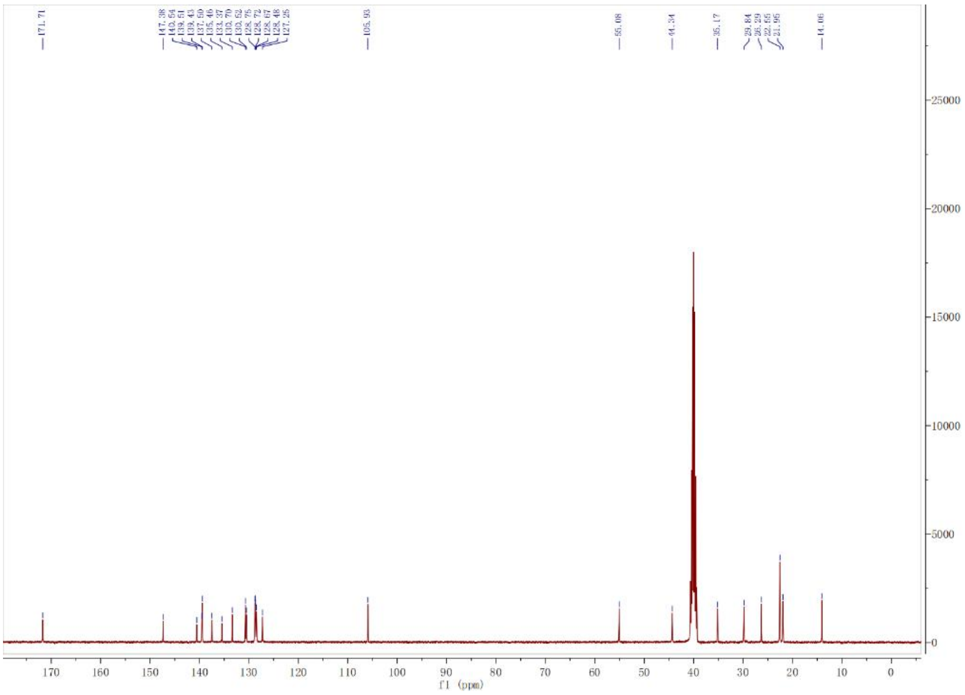
f1 (ppm)

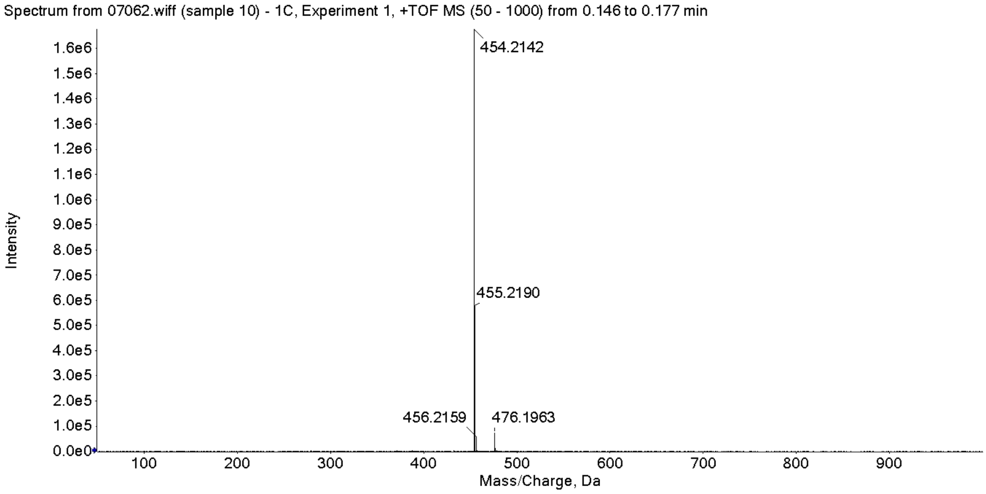
Spectrum from 07062.wiff (sample 10) - 1C, Experiment 1, +TOF MS (50 - 1000) from 0.146 to 0.177 min
454.2142
455.2190
456.2159
476.1963
Intensity
Mass/Charge, Da

9b:

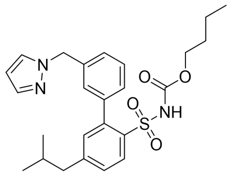
N
N
O
O
NH
S
O
O

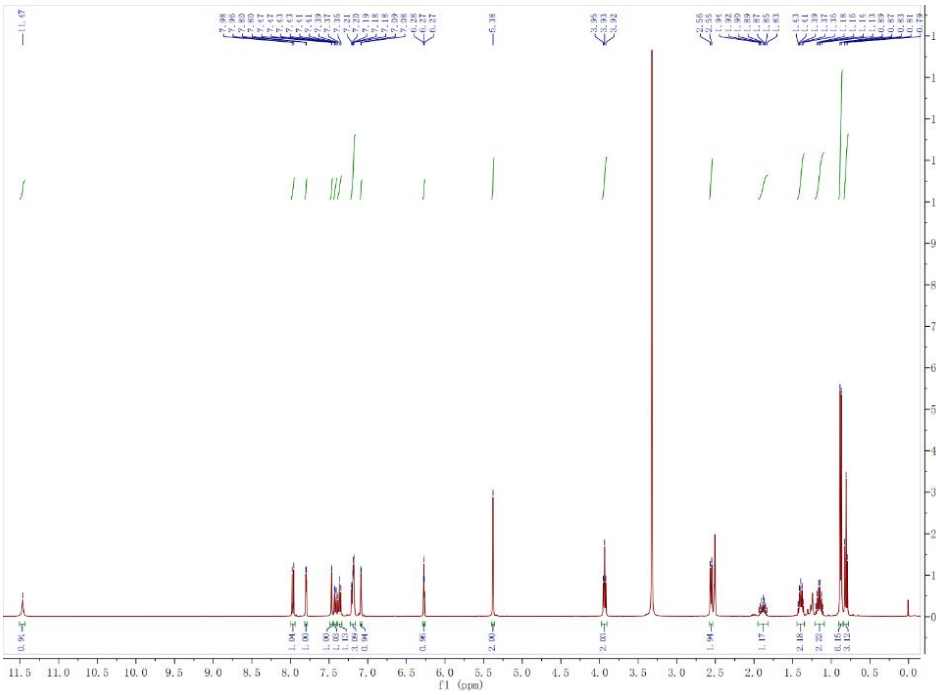
11.47
5.38
f1 (ppm)

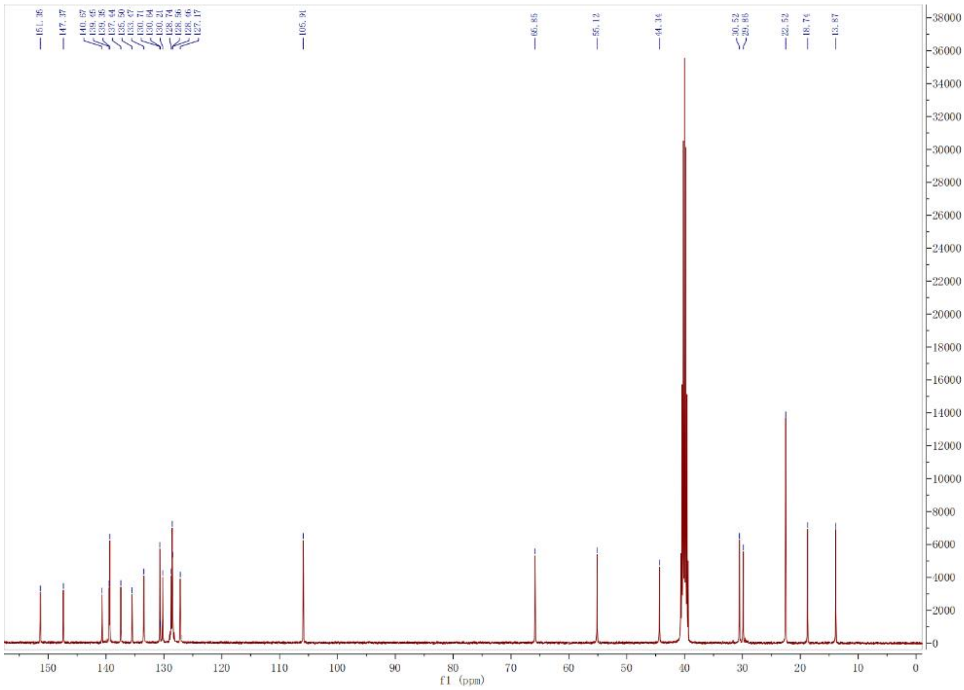
151.35
147.37
105.91
65.85
55.12
44.14
30.52
29.85
22.52
18.74
13.87
f1 (ppm)

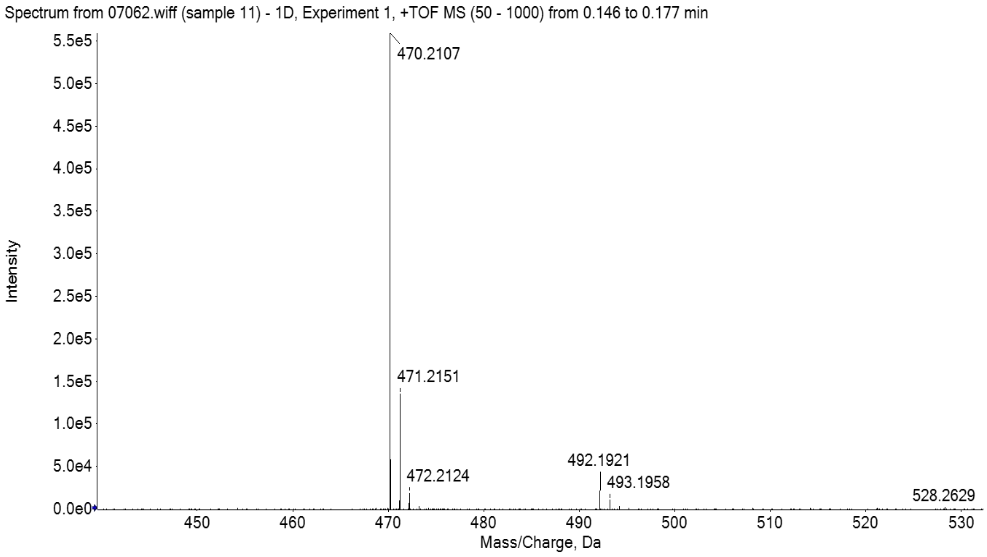
Spectrum from 07062.wiff (sample 11) - 1D, Experiment 1, +TOF MS (50 - 1000) from 0.146 to 0.177 min
Intensity
Mass/Charge, Da
470.2107
471.2151
472.2124
492.1921
493.1958
528.2629

9c:

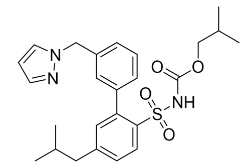
N
N
O
O
S
NH
O
O

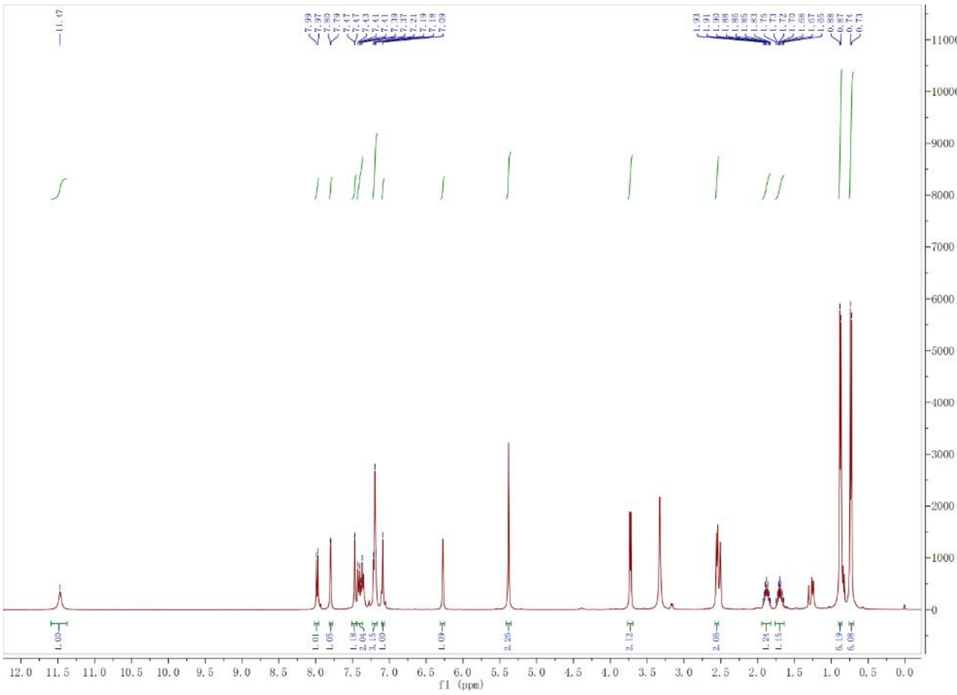
f1 (ppm)

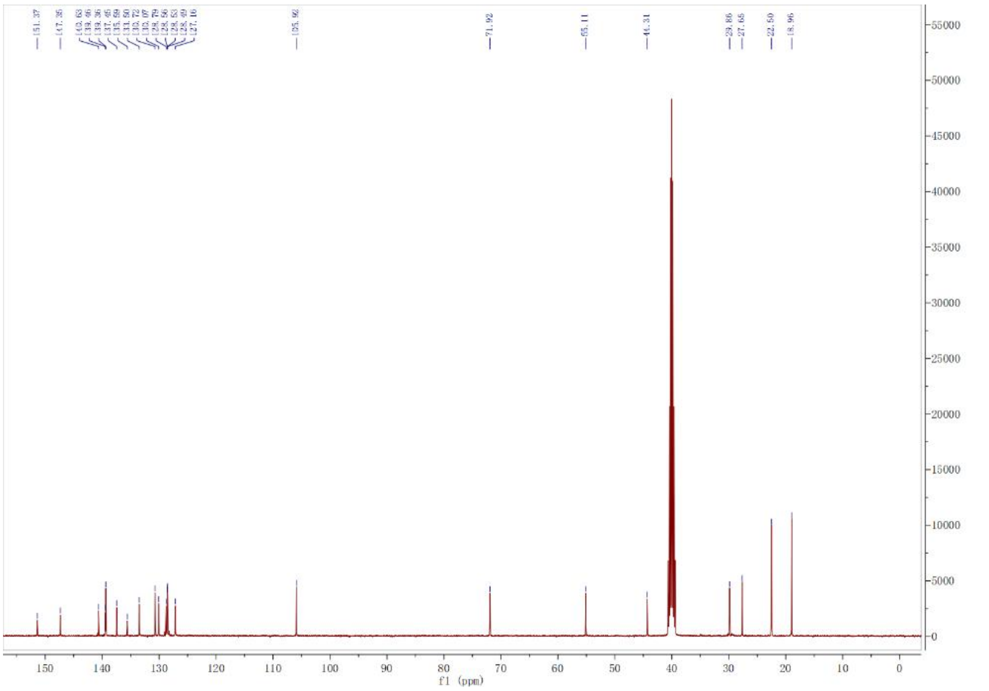

Spectrum from 07062.wiff (sample 12) - 1E, Experiment 1, +TOF MS (50 - 1000) from 0.145 to 0.177 min

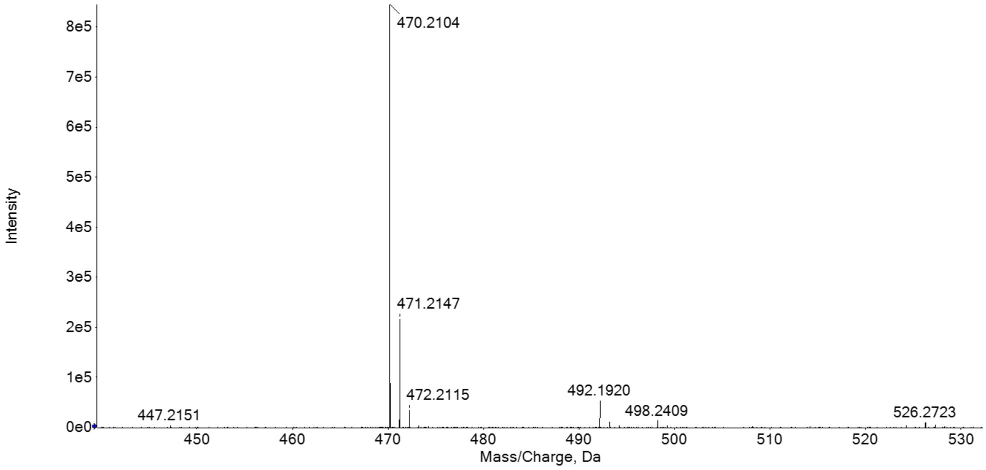

9d:

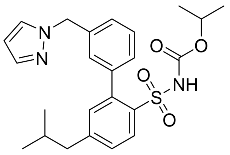

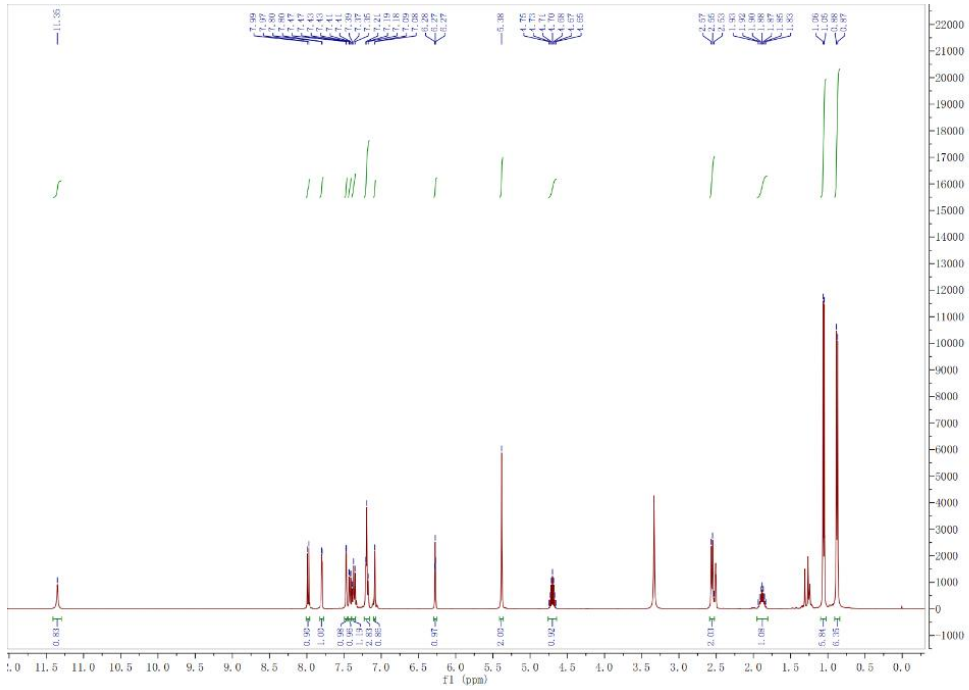
f1 (ppm)

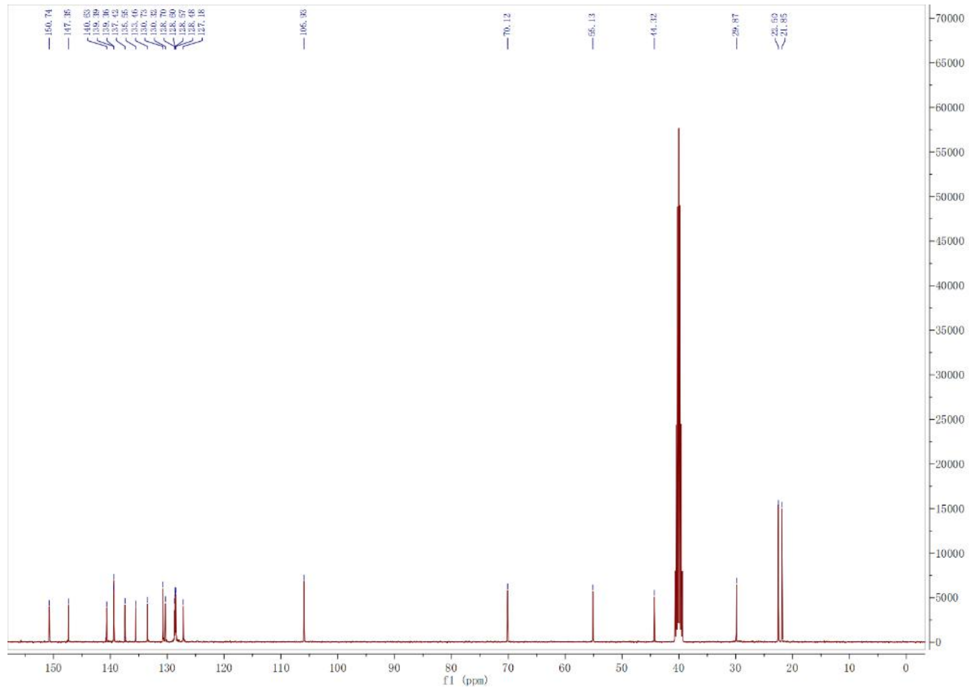
f1 (ppm)

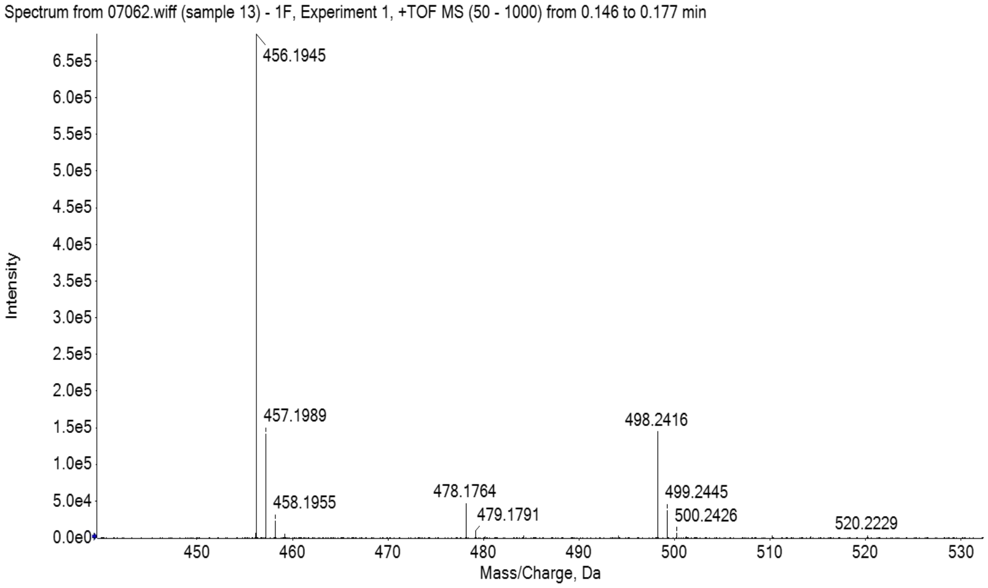
Spectrum from 07062.wiff (sample 13) - 1F, Experiment 1, +TOF MS (50 - 1000) from 0.146 to 0.177 min
456.1945
457.1989
458.1955
478.1764
479.1791
498.2416
499.2445
500.2426
520.2229
Intensity
Mass/Charge, Da

9e:

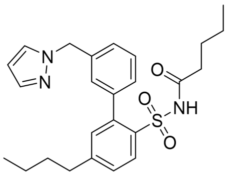

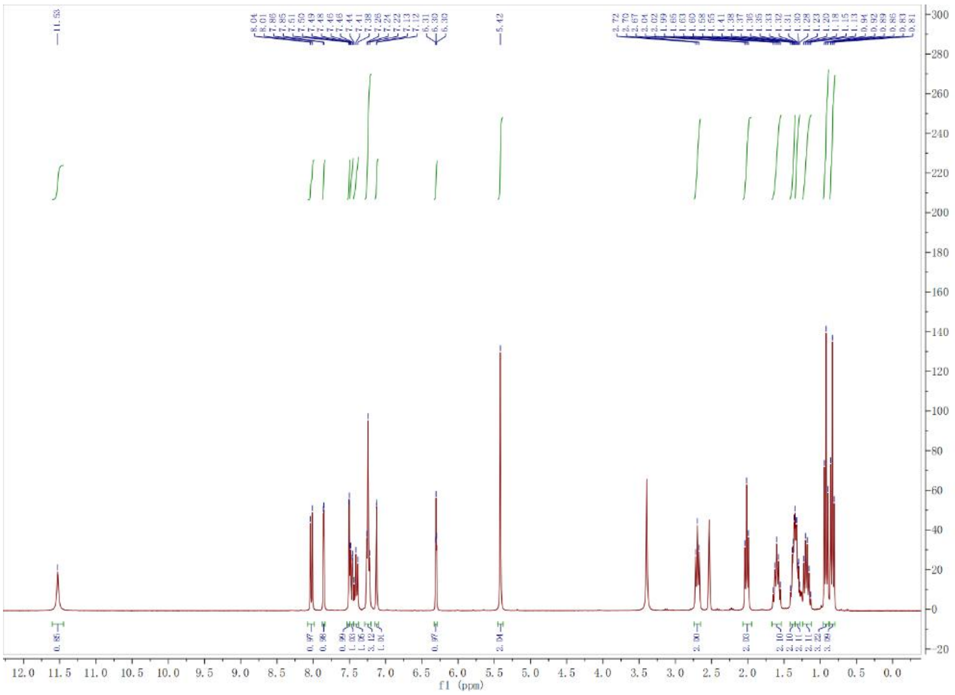
f1 (ppm)

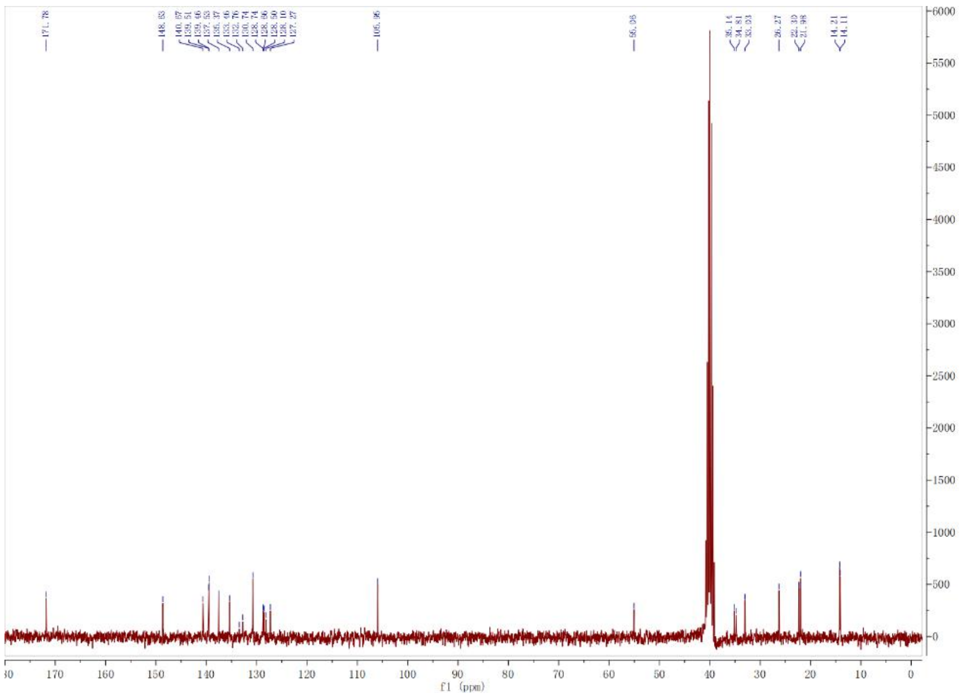

Spectrum from 07062.wiff (sample 16) - 2C, Experiment 1, +TOF MS (50 - 1000) from 0.146 to 0.177 min

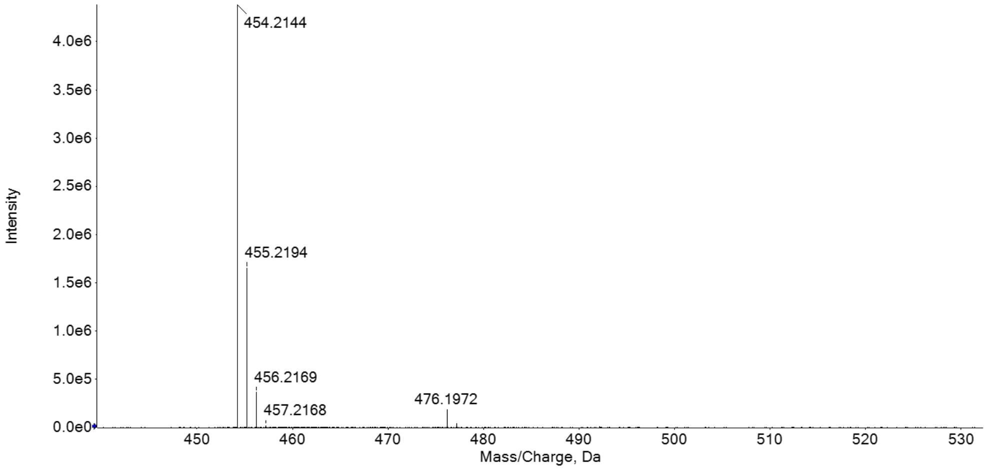

9f:

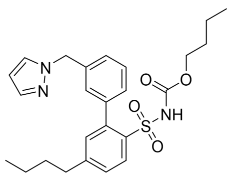

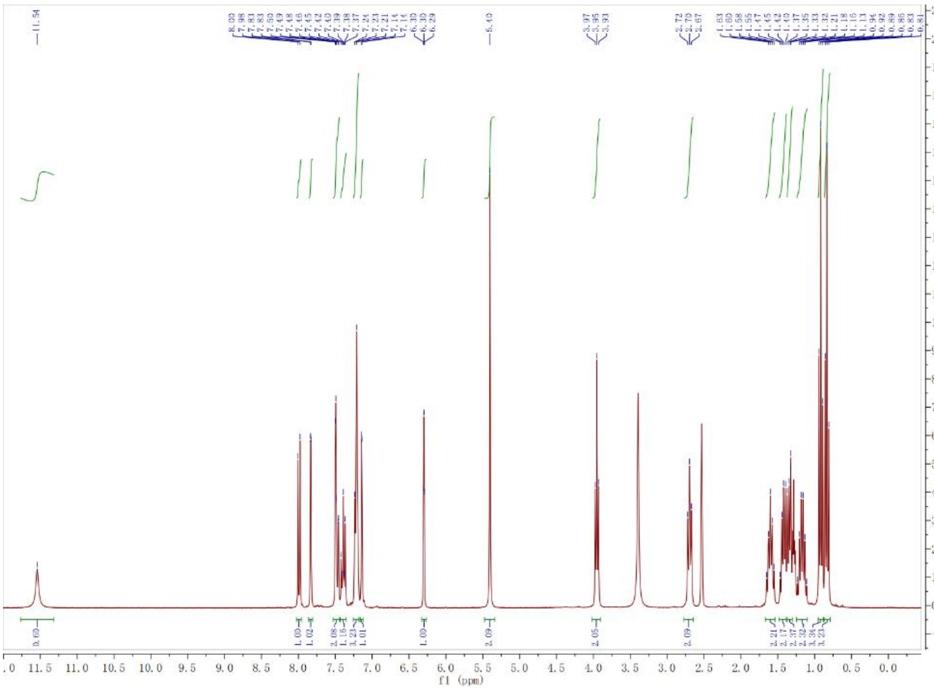
11.54
8.00 7.98 7.83 7.83 7.50 7.49 7.48 7.46 7.45 7.42 7.40 7.39 7.38 7.37 7.24 7.23 7.21 7.14 7.14 6.30 6.30 6.29
5.40
3.97 3.95 3.93
2.72 2.70 2.67
1.63 1.60 1.58 1.55 1.47 1.45 1.42 1.40 1.37 1.35 1.33 1.32 1.21 1.18 1.16 1.13 0.94 0.92 0.89 0.86 0.83 0.81
0.69 1.00 1.02 2.08 1.16 3.23 1.01 1.00 2.09 2.05 2.09 2.21 2.17 2.37 2.32 3.34 3.23
f1 (ppm)

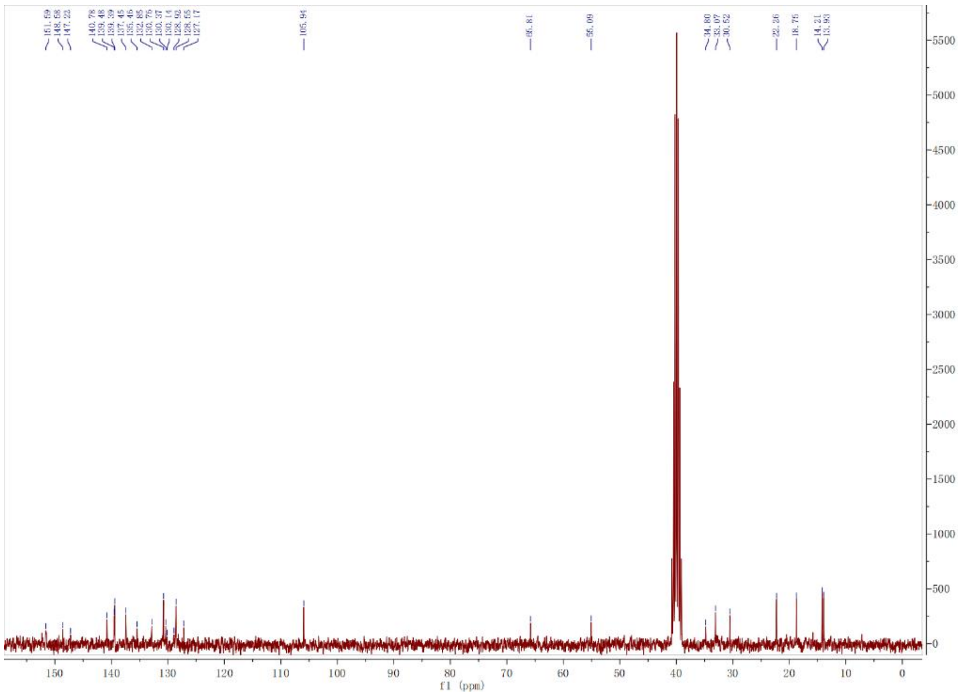
151.59 148.58 147.22 140.78 139.48 139.39 137.45 135.46 132.85 130.75 130.37 130.14 128.92 128.55 127.17
105.94
65.81
55.09
34.80 33.07 30.52
22.26
18.75
14.21 13.93
f1 (ppm)

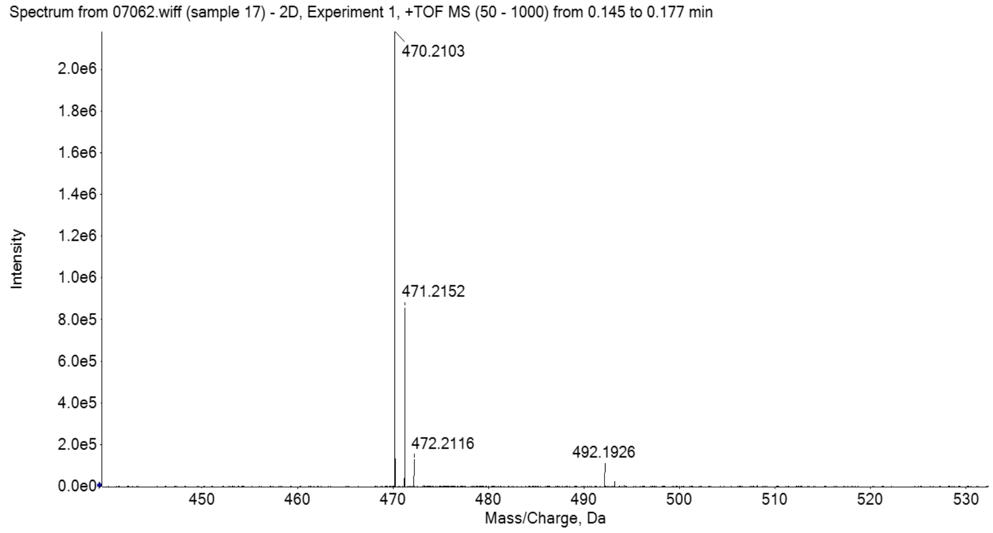
Spectrum from 07062.wiff (sample 17) - 2D, Experiment 1, +TOF MS (50 - 1000) from 0.145 to 0.177 min
470.2103
471.2152
472.2116
492.1926
Intensity
2.0e6
1.8e6
1.6e6
1.4e6
1.2e6
1.0e6
8.0e5
6.0e5
4.0e5
2.0e5
0.0e0
450
460
470
480
490
500
510
520
530
Mass/Charge, Da

9g:

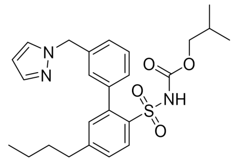
N
N
O
O
NH
S
O
O

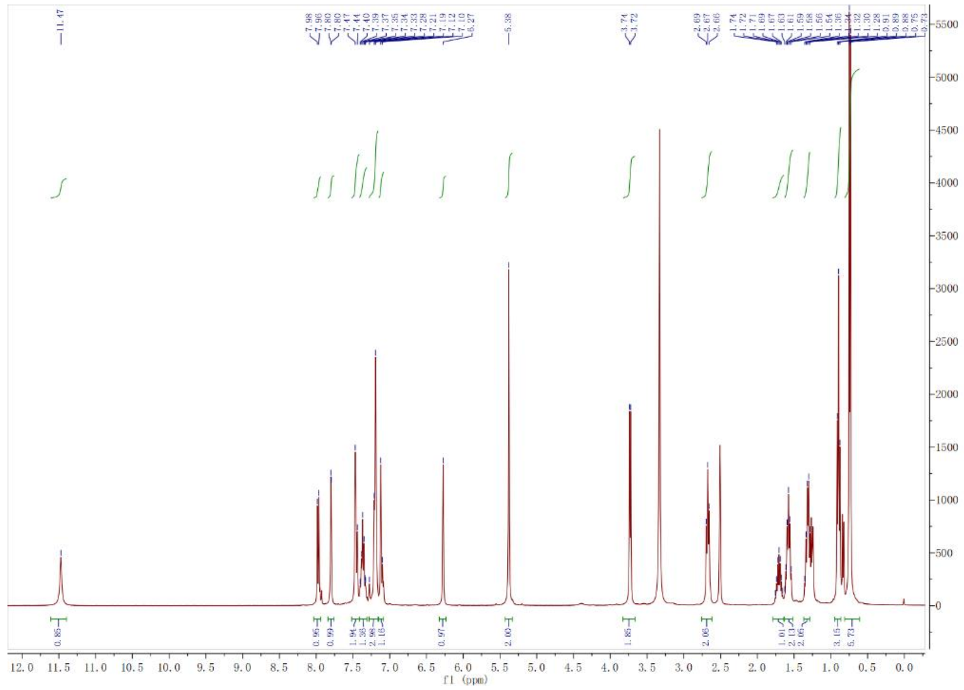
11.47
5.38
3.74
3.72
0.85
0.95
0.99
0.97
2.00
1.85
2.08
3.15
5.73
f1 (ppm)

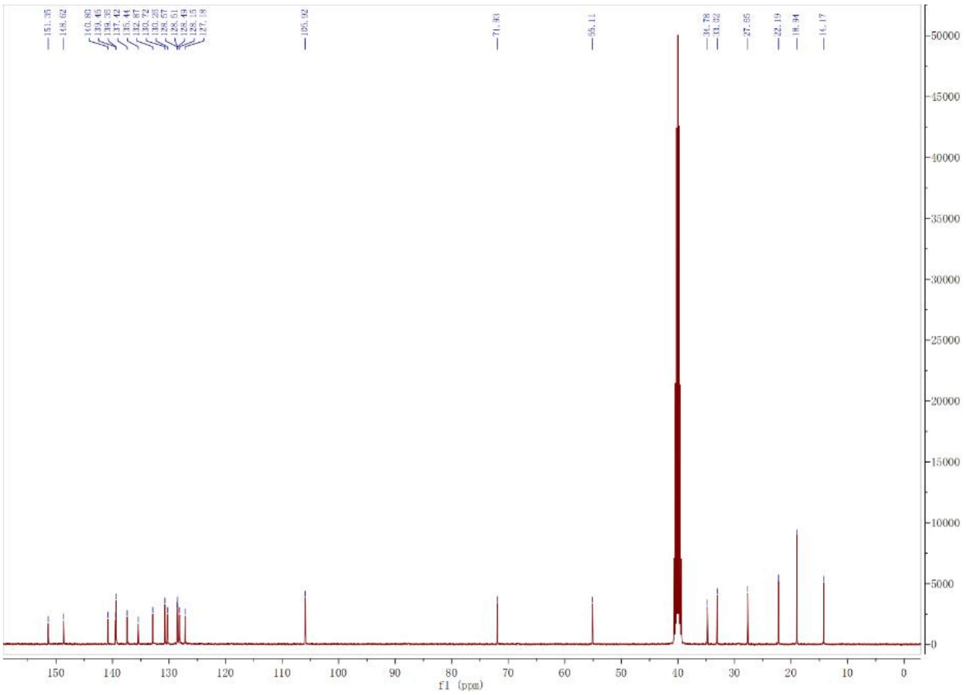
151.35
148.62
140.80
139.46
139.36
137.42
135.44
132.87
130.72
130.28
129.57
128.51
128.49
128.15
127.18
105.92
71.93
55.11
34.78
33.32
27.55
22.19
18.94
14.17
f1 (ppm)

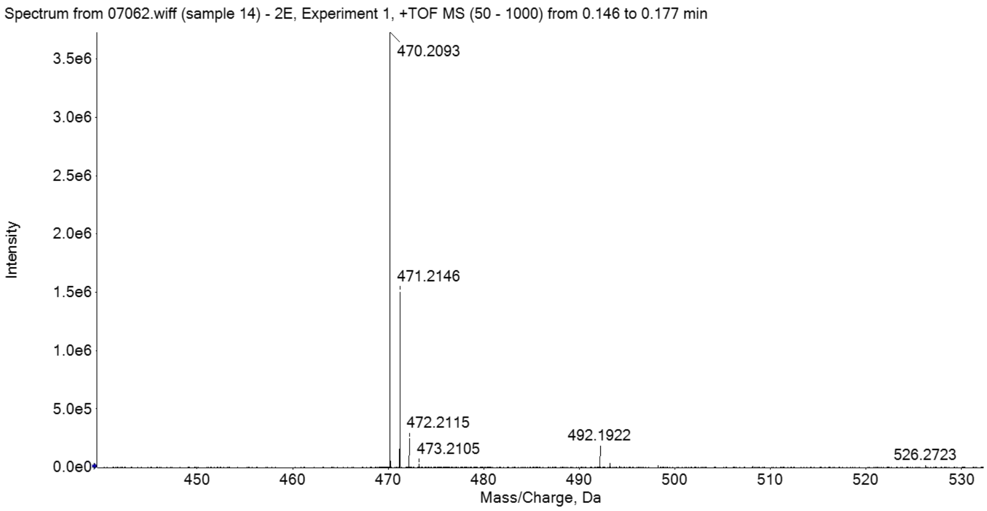
Spectrum from 07062.wiff (sample 14) - 2E, Experiment 1, +TOF MS (50 - 1000) from 0.146 to 0.177 min
470.2093
471.2146
472.2115
473.2105
492.1922
526.2723
Intensity
Mass/Charge, Da

9h:

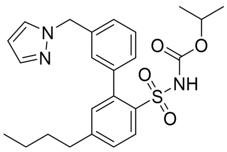
N
N
O
O
NH
S
O
O

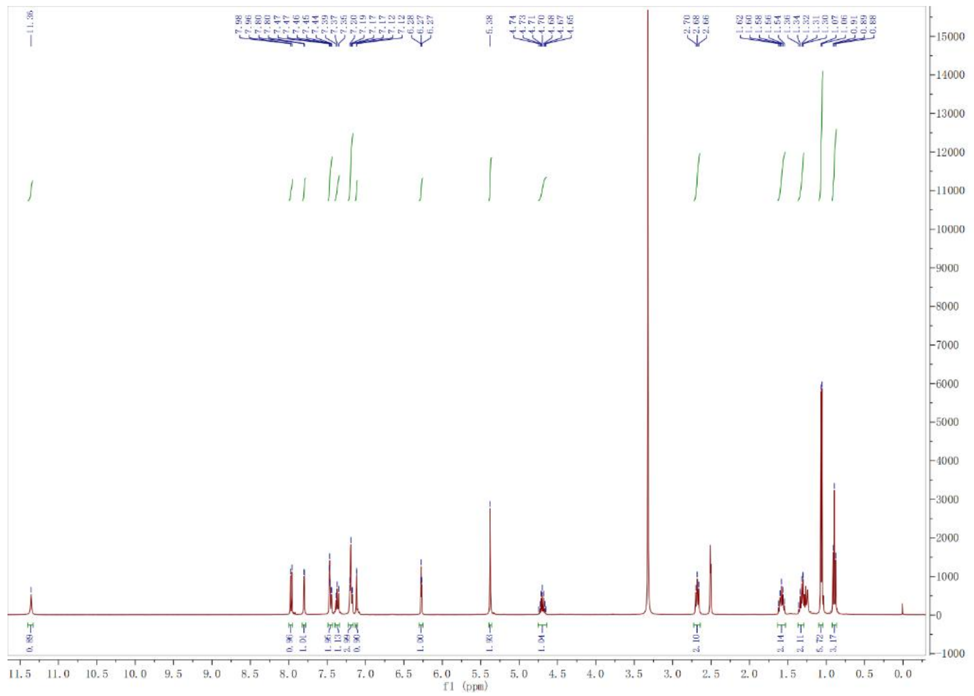

f1 (ppm)

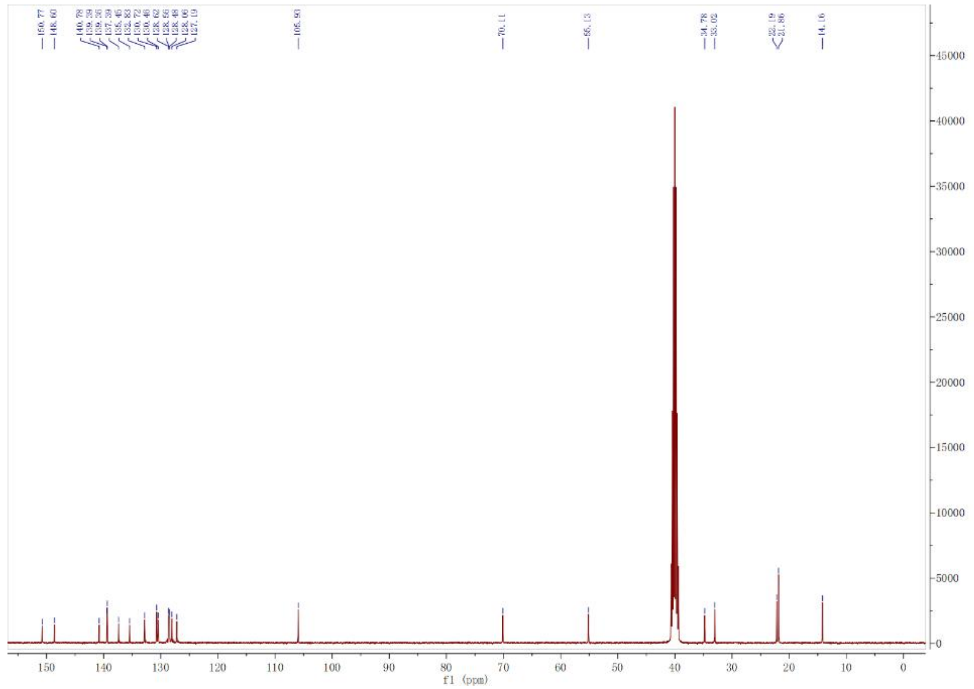

f1 (ppm)

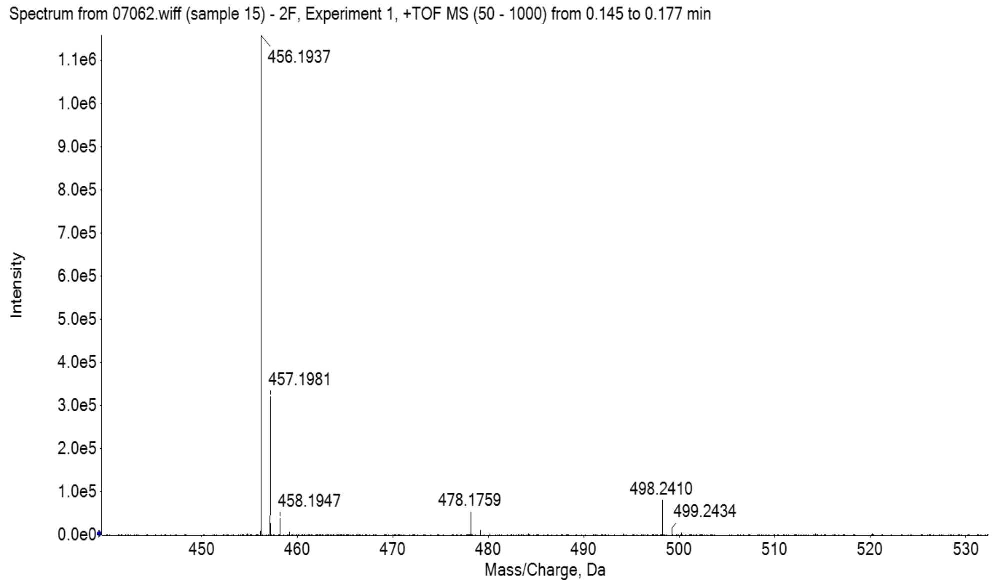

3. Radioligand binding assay steps

3.1 Rat Liver Membrane AT_1 Receptor Binding Assay.

Rat liver membranes were prepared according to the method of Dudley et al[1]. Binding of $[^{125}I]$-Ang II to membranes was conducted in a final volume of 0.5 mL containing 50 mM Tris-HCl (pH 7.4), 100 mM NaCl, 10 mM $MgCl_2$, 1 mM EDTA, 0.025% bacitracin, 0.2% BSA (bovine serum albumin), liver homogenate corresponding to 5 mg of the original tissue weight, $[^{125}I]$-Ang II (80000-85 000 cpm, 0.03 nM), and variable concentrations of test substance. Samples were incubated at 25 °C for 2 h, and binding was terminated by filtration through Whatman GF/B glass-fiber filter sheets, which had been presoaked overnight with 0.3 % polyethylamine, using a Brandel cell harvester. The filters were washed with 3×3 mL of Tris-HCl (pH 7.4) and transferred to tubes. The radioactivity was measured in a γ-counter. The characteristics of the Ang II binding AT1receptor was determined by using six different concentrations (0.03-5 nmol/L) of the labeled $[^{125}I]$-Ang II. Nonspecific binding was determined in the presence of 1 μM Ang II. The specific binding was determined by subtracting the nonspecific binding from the total bound $[^{125}I]$-Ang II. The apparent dissociation constant Ki values were calculated from IC_{50} values using the Cheng-Prusoff equation. The binding data were best fitted with a one-site fit. All determinations were performed in triplicate.

3.2 HEK-293 cell AT_2 Receptor Binding Assay.

After HEK-293 cells were transfected with AT_2 receptor, lysis buffer (150 mM NaCl, 0.1% Triton X-100, 50 mM Tris-HCl, 1 mM EDTA, protease inhibitor) was used to separate the cell membrane of HEK-293 cells[2], using 27-G Resuspend and mix the lysis solution, and centrifuge at 12,000 g for 10 minutes at 4 °C. The supernatant was collected and centrifuged for a further 60 minutes. The precipitated membrane extracts were buffered (150 mM NaCl, 10 mM $MgCl_2$, 0.5 % SDS, 1 % Triton X-100, 50 mM Tris-Cl, 1 mM EDTA, protease inhibitor) for resuspension. Binding of [^{125}I]-CGP42112A to membranes was conducted in a final volume of 0.5 mL containing 50 mM Tris-HCl (pH 7.4), 100 mM NaCl, 10 mM $MgCl_2$, 1 mM EDTA, 0.025 % bacitracin, 0.2 % BSA (bovine serum albumin), cell homogenate corresponding to 5 mg of the original tissue weight, [^{125}I]-CGP42112A (80000-85 000 cpm, 0.03 nM), and variable concentrations of test substance. Samples were incubated at 25 °C for 2 h, and binding was terminated by filtration through Whatman GF/B glass-fiber filter sheets, which had been presoaked overnight with 0.3 % polyethylamine, using a Brandel cell harvester. The filters were washed with 3×3 mL of Tris-HCl (pH 7.4) and transferred to tubes. The radioactivity was measured in a γ-counter. The characteristics of the Ang II binding AT_1 receptor was determined by using six different concentrations (0.03-5 nmol/L) of the labeled [^{125}I]-CGP42112A. Nonspecific binding was determined in the presence of 1 μM Ang II. The specific binding was determined by subtracting the nonspecific binding from the total bound [^{125}I]-CGP42112A. The apparent dissociation constant *Ki* values were calculated from IC_{50} values using the Cheng-Prusoff equation. The binding data were best fittedwith a one-site fit. All determinations were performed in triplicate.

4. NG108-15 cell experiment steps

We have previously shown that NG108-15 cells in their undifferentiated state express only the AT_2 receptor and that a 3-day treatment with **Ang II** or the selective peptidic AT_2 receptor agonist **CGP-42112A** induces neurite outgrowth[3,4]. The signaling pathways involve a sustained increase in Rap1/BRaf/p42/p44^{mapk} activity and activation of the nitric oxide/guanylyl cyclase/cGMP pathway[5-7]. Cells were plated as described in the Experimental Section, and adequate test concentrations for

each compound were determined by testing a dilution series of each compound ranging from 1 pM to 1 uM. For all the compounds it was only at the highest concentration that any evidence of cell death was observed. Antagonistic effect was verified through co-incubation with **Ang II** resulting in reduced Ang II-induced neurite outgrowth, verifying blockage of the AT_2 receptor. Agonistic effect was verified through co-incubation with the selective AT_2 receptor antagonist **PD-123,319**, which reduced neurite outgrowth, verifying that the effect was mediated through the AT_2 receptor. Treatment with **PD-123,319** alone did not alter the morphology compared to untreated cells.

The chemicals used in the present study were obtained from the following sources: Dulbecco's modified Eagle's medium (DMEM), heat-inactivated fetal bovine serum (FBS), HAT supplement (hypoxanthine, aminopterin, thymidine), gentamycin from Gibco BRL (Burlington, Ontario, Canada), and **Ang II** from MedChemExpress (Monmouth Junction, NJ, USA). **PD-123,319** was obtained from MedChemExpress (Monmouth Junction, NJ, USA). All other chemicals were of grade A purity.

For all experiments, cells were plated at the same initial density of 3.6×10^4 cells/35 mm Petri dish. To determine a good test concentration, all compounds were tested at various concentrations ranging from 1 pM to 1 uM. It was only at the highest concentration of compounds **8d** and **8h** that any evidence of cell death was observed, and that was most probably due to a higher concentration of DMSO (due to low solubility). Cells were treated without (control cells), or with **Angiotensin II** (100 nM) or with compound **8d** (100 nM), **8h** (100 nM), **9h** (10 and 100 nM), **8i** (10 and 100 nM), **8j** (100 nM), **8k** (100 nM), or **8l** (10 and 100 nM) in the absence or in the presence of **PD-123,319** (10 uM), an AT_2 receptor antagonist. The antagonist was introduced daily 30 min prior to **Ang II**, compound **8d, 8i, 8k, 8l, 8h** or **9h**, to evaluate antagonistic properties.

Cells were examined under a phase contrast microscope, and micrographs were taken after 3 days under the various experimental conditions. Cells with at least one neurite longer than a cell body were counted as positive for neurite outgrowth. The number of cells with neurites represents the percentage of the total amount of cells in

the micrographs. At least three different experiments were conducted for each condition, each in duplicate[33]. At least five images were taken per petri dish. Hence, a total of 250−400 cells from each of the duplicate dishes were examined.

References

[1] Dudley DT, Panek RL, Major TC, et al. Subclasses of angiotensin II binding sites and their functional significance [J]. *Mol Pharmacol*, 1990, 38 (3): 370-377.

[2] Grieger JC, Soltys SM, Samulski RJ. Production of Recombinant Adeno-associated Virus Vectors Using Suspension HEK293 Cells and Continuous Harvest of Vector From the Culture Media for GMP FIX and FLT1 Clinical Vector [J]. *Molecular Therapy*, 2016, 24 (2): 287-296.

[3] Buisson B, Bottari SP, Gasparo MD, et al. The angiotensin AT_2 receptor modulates T-type calcium current in non-differentiated NG108-15 cells [J]. *Febs Letters*, 1992, 309 (2): 1-9.

[4] Gasparo, DM. Angiotensin II induction of neurite outgrowth by AT2 receptors in NG108-15 cells. Effect counteracted by the AT1 receptors [J]. *J Biol Chem*, 1996, 271 (37): 22729-22735.

[5] Gendron, L. Cyclic AMP-independent involvement of Rap1/B-Raf in the angiotensin II AT2 receptor signaling pathway in NG108-15 cells [J]. *J Biol Chem*, 2003, 278 (6): 3606-3614.

[6] Gendro Louis, Laflamme Liette, Nathalie R, et al. Signals from the AT_2 (angiotensin type 2) receptor of angiotensin II inhibit p21ras and activate MAPK (mitogen-activated protein kinase) to induce morphological neuronal differentiation in NG108-15 cells [J]. *Mol. Endocrinol*. 1999, 13, 1615-1626.

[7] Gendron, Louis, Cote, et al. Nitric Oxide and Cyclic GMP Are Involved in Angiotensin II AT[sub 2] Receptor Effects on Neurite Outgrowth in NG108-15 Cells [J]. *Neuroendocrinology*, 2002, 75, 70-81.
